# Chemoselective Esterification of Natural and Prebiotic 1,2-Amino Alcohol Amphiphiles in Water

**DOI:** 10.1021/jacs.3c12038

**Published:** 2023-12-01

**Authors:** Ahanjit Bhattacharya, Lalita Tanwar, Alessandro Fracassi, Roberto J. Brea, Marta Salvador-Castell, Satyam Khanal, Sunil K. Sinha, Neal K. Devaraj

**Affiliations:** † Department of Chemistry and Biochemistry, University of California, San Diego, La Jolla, California 92093, United States; ‡ Biomimetic Membrane Chemistry (BioMemChem) Group, Centro de Investigacións Científicas Avanzadas (CICA), Universidade da Coruña, Rúa As Carballeiras, 15701, A Coruña, Spain; § Department of Physics, University of California, San Diego, La Jolla, California 92093, United States

## Abstract

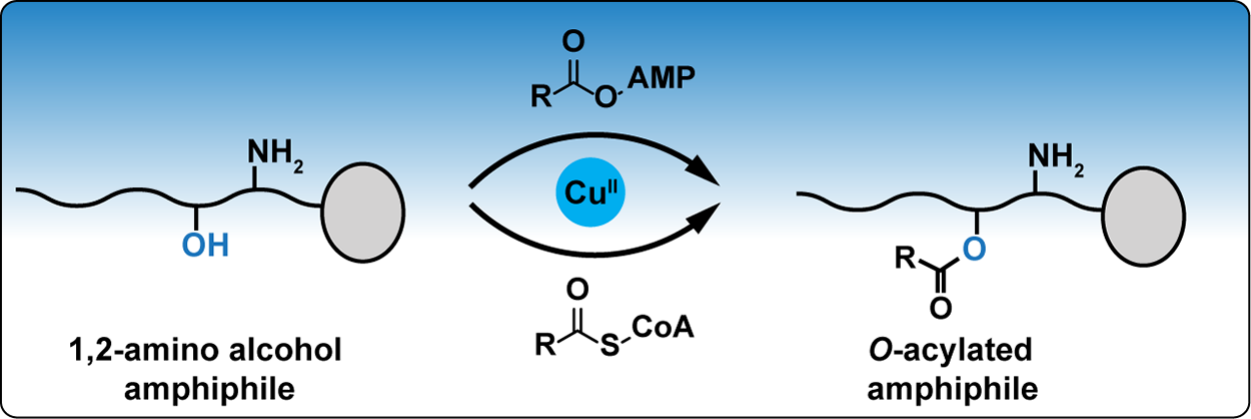

In cells, a vast number of membrane lipids are formed by the enzymatic O-acylation of polar head groups with acylating agents such as fatty acyl-CoAs. Although such ester-containing lipids appear to be a requirement for life on earth, it is unclear if similar types of lipids could have spontaneously formed in the absence of enzymatic machinery at the origin of life. There are few examples of enzyme-free esterification of amphiphiles in water and none that can occur in water at physiological pH using biochemically relevant acylating agents. Here we report the unexpected chemoselective O-acylation of 1,2-amino alcohol amphiphiles in water directed by Cu(II) and several other transition metal ions. In buffers containing Cu(II) ions, mixing biological 1,2-amino alcohol amphiphiles such as sphingosylphosphorylcholine with biochemically relevant acylating agents, namely, acyl adenylates and acyl-CoAs, leads to the formation of the O-acylation product with high selectivity. The resulting O-acylated sphingolipids self-assemble into vesicles with markedly different biophysical properties than those formed from their N-acyl counterparts. We also demonstrate that Cu(II) can direct the O-acylation of alternative 1,2-amino alcohols, including prebiotically relevant 1,2-amino alcohol amphiphiles, suggesting that simple mechanisms for aqueous esterification may have been prevalent on earth before the evolution of enzymes.

## Introduction

**Figure 1:**
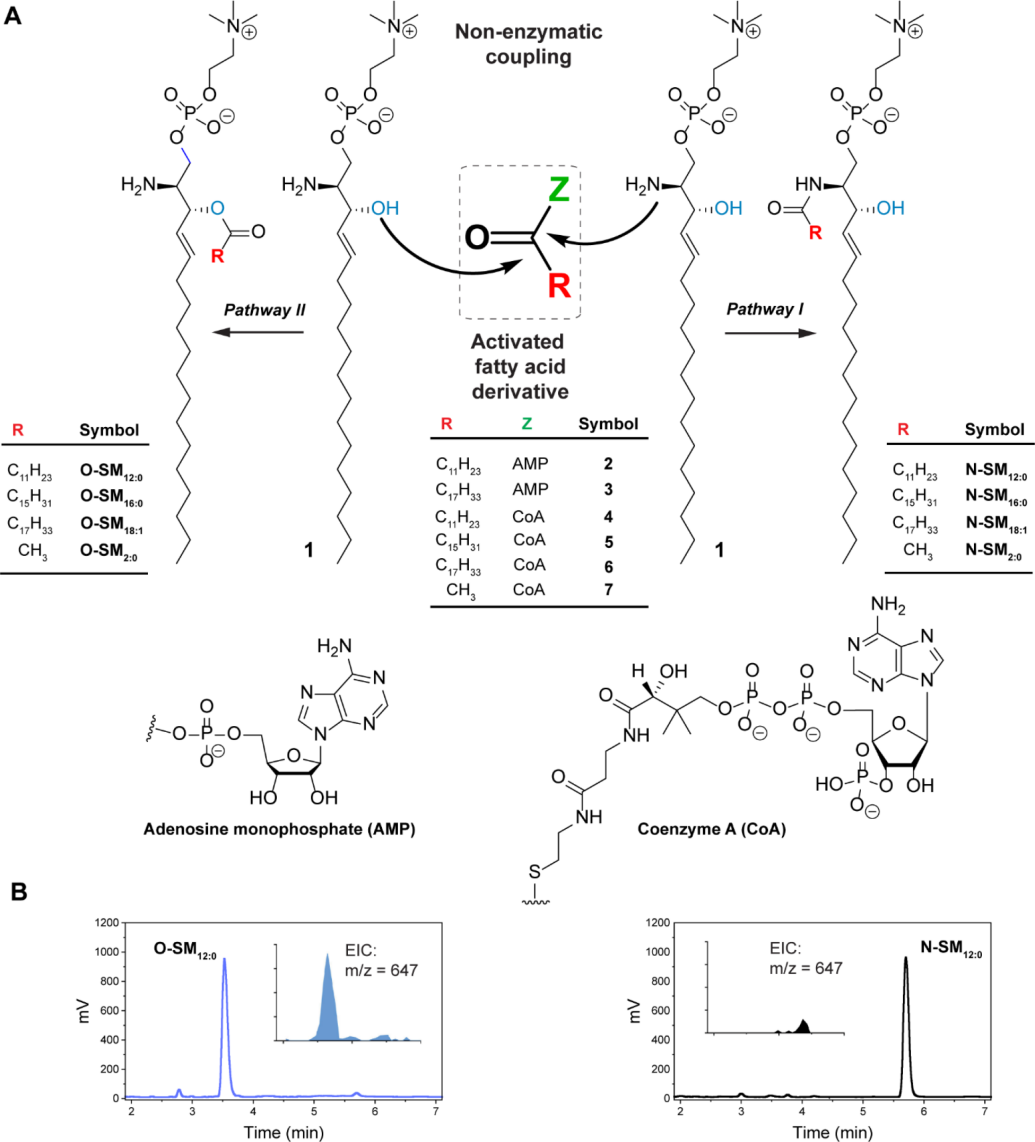
Acylation of a model natural 1,2-amino alcohol amphiphile, sphingosylphosphorylcholine. (A) Reaction schemes showing the enzyme-free acylation of sphingosylphosphorylcholine (**1**) with miscellaneous activated fatty acid derivatives (adenylates and CoA thioesters). (B) The HPLC-ELSD chromatograms corresponding to the synthesis of **O-SM**_**12:0**_ and **N-SM**_**12:0**_ are shown with extracted ion chromatograms (EICs) corresponding to *m*/*z* = 647 in the inset.

Macromolecular catalysis is a central feature of cellular life. In modern cells, protein-based enzymes and ribozymes catalyze most chemical transformations. However, it is unlikely that such sophisticated catalytic machinery existed at the origin of life. Therefore, the earliest biochemical transformations may have been driven by physical phenomenon, like evaporation, or catalyzed by simple chemical species such as metal ions.^1^ It has been suggested that transition metal catalysis played a significant role in prebiotic chemistry.^2^ Based on the analysis of multibillion-year-old rocks like amphibolite and migmatite, it has been estimated that the concentration of metals like copper and iron in the earth’s primordial ocean was significant (∼10–100 μM) enough to have influenced catalytic transformations.^3^ In biological membranes, membrane-bound acyltransferase enzymes catalyze the transfer of a fatty acyl chain to an alcohol or amine group on a single-chain amphiphile to generate lipid species such as diacyl phospholipids, which make up the majority of cell membranes. However, it is unclear whether similar acylation reactions could have proceeded in the absence of enzymes at the origin of life. In particular, the nonenzymatic O-acylation of single-tail amphiphiles would be of great interest because ester linkages are a key feature of the most abundant phospholipids. However, there are only a few examples of nonenzymatic esterification in water. For example, Kluger and co-workers had reported the lanthanide-promoted monoacylation of simple diols using acyl phosphates in aqueous media.^4^ Recently, Liu *et al*. reported that it is possible to carry out nonenzymatic esterification of lysophospholipids directed by ion-pair interactions between amphiphile headgroups.^5^ However, this reaction took place only under significantly alkaline conditions (pH > 9) and required the use of abiotic acylating agents. Here we report that several d-block metal ions, particularly Cu(II), catalyze the selective O-acylation of the 1,2-amino alcohol moiety of the biologically occurring single-chain amphiphile sphingosylphosphorylcholine^6,7^ (also known as lysosphingomyelin) using fatty acyl phosphates (adenylates) or thioesters (acyl-CoAs) as acyl donors under mild aqueous conditions at or near neutral pH to generate O-acylated sphingomyelin analogues (Figure 1A). This transformation is a rare example of selective O-acylation in the presence of a more nucleophilic amine. There are a few reports of metal-directed regioselectivity switches between acylation sites (i.e., preference of alcohol over amine) in organic solvents like toluene and diisopropyl ether, often under harsh conditions like high temperature over prolonged periods.^8−10^ However, to our knowledge, there are no examples of nonenzymatic regioselectivity switches that occur in mild aqueous biochemically relevant conditions. In addition, acyl thioesters are not known to spontaneously react with either amines or alcohols due to their kinetic inertness. Thus, the Cu(II)-catalyzed O-acylation of 1,2-amino alcohol amphiphiles by acyl thioesters can be likened to an enzymatic transformation.

Although N-acylated sphingomyelins are ubiquitously found in eukaryotic membranes, our method of chemoselective esterification leads to the generation of O-acylated analogues of sphingomyelin, which have not been reported previously (Supplementary Note, Figure S1). It is possible that similar O-acylation reactions may take place in living organisms and the corresponding O-acylated molecular species have unknown biological significance. We demonstrate that the O-acylated sphingomyelin analogues self-assemble into vesicles having markedly different physical properties as compared to the vesicles formed from isomeric N-acylated sphingomyelins. We further show that our approach of metal ion-directed selective acylation of 1,2-amino alcohol amphiphiles is not limited to sphingolipid species but can be extended to nonbiological molecules as well. For instance, fingolimod, an amphiphilic FDA-approved drug molecule, can be selectively O-acylated with fatty acyl-CoAs, a modification that may be of interest in medicinal chemistry for generating prodrugs. Finally, we show that a prebiotically plausible single-chain 1,2-amino alcohol amphiphile can be selectively O-acylated to generate a two-tailed amphiphile. Related transformations may have been highly significant in the chemical evolution of lipids at the origin of life.^11^

## Experimental Section

### General Considerations

1-Palmitoyl-2-oleoyl-*sn*-3-phosphocholine (POPC) was obtained from Avanti Polar Lipids. d-*erythro*-lysosphingomyelin (d18:1), l-*threo* lysosphingomyelin (d18:1), and fingolimod HCl were purchased from Cayman Chemicals. Di-*tert*-butyl dicarbonate, 2,4,6-trichlorobenzoyl chloride (TCBC), 4-dimethylaminopyridine (DMAP), *O*-(7-azabenzotriazol-1-yl)-1,1,3,3-tetramethyl-uronium hexafluorophosphate (HATU), adenosine 5′-monophosphate monohydrate (5′-AMP·H_2_O), *N*,*N*-diisopropylethylamine (DIPEA), trifluoroacetic acid (TFA), triethylamine, dodecanoic acid, palmitic acid, oleic acid, palmitoyl-CoA lithium salt, oleoyl-CoA lithium salt, 1,6-diphenyl-1,3,5-hexatriene (DPH), dichloromethane (CH_2_Cl_2_), and *N,N*-dimethylformamide (DMF) were obtained from Sigma-Aldrich. Texas Red 1,2-dihexadecanoyl-*sn*-glycero-3-phosphoethanolamine, triethylammonium salt (Texas Red DHPE), Nile Red, and Alexa Fluor 488 succinimidyl ester were obtained from Life Technologies. Deuterated chloroform (CDCl_3_), DMSO (*d*_6_-DMSO), and methanol (CD_3_OD) were obtained from Cambridge Isotope Laboratories. Proton nuclear magnetic resonance (^1^H NMR) spectra were recorded on a Varian VX-500 MHz spectrometer and were referenced relative to residual proton resonances in CDCl_3_ (at δ7.24 ppm). Chemical shifts were reported in parts per million (ppm, δ) relative to tetramethylsilane (δ 0.00). ^1^H NMR splitting patterns were assigned as singlet (s), doublet (d), triplet (t), quartet (q), or pentuplet (p). All first-order splitting patterns were designated on the basis of the appearance of the multiplet. Splitting patterns that could not be readily interpreted are designated as multiplet (m) or broad (br). Carbon nuclear magnetic resonance (^13^C NMR) spectra were recorded on a Varian VX-500 MHz spectrometer and were referenced relative to residual proton resonances of CDCl_3_ (at δ77.23 ppm). Phase-contrast images were acquired on an Olympus BX51 upright microscope. Spinning-disk confocal microscopy images were acquired on an Axio Observer Z1 motorized inverted microscope (Carl Zeiss Microscopy GmbH, Germany) equipped with a Yokagawa spinning-disk system (Yokagawa, Japan) and 63×, 1.40 NA oil immersion objective. Images were captured using an Orca Flash 4.0 CMOS camera (Hamamatsu) using the ZEN imaging software (Carl Zeiss Microscopy GmbH, Germany). A NanoDrop 2000C spectrophotometer was used for UV/vis measurements. Fluorescence anisotropy measurements were carried out on a Cary Eclipse Fluorescence Spectrophotometer (Agilent Technologies).

### High-Performance Liquid Chromatography–Mass Spectrometry (HPLC–MS) Methods

HPLC analyses were carried out on an Agilent 1260 Infinity LC System. All analytical separations were carried out using an Eclipse Plus C8 analytical column at a flow rate of 1.0 mL/min. Detection was done using a diode array detector (DAD) and an evaporative light scattering detector (ELSD). Use of ELSD allowed sensitive detection of lipids with a linear response to concentration over a broad range. HPLC purification was carried out on a Zorbax SB-C18 semipreparative column at a flow rate of 4.0 mL/min. Various ratios of Phase A (H_2_O with 0.1% v/v formic acid) and Phase B (MeOH with 0.1% v/v formic acid) were used as the mobile phase. Electrospray ionization time-of-flight (ESI-TOF) spectra were obtained on an Agilent 6230 Accurate-Mass TOF-MS mass spectrometer.

### General Procedure for Cu(II)-Directed O-Acylation in Aqueous Media

#### If the Starting Material Is Soluble in Water

To a 2 mL Eppendorf tube, 1,2-amino alcohol (10 mM in H_2_O, 4 μL) was added followed by HEPES-Na (1 M, pH 7.5, 2 μL), H_2_O (28 μL), CuSO_4_·5H_2_O (10 mM in H_2_O, 2 μL), and an acylating agent (10 mM in H_2_O or buffer, 4 μL). The reaction mixture was vortexed for 10 s and tumbled at 37 °C. To analyze the formation of products, 10 μL of the reaction mixture was dissolved in MeOH and injected into LC-MS.

#### If the Starting Material Is Insoluble in Water

To a 2 mL Eppendorf tube, 1,2-amino alcohol (10 mM in MeOH/CHCl_3_, 4 μL) was added, and the organic solvents were evaporated under N_2_ flow. After drying for 10 min, HEPES-Na (1 M, pH 7.5, 2 μL), H_2_O (32 μL), CuSO_4_·5H_2_O (10 mM in H_2_O, 2 μL), and acylating agent (10 mM in H_2_O or buffer, 4 μL) were added. The reaction mixture was vortexed for 10 s and tumbled at 37 °C. To analyze the formation of products, 10 μL of the reaction mixture was dissolved in MeOH and injected into LC-MS.

### X-ray Diffraction (XRD) Studies on Lipid Multilayers

#### Preparation of Lipid Multilayers and Data Acquisition

XRD experiments were performed on multistacks of oriented lipid bilayers deposited on freshly cleaned hydrophilic silicon [100] wafers. Silicon substrates, cut to 18 × 20 mm, were sonicated three times for 15 min in methanol followed by another 15 min in deionized water (18 MΩ cm^–1^, Milli-Q; Millipore, Billerica, MA). Substrates were then nitrogen-dried and exposed to short-wavelength UV radiation for 30 min to make the surface hydrophilic. For lipid deposition, the wafers were placed on an accurately leveled platform, and 2 μmol of the corresponding lipid was deposited dropwise. The wafers were left for about 2 h covered at the fume hood for slow evaporation and then placed under high vacuum for 24 h to completely evaporate the solvents. The dried lipid films were equilibrated under 97% relative humidity (RH) at 50 °C for 48 h, and finally, they were equilibrated for 24 h at room temperature under different RHs (98, 93.5, 83, and 75%) achieved by a reservoir of different saturated salt solutions (K_2_SO_4_, KNO_3_, KCl, and NaCl, respectively).^12^ XRD measurements were carried out using an in-house Cu Kα tube spectrometer with a wavelength of 1.54 Å operating in the horizontal plane. During the in-house XRD measurements, we used a previously reported humidity cell designed for high accuracy and sensitivity in RH.^13^ The scattering intensity *I*(*q*) was plotted as a function of *q* (intensity of scattering vector), which is directly related to the scattering angle by *q* = 4π sin(θ)/λ, where λ is the X-ray wavelength. We obtained one-dimensional *I*(*q*) profiles for each RH with Bragg peaks, indicating the presence of a lamellar phase for both lipids. The diffraction peaks were fitted by a Gaussian after background subtraction. The repeat distance (or *d*-spacing) of the lamellar phase was calculated by *D* = 2π/Δ*q*, where Δ*q* corresponds to the slope of a linear fit of peak position (*q*) vs diffraction order (*n*).

#### Calculation of Electron Density Profiles (EDPs)

The integrated intensity *I*_*n*_ of *n*th order peaks were used to calculate the electron density profiles as follows:^14^
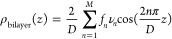
where *D* is the lamellar spacing; coefficient *f_n_* is found by the relationship , where *q*_*z*_ is the Lorentz correction factor equal to *q* for oriented bilayers and *I*_*n*_ is the integrated intensity of the *n*th Bragg peak; and *v_n_* is the phase factor for the *n*th order reflection.^15^ Because of the mirror symmetry of the bilayers in the *z* direction, the phase factors can only be ±1.^16^ For each phase, the intensities of all diffraction orders are normalized by the sum of all peak intensities in that phase to account for the full beam intensity normalization correction. We established the phase factors by following the swelling method,^17^ corresponding to −, −, +, −, + for both lipids at 98% RH. For each phase, intensities of all diffraction orders were normalized by the sum of all peak intensities in that phase to account for the full beam intensity normalization correction. Finally, the distance between the two characteristic maxima of the density profile was attributed to the lipid headgroup to headgroup distance (*D*_hh_), and the water layer thickness between lipid bilayers was defined as *D*_w_ = *D* – *D*_hh_.^18^

### Cryogenic Electron Microscopy (Cryo-EM)

EM grids (Lacey Carbon Film, Electron Microscopy Sciences #LC200-Cu) were glow-discharged (Emitech K350 unit at 20 mA for 30 s), deposited with 4 μL of a 1 mM lipid dispersion, blotted, and then plunged into liquid ethane using a Vitrobot (Mark IV, Thermo Fisher Scientific). Images were acquired on a Talos Arctica (FEI) operated at 200 kV and collected with a total dose of 40 e/Å^2^ at 1.55 Å/pixel and a 3 μm nominal defocus.

## Results and Discussion

### Reactions between Natural 1,2-Amino Alcohol Amphiphiles and Activated Fatty Acid Derivatives

Amphiphilic environments have been shown to facilitate reactions that are otherwise highly inefficient.^5,19^ Amphiphilic reactants can undergo self-assembly into supramolecular aggregates, raising the effective molarity and rendering a reaction chemoselective with minimal interference from nonamphiphilic competing groups.^20,21^ Based on this principle, reactions between functionalized lysolipid fragments and activated fatty acid derivatives have been extensively applied to synthesize membrane compartments for synthetic cells.^22^ Fatty acyl phosphates, such as adenylates, are biochemically important activated fatty acid derivatives that occur transiently in all fatty acid activation pathways in cells.^23^ Acyl phosphates are also thought to be key energy-rich intermediates in prebiotic chemistry.^24,25^ Therefore, fatty acyl adenylates serve as readily accessible model compounds for biological and prebiotic chemistries. Recently, we described a strategy for the synthesis of glycerophospholipids analogues that involved the nonenzymatic coupling between amine-functionalized lysophospholipids and fatty acyl adenylates in aqueous solution.^21^ The resulting amidophospholipids self-assembled into cell-mimetic vesicular compartments. To follow up on these previous N-acylation studies, we began investigating the synthesis of analogues of the natural lipid sphingomyelin via acylation of the NH_2_(C2) group on sphingosylphosphorylcholine (**1**) with dodecanoyl-AMP (**2**) or oleoyl-AMP (**3**) in water (Figure 1A). Indeed, we found using HPLC-ELSD-MS that when **1** and **2** (or **3**) are mixed at pH 7.5 (50 mM HEPES-Na) at room temperature, the expected N-acylated products **N-SM**_**12:0**_ (*m*/*z* = 647, Figure 1A) or **N-SM**_**18:1**_ are obtained (pathway I). This is perhaps not surprising given the known reactivity of amines with acyl adenylates.^26^ However, the reaction was sluggish, likely because the amine group of **1** is situated on a secondary carbon. We therefore decided to explore if metal ions, such as Mg(II) ions, could accelerate the rate of reaction, as previous studies had demonstrated that N-acylation using acyl phosphate reactants could be stimulated by metal additives.^27^ As expected, we found that the rate of the reaction significantly increased in the presence of Mg(II) ions. This is likely due to the greater electrophilicity of the carbonyl carbon of **2** (or **3**) resulting from coordination of the Mg(II) ion to the acyl phosphate moiety of AMP. Inspired by this result, we screened a series of common metal salts to assess their catalytic effect on the acylation reaction. Using HPLC–ELSD–MS to characterize the reaction products between **1** and **2**, we were surprised to observe an additional peak at a lower retention time (compared to **N-SM**_**12:0**_) when the reaction was carried out in the presence of specific metal ions (Figure 1A, pathway II). This peak had an *m*/*z* value of 647, which was identical to that of the N*-*acylation product. As acylation at NH_2_(C2) and acylation at OH(C3) are both possible outcomes, we speculated that the product at the lower retention time (∼3.5 min) corresponded to the O-acylated product (**O-SM**_**12:0**_) as it would be expected to be more polar due to the protonation of the free -NH_2_ group under the eluent solvent conditions (methanol–water containing 0.1% formic acid). Furthermore, the corresponding mass spectra showed a strong molecular ion [M + H^+^] (*m*/*z* = 647) signal, which we thought could be due to the ionization of the amine group (Figure 1B). In comparison, the peak at higher retention time (∼5.7 min), which was previously confirmed to be **N-SM**_**12:0**_, gave a significantly suppressed [M + H^+^] (*m*/*z* = 647) signal (Figure 1B).

To validate that the newly formed product is indeed O-acylated sphingosylphosphorylcholine, we carried out further characterization experiments. We synthesized the compounds **O-SM**_**12:0**_ and **N-SM**_**12:0**_ using standard organic chemistry approaches (Schemes S1 and S2) to verify the retention times in HPLC–ELSD–MS and characterized the structure using NMR spectroscopy. Notably, the ^13^C NMR spectrum of **O-SM**_**12:0**_ displayed a carbonyl peak at 172.4 ppm (ester), whereas that of **N-SM**_**12:0**_ displayed a carbonyl peak at 174.5 ppm (amide) (Figure S2). Infrared (IR) spectroscopy of the isomers showed carbonyl stretching bands at 1718 cm^–1^ (ester) and 1646 cm^–1^ (amide) for **O-SM**_**12:0**_ and **N-SM**_**12:0**_, respectively (Figure S2). Finally, **O-SM**_**12:0**_ presented a significantly smaller peak in the 205 nm chromatogram compared to that from the same amount of **N-SM**_**12:0**_ (Figure S2) due to the smaller molar absorptivity of ester groups compared to that of amides.

Among the metal ions we screened, the hard cations (entries 1–4, Table 1) promote N-acylation exclusively, whereas relatively soft or borderline cations (entries 5–11, Table 1) promote O-acylation to various degrees. A summary of the outcomes of the reactions between **1** and **2** in the presence of miscellaneous water-soluble metal salts at pH 7.5 is provided in Table 1. The ratios between **O-SM**_**12:0**_ and **N-SM**_**12:0**_ products were estimated based on standard calibration curves generated from HPLC peak areas (205 nm chromatogram). We reason that for O-acylation to take place, the OH(C3) group is deprotonated upon coordination to the catalytic metal ion, which makes it more nucleophilic compared to NH_2_(C2), and therefore, attack of the carbonyl group of **2** by OH(C3) is favored.^28^ Among all the cations screened, Cu(II) was found to promote O-acylation with the highest selectivity (∼95%) (entries 8–9, Table 1). We tested several water-soluble salts (50 mol % with respect to 1 mM each of **1** and **2**) like CuSO_4_, CuCl_2_, and Cu(OAc)_2_ and observed similar product ratios. The selectivity was maintained even when an additional 20 equiv of Mg(II) was added. We obtained 76 and 72% O-acylation when 10 and 2.5 mol % Cu(II) were used, respectively.

**Table 1:** Summary of Metal Ion-Dependent Acylation of a 1,2-amino Alcohol Amphiphile Sphingosylphosphorylcholine (**1**)^a^

entry	species	metal salt	**O-SM**_12:0_:**N-SM**_12:0_
1	Li(I)	LiCl	0:100
2	Mg(II)	MgCl_2_·6H_2_O	0:100
3	Ca(II)	CaCl_2_·2H_2_O	0:100
4	Mn(II)	MnCl_2_	0:100
5	Fe(II)	FeSO_4_·7H_2_O	40:60
6	Co(II)	Co(NO_3_)_2_·6H_2_O	66:34
7	Ni(II)	NiSO_4_·6H_2_O	80:20
8	Cu(II)	CuSO_4_·5H_2_O	95:5
9		Cu(OAc)_2_·2H_2_O	96:4
10	Zn(II)	ZnCl_2_	66:34
11		Zn(OAc)_2_·2H_2_O	73:27

a In each of these reactions, **1** (1 mM) was incubated with **2** (1 mM) in HEPES-Na (100 mM, pH 7.5) in the presence of 10 mM of various metal salts at 37 °C.

**Figure 2:**
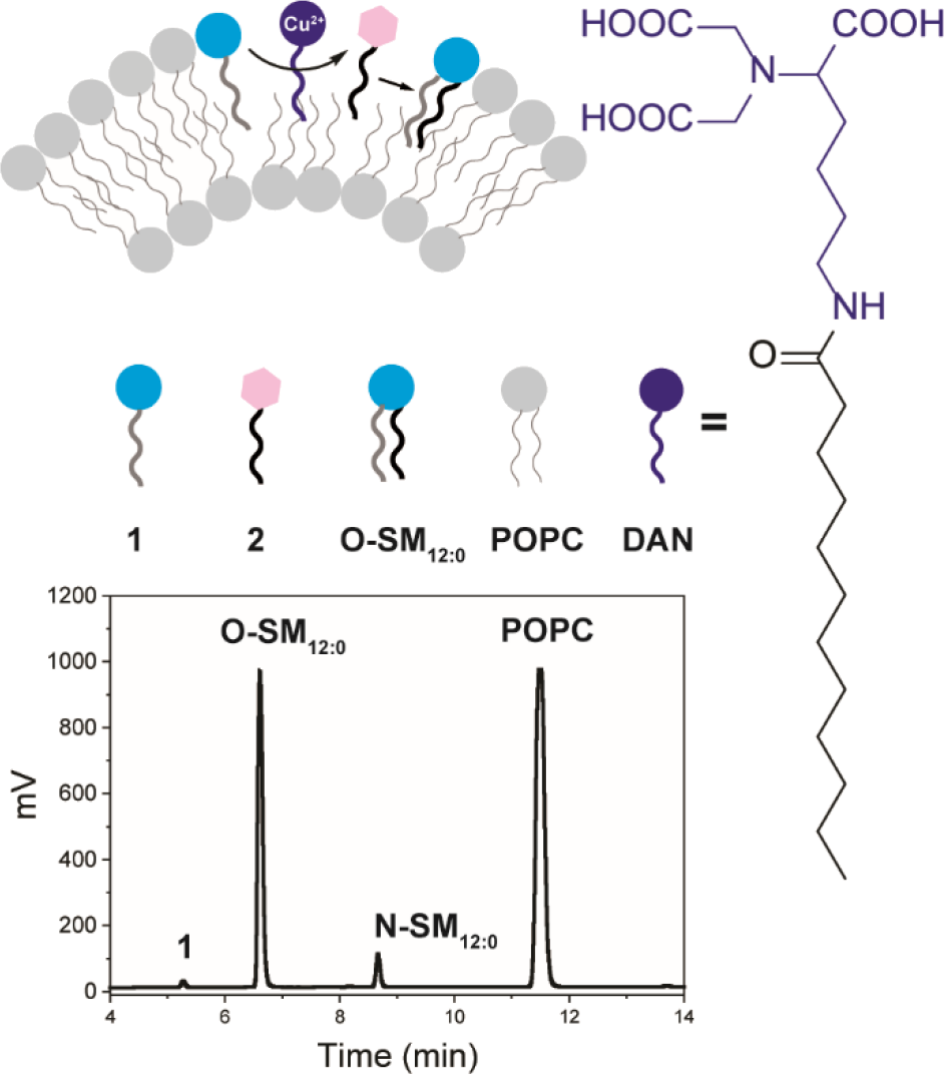
Synthesis of **O-SM**_**12:0**_ via coupling between sphingosylphosphorylcholine (**1**) and dodecanoyl-AMP (**2**) directed by a Cu(II) ion in the presence of an amphiphilic chelating ligand (**DAN**) in phospholipid (POPC) vesicle membranes. HPLC–ELSD chromatogram shows the formation of **O-SM**_**12:0**_ as the major product along with a small amount of **N-SM**_**12:0**_.

Having explored the conditions under which two modes of acylation of **1** take place, we asked if similar reactivity patterns are observed if the reaction is carried out in the presence of copper chelating ligands. Previous work has suggested roles for copper chelating ligands like nitriles and amino acids in the origin of life,^3,29,30^ and in cells, copper is found nearly entirely in its complexed form. Furthermore, it has been suggested that certain amphiphilic lipid species may serve as ligands for complexing Cu(II) inside cells.^31^ We tested whether a model amphiphilic compound dodecanoyl AB-NTA (**DAN**, Scheme S5) chelated with copper can direct the coupling between **1** and **2** in a preformed membrane (Figure 2). We prepared 1-palmitoyl-2-oleoyl-*sn*-glycero-3-phosphocholine (POPC) vesicles embedding **1**, **2**, and Cu-**DAN**. Using HPLC–ELSD–MS, we observed the formation of **O-SM**_**12:0**_ as the majority product (95%) (Figure 2).

In addition to acyl phosphates, thioesters are ubiquitously found in biochemistry as activating groups for carboxylic acids, enabling acylation chemisty.^32^ Thioesters are also thought to have been found in prebiotic environments and have been argued to be key metabolites involved in the origin of life, and their generation by ribozyme catalysis has been explored in an RNA World context.^33,34^ We were curious if Cu(II) species can drive the O-acylation of the 1,2-amino alcohol amphiphile **1** with biochemically relevant and readily available model thioesters such as fatty acyl-CoAs. Whereas acyl adenylates are usually transient species in cells, acyl-CoAs are kinetically stable species that typically require enzymatic catalysis for their use as reactants for O-acylation. We decided to examine if metal catalyzed O-acylation of **1** was feasible with various biologically occurring fatty acyl-CoAs (**4**, **5**, and **6**) as acylating reagents (Figure 1A). Unlike adenylates, which are known to spontaneously acylate amines, acyl-CoAs do not show intrinsic reactivity toward amines even in the presence of Mg(II). Initially, we tested the reaction between **1** (1 mM) and dodecanoyl-CoA (**4**) at pH 7.5 (50 mM HEPES-Na) in the presence of 50 mol% of Cu(II) at 37 °C. After 7 h, we observed the formation of **O-SM**_**12:0**_ with only traces of **N-SM**_**12:0**_ (Figure S3). Next, we carried out the Cu(II)-directed acylation of **1** with palmitoyl-CoA (**5**). After 24 h of metal catalyzed reaction between **1** and **5**, we observed the apparent O-acylated product **O-SM**_**16:0**_ with traces of **N-SM**_**16:0**_ (Figure S4). Similar to that previously described, the HPLC–ELSD chromatogram of the reaction showed two different product peaks with different retention times. Using standard organic synthesis methods, we synthesized O- and N-acylated standards (Scheme S4) and confirmed that the peak at the retention time of 3.6 min corresponds to **O-SM**_**16:0**_ (*m*/*z* = 702) and the peak at 7.3 min corresponds to **N-SM**_**16:0**_. No acylated product was observed without the addition of Cu(II), meaning that Cu(II) plays a crucial role in achieving chemoselective O-acylation. Other fatty acyl-CoAs, such as oleoyl-CoA (**6**), also reacted with **1** to afford **O-SM**_**18:1**_ with traces of **N-SM**_**18:1**_ (Figure S5). Fatty acyl-CoA thioesters are amphiphilic species that would be expected to form mixed micelles with 1,2-amino alcohol **1**. As mentioned, it is likely that coassembly of the amphiphilic reactants is important for the reaction kinetics. To test this, we also looked at the acylation of **1** with acetyl-CoA (**7**), a key metabolite in cells. Interestingly, **7** was also able to acylate **1** in the presence of Cu(II) to obtain **O-SM**_**2:0**_, albeit with more sluggish kinetics. The use of a large excess of **7** was required to achieve comparable yields (Figure S6). Thus, it appears that lipid colocalization does aid the acylation reaction, although it is not a strict requirement.

We also examined the effect of stereochemistry on the outcome of the reactions. The 1,2-amino alcohol moiety on **1** has a d-*erythro* (2S, 3R) configuration, and NH_2_(C2) and OH(C3) are placed *anti* to one another. Acylation of **1** with fatty acyl-CoAs leads primarily to O-acylation with traces of N-acylation. However, when we performed the reaction between the corresponding l-*threo* (2S, 3S) isomer **1′**and **5** at pH 7.5, an approximately 1:1.5 ratio between O- and N-acylated products was obtained after 5 h (Figure S7). After 24 h, the reaction mainly afforded an N-acylated product (Figure S7). The NH_2_(C2) and OH(C3) are placed *syn* to one another in the l-*threo* isomer, and thus, a likely explanation for these results is that intramolecular *O* → *N* acyl transfer is facilitated when compared to the d-*erythro* isomer.^35^ This observation led us to examine the effect of the pH on the outcome of Cu(II)-catalyzed acylation of **1**. We found that over a pH range of 5–8, copper catalyzed acylation of **1** with adenylate **2** leads to nearly exclusive O-acylation (Figure S8A). At pH 9, the product ratio was completely reversed, and **N-SM**_**12:0**_ was the majority product. We reason that, at pH 9, which is considerably close to the expected p*K*_a_ of the free NH_2_(C2) group, the latter is nucleophilic enough to cause facile *O* → *N* acyl transfer reaction.^36^ Comparable to the adenylates, we observed a similar trend of product ratio when the reaction was carried out between **1** and **4** at different pH values over the range 5–9 (Figure S8B). At pH 5.4, exclusively **O-SM**_**12:0**_ was synthesized, and at pH 9.0, the majority of the product was **N-SM**_**12:0**_.

Selective esterification of an alcohol in the presence of an amine is an inherently challenging reaction because of the higher intrinsic nucleophilicity of the amine group. Previous synthetic methodologies have relied on a protective strategy in which an amine and an adjacent carbonyl group are cochelated using Cu(II) to restrict acylation to a remote alcohol center using a highly reactive acylating agent such as an acyl chloride.^37^ However, there are several lines of evidence that suggest that, in our observed reaction, the acylation of alcohol is taking place not merely due to blocking of the amine’s reactivity. First, we observe that when adenylate **2** is added to the C_14:0_ lysolipid (which lacks any amine) in the presence of Cu(II), only traces of O-acylation take place (Scheme S7A). Furthermore, no acylation of the alcohol group on the C_14:0_ lysolipid takes place using thioester **4** as the acylating agent in the presence of Cu(II). In addition, we observe that when an N-acyl sphingomyelin like **N-SM**_**16:0**_ is combined with acylating agents **2** or **4** in the presence of Cu(II), no O-acylation on the free OH(C3) takes place (Scheme S7B). The above results imply that both the amine and alcohol are required for coordination to the Cu(II) center to achieve chemoselective O-acylation of the 1,2-amino alcohol moiety. The OH(C3) group on **1** is likely deprotonated to alkoxide upon coordination to the catalytic Cu(II) ion, making the site more nucleophilic compared to the coordinated NH_2_(C2). Therefore, attack of the carbonyl group of either adenylates or thioesters by OH(C3) is favored. It is notable that the Tonellato group has demonstrated through many examples that aminoalkoxide nucleophiles coordinated to a catalytic metal center mimic the functions of hydrolytic metalloenzymes and catalyze the hydrolysis of activated carboxylate esters.^28,38,39^ Notably, in our work, the opposite outcome is achieved as compared to the previous art; namely, we demonstrate that a metal-bound aminoalkoxide couples to an activated carboxylic acid derivative (i.e., adenylate or thioester) to form a stable ester product. Also, it appears that simple copper salts can carry out such transformations, and specially designed ligands to control the coordination environment are not required.

### Self-Assembly and Molecular Properties of Sphingomyelin Isomers

**Figure 3:**
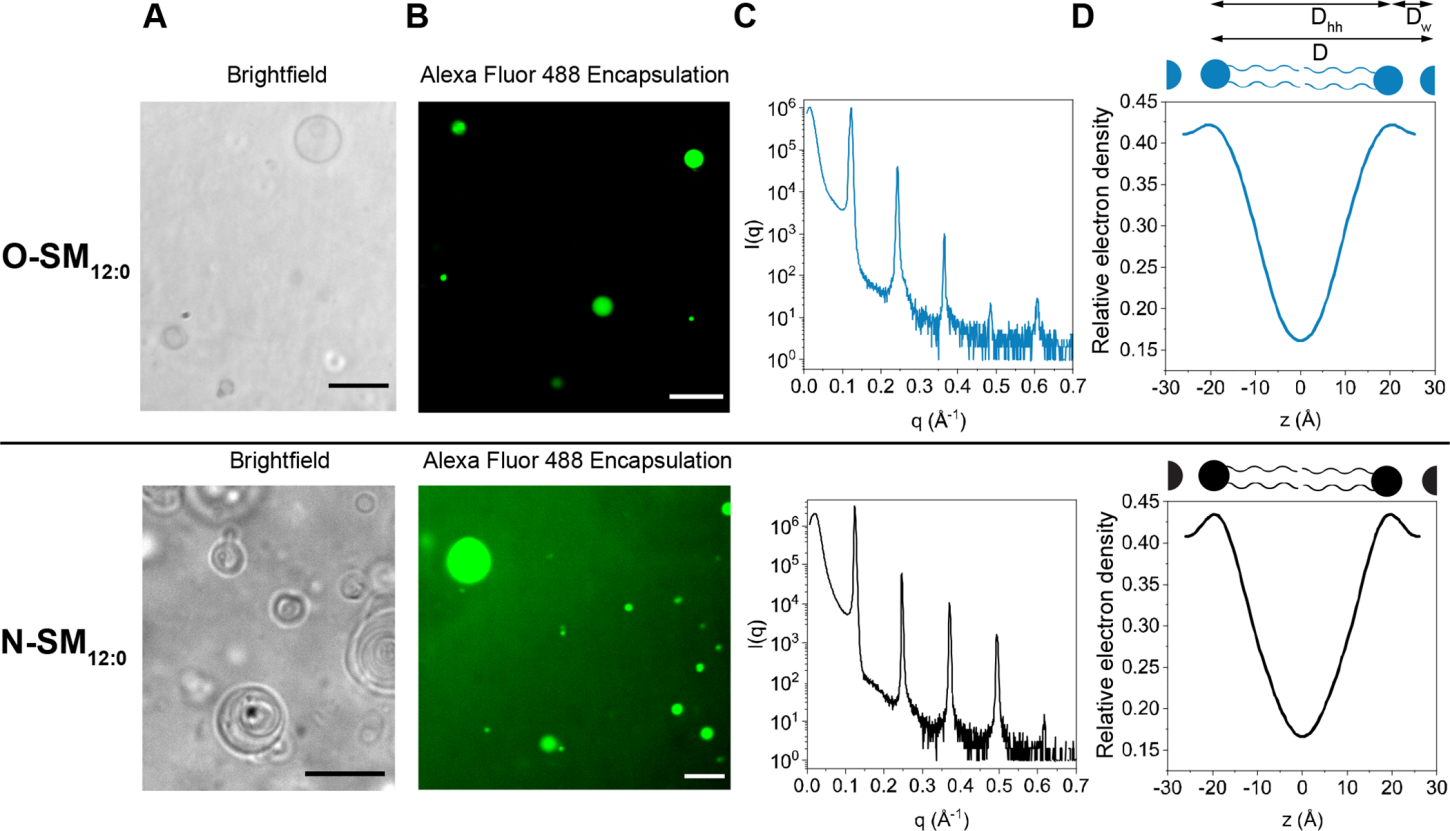
Characterization of the self-assembly behavior of the sphingomyelin isomers **O-SM**_**12:0**_ (top panel) and **N-SM**_**12:0**_ (bottom panel). (A) Bright-field microscopy images show giant vesicles formed upon hydration of a lipid film. (B) In a separate preparation, the water-soluble dye Alexa Fluor 488 was encapsulated inside vesicles and imaged by fluorescence microscopy. All scale bars denote 10 μm. (C) XRD intensity profiles from oriented lipid multilayers on a solid substrate. (D) Electron density profiles (EDPs) were calculated from the intensity profiles. 0's on the *x* axes represent the bilayer midplane.

Sphingomyelins are an important class of eukaryotic membrane lipids and a major component of cellular membranes. Functionalization of the sphingoid base backbone engenders a large family of sphingolipids that play crucial roles in membrane biology and provide many biologically active metabolites. Lipids with two alkyl chains, like diacyl phospholipids and N-acyl sphingolipids, are well-known to form bilayer vesicles and make up the majority of living cellular membranes. Thus, Cu(II)-directed O-acylation of 1,2-amino alcohol amphiphiles has the potential to drive the formation of lipid vesicles or protocells in the absence of enzymes. We hypothesized that O-acylated 1,2-amino alcohol lipids, like **O-SM**_**12:0**_, would self-assemble to form bilayers because isomeric native (N-acylated) sphingomyelins having phosphocholine headgroups are known to self-assemble into vesicles in aqueous media.^40−42^ In the metal ion-catalyzed synthesis reactions forming O- or N-acylated sphingomyelins, we observed the *in situ* formation of membrane-bound vesicles by microscopy (Figure S9). Because O-acylated sphingomyelin analogues have not previously been reported, we sought to characterize the physical properties of the membranes formed from pure **O-SM**_**12:0**_ and compared them with the membranes formed from **N-SM**_**12:0**_. Upon gentle hydration of a thin dried lipid film in water or buffered solutions, both lipids formed membrane bound vesicles, as observed by phase contrast microscopy (Figure 3A, Figure S10). The vesicles also were able to encapsulate water-soluble dyes, such as Alexa Fluor 488 (Figure 3B, Figure S10).

**Figure 4:**
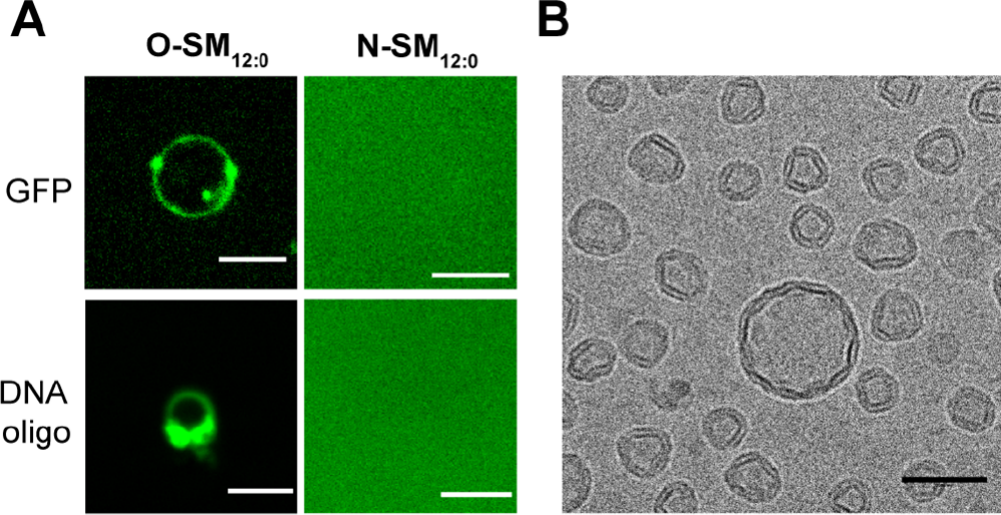
(A) Spinning disk confocal microscopy showing the interaction of either **O-SM**_**12:0**_ (left column) or **N-SM**_**12:0**_ (right column) with sfGFP and 5′-FAM-labeled DNA oligonucleotide, respectively. Scale bars represent 5 μm. (B) Cryogenic electron microscopy images of vesicles formed by the hydration of a film of **O-SM**_**16:0**_ with water. Scale bar: 50 nm.

Having evidence of membrane formation, we next measured the structural parameters of the formed membranes. X-ray diffraction (XRD) methods have been widely applied to lamellar lipid multilayers and are well-established to quantitatively study lipid bilayer structures.^43,44^ We prepared multilayered oriented films from **O-SM**_**12:0**_ and **N-SM**_**12:0**_ on silicon wafers and carried out XRD experiments. We obtained five Bragg peaks at equal *q*-spacing corresponding to a lamellar phase (Figure 3C). We calculated the electron density profiles (EDPs) of **O-SM**_**12:0**_ and **N-SM**_**12:0**_ bilayers (Figure 3D) from which we obtained the distance between lipid headgroups (*D*_hh_) and the thickness of the water layer between lipid bilayers (*D*_w_). We obtained a lamellar repeat distance (*d*-spacing) of 51.6 Å and a membrane thickness of 39.8 Å for **O-SM**_**12:0**_ multilayers. For **N-SM**_**12:0**_ multilayers, we obtained a slightly lower *d*-spacing value of 51.0 Å and a membrane thickness of 39.2 Å (Figure 3D). For both samples, the water thickness between lipid bilayers was established as 11.8 Å, suggesting a similar headgroup hydration (Figure 3D). Although the X-ray studies suggest that **O-SM**_**12:0**_ and **N-SM**_**12:0**_ present similar structural parameters when organized as a stack of lipid multilayers, the vesicles formed from the lipids showed marked differences. For instance, we found that unlike membranes formed from **N-SM**_**12:0**_ or glycerophospholipids, the **O-SM**_**12:0**_ membranes could not be visualized by fluorescence microscopy by staining with lipophilic dyes such as Nile Red (Figure S10) and Texas Red DHPE. A possible explanation is that the presence of the free −NH_2_(C2) group at the hydrophobic–hydrophilic interface of **O-SM**_**12:0**_ quenches the fluorescence of the lipophilic dyes. On the other hand, we speculated that the presence of a free interfacial ionizable −NH_2_(C2) group could facilitate the binding of negatively charged substrates. To test this, we incubated vesicles composed of **O-SM**_**12:0**_ membranes with negatively charged macromolecules such as green fluorescent protein (sfGFP) and a fluorescently labeled DNA oligonucleotide (5′-FAM dN_20_). When membranes were observed by microscopy (Figure 4A, Figure S10), there were clear staining and accumulation of substrates at the membrane boundary. In contrast, we did not observe any binding of sfGFP or a fluorescent oligonucleotide with **N-SM**_**12:0**_ vesicles (Figure 4A, Figure S10). These results suggest that O-acylated 1,2-amino alcohol lipids can act as scaffolds for binding negatively charged molecules.

We also assayed the temperature-dependent fluidity of the membranes based on the fluorescence anisotropy of the dye 1,6-diphenyl-1,3,5-hexatriene (DPH). When a lipid undergoes a transition from gel to fluid phase, an abrupt change in fluorescence anisotropy of DPH is observed.^45^ In the case of **O-SM**_**12:0**_, no abrupt change in anisotropy was observed over the temperature range of 7–79 °C, suggesting that the membranes are fluid over this range (Figure S11). In comparison, **N**-**SM**_**12:0**_ showed a significant change in anisotropy centered at about 20.3 °C (Figure S11), which is in good agreement with the reported gel-to-fluid phase transition temperature (*T*_m_) of 23.4 °C obtained from differential scanning calorimetry (DSC) measurements.^32^ We further measured the phase transition temperatures of **O-SM**_**16:0**_ and obtained a *T*_m_ value of 30.9 °C (Figure S11). This data suggested that **O**-**SM**_**16:0**_ formed gel phase membranes at room temperature. Indeed, using cryogenic electron microscopy, we found that the **O**-**SM**_**16:0**_ vesicles displayed membrane edges and nonspherical morphologies, which are characteristic of gel phase lipids (Figure 4B).^46^ In the case of **N**-**SM**_**16:0**_, we obtained a value of 40.7 °C (Figure S11) for *T*_m_, which is in good agreement with the previously reported value.^47^ We reason that the O-acylated sphingomyelin analogues lack the intermolecular hydrogen bonding between amide groups when compared to their N-acylated counterparts^48^ and therefore display lower gel-to-fluid phase transition temperatures.

### Broader Scope of Cu(II)-Directed O-Acylation of 1,2-Amino Alcohol Amphiphiles

Although sphingosylphosphorylcholine can be considered a prototypical 1,2-amino alcohol amphiphile, we were interested in understanding how general this approach was to the esterification of other similar amphiphilic species. Sphingosine (**8**) is a biologically occurring amphiphile in which there are two nonequivalent −OH groups vicinal to an −NH_2_ group. When we carried out the Cu(II)-directed acylation of **8** using **5**, we found that **8** primarily underwent O-acylation, albeit at both −OH groups (**8.1** or **8.2,**Figure S12). A minor fraction of the N-acylated product (**8.3**) was obtained as well (Figure S12). We assigned the O-acylation products based on the characteristic HPLC retention times and mass spectral ionization patterns.

**Scheme 1:**
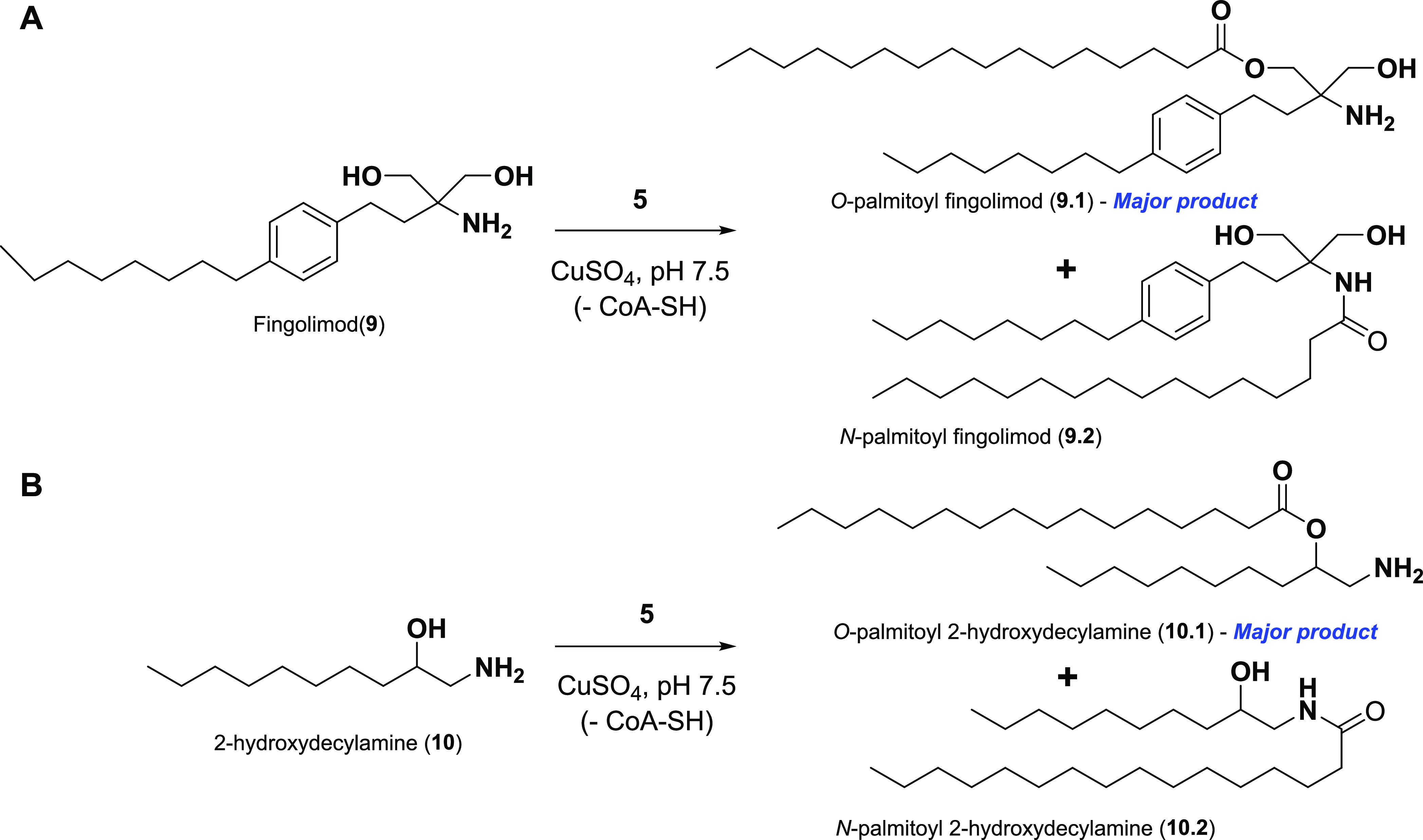
Metal Ion-Directed Selective O-Acylation of Nonbiological 1,2-Amino Alcohol Amphiphiles (A) Fingolimod (**9**). (B) 2-Hydroxydecylamine (**10**).

There are several examples of bioactive molecules containing 1,2-amino alcohols that mimic the structure of sphingolipids and act as inhibitors. For instance, fingolimod (**9**) is a sphingosine analog and FDA-approved immunomodulatory agent used for the treatment of multiple sclerosis.^49^ In fingolimod, there are two equivalent primary −OH groups vicinal to the −NH_2_ group. We found that Cu(II) could direct the acylation of one of the hydroxyl groups of **9** using **5** (Scheme 1A, Figure S13). This finding was intriguing, as both −OH groups are equally reactive, yet our Cu(II)-directed approach yielded only the mono O-acylation product (**9.1**). We confirmed the formation of O-acylated fingolimod by subjecting the products to selective hydrolysis using 1 M NaOH. Only **9.1** underwent hydrolysis to palmitic acid and **9**, but the N-acylated product (**9.2**) was unhydrolyzed.

Previous work has explored the prebiotic relevance of 1,2-amino alcohol and its possible role as early lipid precursors. For example, Mullen and Sutherland introduced 1,2-amino alcohols like 2-hydroxydecylamine (**10**) as prebiotic cationic amphiphiles.^50^ Such species may have been prebiotically synthesized by the reaction of hydrogen cyanide and alkyl aldehydes, the latter being established as products of Fischer–Tropsch reactions, followed by the reduction of the resulting cyanohydrins (Figure S14A). Although such single-chain 1,2-amino alcohols are very interesting as potential prebiotic amphiphiles, it is well established that two-chain lipids typically form membrane assemblies at concentrations much lower than those of single-chain lipids. Two-chain cationic lipids might have allowed more stable binding of RNA to protocell membranes in a putative RNA World.^51^ We were thus interested in determining if Cu(II) ions could catalyze the O-acylation of **10** to generate a two-chain ester with a cationic headgroup (Scheme 1B). Using HPLC–ELSD, we determined that the use of 1 equiv of **5** as an acylating agent exclusively afforded the O-acylation product (**10.1**), as indicated by the early retention time of 3.7 min compared to the N-acylation product (**10.2**, Scheme S6) that has a retention time of 4.7 min (Figure S14B).

## Conclusions

In summary, we have found that metal ions can direct the selective O-acylation of 1,2-amino alcohol amphiphiles in aqueous media. We show that this reaction leads to the formation of O-acylated analogues of sphingomyelin and other sphingolipid species that have previously been unreported (see Supplementary Note). In the past, speculations have been made about whether such species can exist in cells transiently and have any role in sphingolipid homeostasis.^52^ Given that our method of generating O-acylated sphingomyelin relies on biologically relevant amphiphiles, acylating agents, and metal ions, it may be worth exploring if similar reactions take place in cells, especially under abnormal conditions arising from the accumulation of excess metals, such as Wilson’s disease that results in an excess of copper in tissues.^53^ Membrane properties of sphingomyelins are largely influenced by intra- and intermolecular networks formed by interfacial hydrogen bonding, and small structural differences can have a large impact on these interactions.^42^ Membranes formed from O-acylated sphingomyelins showed marked differences in structure and fluidity compared to membranes formed by the N-acylated isomers. O-acylated sphingomyelins are thus expected to have different hydrogen bonding interactions with native lipids, and a more thorough biophysical investigation will be required to understand what kind of interactions may occur within cellular membranes and how this impacts membrane structure. Our method of synthesis of sphingomyelin analogues involved selective O-acylation of a hydroxy group in the presence of an adjacent free amine. Although chemoselective acylation of alcohols has been demonstrated in the presence of amines in organic solvents under both metal catalysis and organocatalysis,^8,9,36,54−56^ such transformations are generally regarded as challenging, particularly in aqueous media.^36,57^ Therefore, our method may facilitate the development of mild strategies for selective O-acylation of organic molecules, which is of interest in medicinal chemistry and carbohydrate chemistry.^10,58−60^ Another area where we foresee applications of our methodology is the development of artificial metalloenzymes capable of catalyzing acylation reactions.^61^ Finally, exploring metal ion-catalyzed selective high-yielding acylation reactions in aqueous media is important in prebiotic chemistry, particularly in the context of the synthesis of phospholipids and other complex membrane lipids.^5,62^ Our method of metal ion-directed acylation of 1,2-amino alcohol amphiphiles leads to stable and high-yielding ester formation particularly at slightly acidic pH. Such transformations may have been plausible in the acidic realm of the Hadean Ocean.^51^ Of particular interest is the acylation of glycerol, the core group of all modern phospholipids. Nearly five decades ago, the laboratory of David Deamer showed that acylation of glycerol with fatty acids can be carried out through a combination of heat, evaporation, minerals, and prebiotic condensing agents.^63^ Our findings with metal-ion directed acylation of 1,2-amino alcohols raise the question of whether simple vicinal diols like glycerol may be acylated under mild aqueous conditions as well. Indeed, our preliminary results show that Cu(II) can direct monoacylation of glycerol with fatty acyl adenylates (Figure S15). Although we could only detect trace quantities of the product, this reaction is not possible in the absence of catalysis. Optimization of the ligands and metal coordination environment may lead to higher yielding outcomes, and such efforts are currently being explored in our laboratory.

## Supporting Information Available

Supplementary Schemes S1–S7, Synthetic Procedures, Supplementary Note, Supplementary Figures S1–S15, and NMR spectral data (PDF)
