# Supplementary material for: Chemoselective Esterification of Natural and Prebiotic 1,2-Amino Alcohol Amphiphiles in Water
Source: J Am Chem Soc. 2023 Dec 1;145(49):27149–59. doi: 10.1021/jacs.3c12038 (PMC10722506; doi:10.1021/jacs.3c12038)
Supplement: Supplementary file 1 — ja3c12038_si_001.pdf [file ja3c12038_si_001.pdf]

# Supporting Information

# Chemoselective Esterification of Natural and Prebiotic 1,2-amino Alcohol Amphiphiles in Water

Ahanjit Bhattacharya,[1, ⁑, ‡] Lalita Tanwar,[1, ‡] Alessandro Fracassi,[1] Roberto J. Brea,[2] Marta Salvador-Castell,[3] Satyam Khanal,[1] Sunil K. Sinha,[3] and Neal K. Devaraj[1,*]

[1]Department of Chemistry and Biochemistry, University of California, San Diego, 9500 Gilman Drive, Natural Sciences Building, California 92093, United States

[2]Biomimetic Membrane Chemistry (BioMemChem) Group, Centro de Investigacións Científicas Avanzadas (CICA), Universidade da Coruña, Rúa As Carballeiras, 15701, A Coruña, Spain

[3]Department of Physics, University of California, San Diego, 9500 Gilman Drive, Mayer Hall, California 92093, United States

[⁑]*Current address*: Keck Science Building, Department of Chemistry, Stanford University, Stanford, California 94305, United States

[‡]These authors contributed to this work equally

*Correspondence to: ndevaraj@ucsd.edu

Synthetic procedures……………………………………………………………………………..S2

Supplementary Note……………………………………………………………………………..S10

Supplementary Figures…………………………………………………………………………S11

Supplementary References……………………………………………………………………..S27

NMR Spectra………………………………………………………………………………......S28

# Synthetic procedures

**Dodecanoyl-AMP (2)**. **2** was synthesized according to a previously published procedure.[1]

**$^1$H NMR** ($d_6$-DMSO, 500.13 MHz, δ): 8.55 (s, 1H, 1×$CH_{Ar}$), 8.30 (s, 1H, 1×$CH_{Ar}$), 5.94 (d, *J* = 5.6 Hz, 1H, 1×CH), 4.57 (t, *J* = 5.3 Hz, 1H, 1×CH), 4.24-3.98 (m, 4H, 2×CH + 1×$CH_2$), 2.33 (t, *J* = 7.3 Hz, 2H, 1×$CH_2$), 1.55-1.35 (m, 2H, 1×$CH_2$), 1.31-1.09 (m, 16H, 8×$CH_2$), 0.88 (t, *J* = 6.8 Hz, 3H, 1×$CH_3$).

**$^{13}$C NMR** ($d_6$-DMSO, 125.77 MHz, δ): 163.3, 152.8, 148.8, 148.5, 140.8, 118.7, 87.3, 83.6, 73.8, 70.4, 66.0, 34.6, 31.3, 29.0, 29.0, 28.9, 28.7, 28.7, 28.3, 24.1, 22.1, 14.0. MS (ESI-TOF) [m/z (%)]: 530 ($[MH]^+$, 100).

**HRMS (ESI-TOF)** calculated for $C_{22}H_{37}N_5O_8P$ ($[MH]^+$) 530.2380, found 530.2374.

**Oleoyl-AMP (3). 3** was synthesized according to a previously published procedure.[1]

**$^1$H NMR** ($d_6$-DMSO, 500.13 MHz, δ): 8.57 (s, 1H, 1×$CH_{Ar}$), 8.31 (s, 1H, 1×$CH_{Ar}$), 5.93 (d, *J* = 5.8 Hz, 1H, 1×CH), 5.35-5.25 (m, 2H, 2×CH), 4.58 (t, *J* = 5.3 Hz, 1H, 1×CH), 4.22-4.15 (m, 1H, 1×CH), 4.14-4.01 (m, 3H, 1×CH + 1×$CH_2$), 2.33 (t, *J* = 7.3 Hz, 2H, 1×$CH_2$), 2.03-1.87 (m, 4H, 2×$CH_2$), 1.51-1.40 (m, 2H, 1×$CH_2$), 1.33-1.15 (m, 20H, 10×$CH_2$), 0.84 (t, *J* = 6.9 Hz, 3H, 1×$CH_3$).

**$^{13}$C NMR** ($d_6$-DMSO, 125.77 MHz, δ): 169.5, 169.4, 152.9, 148.9, 140.9, 129.7, 129.7, 118.7, 87.3, 83.7, 73.9, 70.6, 66.1, 34.7, 31.4, 29.2, 29.1, 28.9, 28.8, 28.7, 28.7, 28.6, 28.4, 26.7, 26.6, 24.2, 22.2, 14.0.

**HRMS (ESI-TOF)** calculated for $C_{28}H_{46}N_5O_8P$ ($[M-H]^-$) 610.3011, found 610.3009.

***N*-Boc-D-*erythro*-sphingosine phosphocholine (1.1)**. (Scheme S1) To a solution of D-*erythro*-sphingosylphosphorylcholine (**1**, Lyso d18:1 SM-$NH_2$, 4.0 mg, 8.6 µmol) in 250 µL of $H_2O$/dioxane (1:1) was successively added $Boc_2O$ (2.8 mg, 12.9 µmol) and $Et_3N$ (3.6 µL, 25.8 µmol). After 3 h stirring at rt, the solvent was removed under reduced pressure. Then, the corresponding residue was diluted in MeOH (250 µL) and filtered using a 0.2 µm syringe-driven filter. The crude solution was purified by HPLC, affording 4.2 mg of the lysosphingolipid **1.1** as a colorless film [87%, $R_t$ = 7.5 min (Zorbax SB-C18 semipreparative column, 5% *Phase A* in *Phase B*, 15.5 min)].

**$^1$H NMR** ($CDCl_3$, 500.13 MHz, δ): 5.81-5-57 (m, 1H, 1×CH), 5.53-5.38 (m, 1H, 1×CH), 4.59-3.52 (m, 8H, 2×CH + 3×$CH_2$), 3.31 (s, 9H, 3×$CH_3$), 2.10-1.84 (m, 2H, 1×$CH_2$), 1.39 (s, 9H, 3×$CH_3$), 1.34-1.17 (m, 22H, 11×$CH_2$), 0.88 (t, *J* = 6.9 Hz, 3H, 1×$CH_3$).

**$^{13}$C NMR** ($CDCl_3$, 125.77 MHz, δ): 156.0, 134.0, 129.6, 79.2, 71.8, 66.5, 65.5, 59.6, 55.7, 54.6, 32.7, 32.1, 29.9, 29.9, 29.9, 29.9, 29.8, 29.8, 29.7, 29.5, 29.5, 28.7, 22.8, 14.3.

**HRMS (ESI-TOF)** calculated for $C_{28}H_{58}N_2O_7P$ ($[M+H]^+$) 565.3976, found 565.3973.

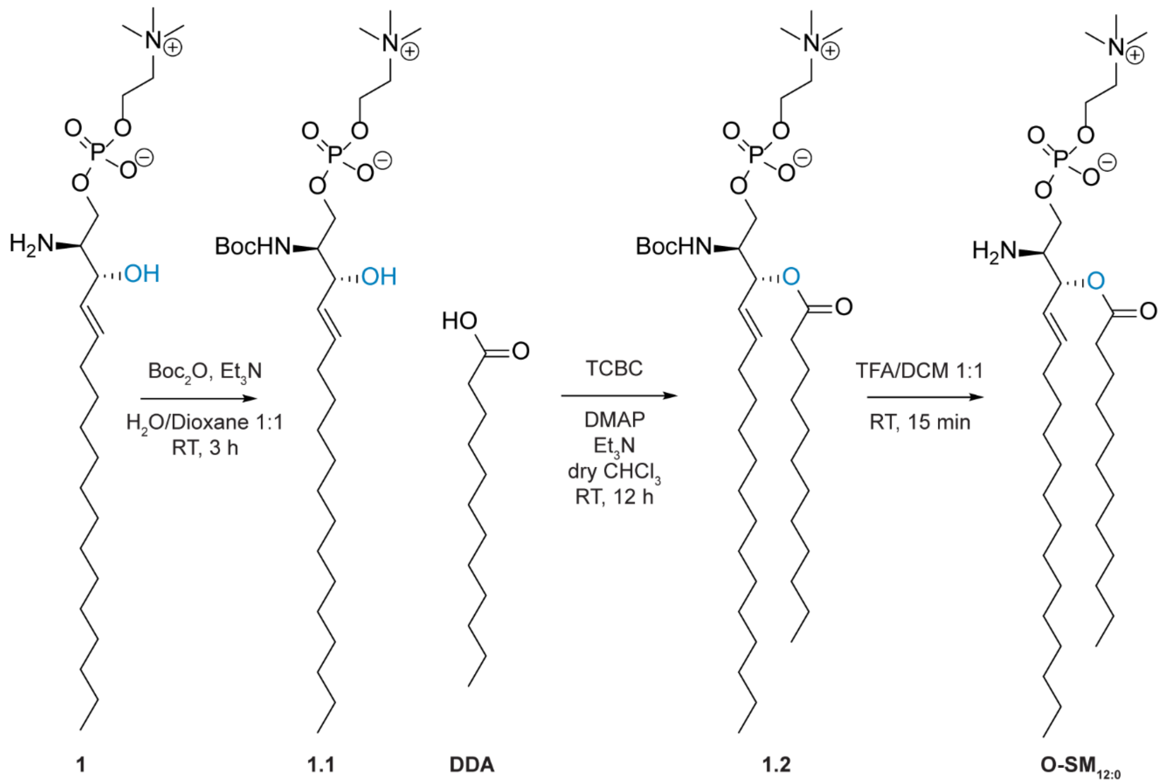


**Scheme S1.** Reaction scheme for synthesizing *O*-dodecanoyl-D-sphingosylphosphocholine (**O-SM$_{12:0}$**).

***N*-Boc-*O*-dodecanoyl-D-*erythro*-sphingosylphosphorylcholine (1.2)**. (Scheme S1) A solution of *N*-Boc-D-*erythro*-sphingosylphosphorylcholine (**1.1**, 4.0 mg, 7.1 µmol), dodecanoic acid (**DDA**, 3.6 mg, 17.7 µmol), DMAP (5.2 mg, 42.5 µmol) and $Et_3N$ (3.5 µL) in dry $CHCl_3$ (275 µL) was stirred for 10 min at rt. Then, 2,4,6-trichlorobenzoyl chloride (TCBC, 7.2 µL) was added, and the reaction was stirred for 12 h at rt. Afterward, $H_2O$ (7.5 µL) was added to the reaction mixture to quench the acid chloride, and the solvent was removed by rotary evaporation to give a yellow oil. Then, the corresponding residue was diluted in MeOH (250 µL), filtered using a 0.2 µm syringe-driven filter. The crude solution was purified by HPLC, affording 4.6 mg of the sphingophingolipid **1.2** as a colorless film [87%, $R_t$ = 10.1 min (Zorbax SB-C18 semipreparative column, *Phase B*, 23.5 min)].

**$^1$H NMR** ($CD_3OD$, 500.13 MHz, δ): 6.89 (d, *J* = 9.4 Hz, 1H, 1×NH), 5.80 (dt, *J1* = 15.3 Hz, *J2* = 6.8 Hz, 1H, 1×CH), 5.45 (ddt, *J1* = 15.3 Hz, *J2* = 7.9 Hz, *J3* = 1.5 Hz, 1H, 1×CH), 5.37-5.25 (m, 1H, 1×CH), 4.35-4.18 (m, 2H, 1×$CH_2$), 4.01-3.94 (m, 1H, 1×CH), 3.93-3.82 (m, 2H, 1×$CH_2$), 3.70-3.58 (m, 2H, 1×$CH_2$), 3.23 (s, 9H, 3×$CH_3$), 2.32 (t, *J* = 7.4 Hz, 2H, 1×$CH_2$), 2.05 (ddt, *J1* = 7.2 Hz, *J2* = 3.6 Hz, *J3* = 2.0 Hz, 2H, 1×$CH_2$), 1.69-1.54 (m, 2H, 1×$CH_2$), 1.43 (s, 9H, 3×$CH_3$), 1.36-1.18 (m, 38H, 19×$CH_2$), 0.90 (t, *J* = 7.0 Hz, 6H, 2×$CH_3$).

**$^{13}$C NMR** ($CD_3OD$, 125.77 MHz, δ): 174.4, 158.1, 138.4, 125.8, 80.2, 75.1, 67.4, 65.4, 60.5, 54.6, 54.3, 35.4, 33.4, 33.1, 30.9, 30.8, 30.8, 30.8, 30.7, 30.7, 30.6, 30.5, 30.5, 30.3, 30.2, 30.0, 28.8, 26.1, 23.8, 14.5.

**HRMS (ESI-TOF)** calculated for $C_{40}H_{80}N_2O_8P$ ($[M+H]^+$) 747.5647, found 747.5649.

***O*-dodecanoyl-D-*erythro*-sphingosylphosphorylcholine (O-SM$_{12:0}$)**. (Scheme S1) A solution of *N*-Boc-*O*-dodecanoyl-D-*erythro*-sphingosylphosphorylcholine (**1.2**, 4.0 mg, 5.4 μmol) in 500 μL of TFA/$CH_2Cl_2$ (1:1) was stirred at rt for 15 min. After the removal of the solvent, the residue was dried under a high vacuum for 3 h. Then, the corresponding residue was diluted in MeOH (500 μL), filtered using a 0.2 μm syringe-driven filter, and the crude solution was purified by HPLC, affording 2.7 mg of **O-SM$_{12:0}$** as a colorless oil [77%, $t_R$ = 4.9 min (Zorbax SB-C18 semipreparative column, *Phase B*, 23.5 min)].

**$^1$H NMR** ($CD_3OD$, 500.13 MHz, δ): 6.02-5.82 (m, 1H, 1×CH), 5.53-5.39 (m, 2H, 2×CH), 4.36-4.24 (m, 2H, 1×$CH_2$), 4.12 (ddd, *J1* = 11.5 Hz, *J2* = 6.1 Hz, *J3* = 3.6 Hz, 1H, 0.5×$CH_2$), 3.96 (dt, *J1* = 11.4 Hz, *J2* = 7.1 Hz, 1H, 0.5×$CH_2$), 3.65 (dd, *J1* = 6.0 Hz, *J2* = 3.4 Hz, 2H, 1×$CH_2$), 3.59-3.53 (m, 1H, 1×CH), 3.23 (s, 9H, 3×$CH_3$), 2.40 (t, *J* = 7.4 Hz, 2H, 1×$CH_2$), 2.18-2.01 (m, 2H, 1×$CH_2$), 1.68-1.56 (m, 2H, 1×$CH_2$), 1.47-1.26 (m, 38H, 19×$CH_2$), 0.90 (t, *J* = 6.9 Hz, 6H, 2×$CH_3$).

**$^{13}$C NMR** ($CD_3OD$, 125.77 MHz, δ): 172.4, 139.1, 127.2 and 122.4, 71.6, 66.0, 62.5, 59.2, 53.8, 53.2, 33.6, 32.0, 31.7, 29.5, 29.4, 29.4, 29.4, 29.4, 29.3, 29.2, 29.1, 29.1, 29.1, 28.8, 28.8, 28.5, 24.6, 22.4, 13.1.

**HRMS (ESI-TOF)** calculated for $C_{35}H_{72}N_2O_6P$ ($[M+H]^+$) 647.5123, found 647.5108.

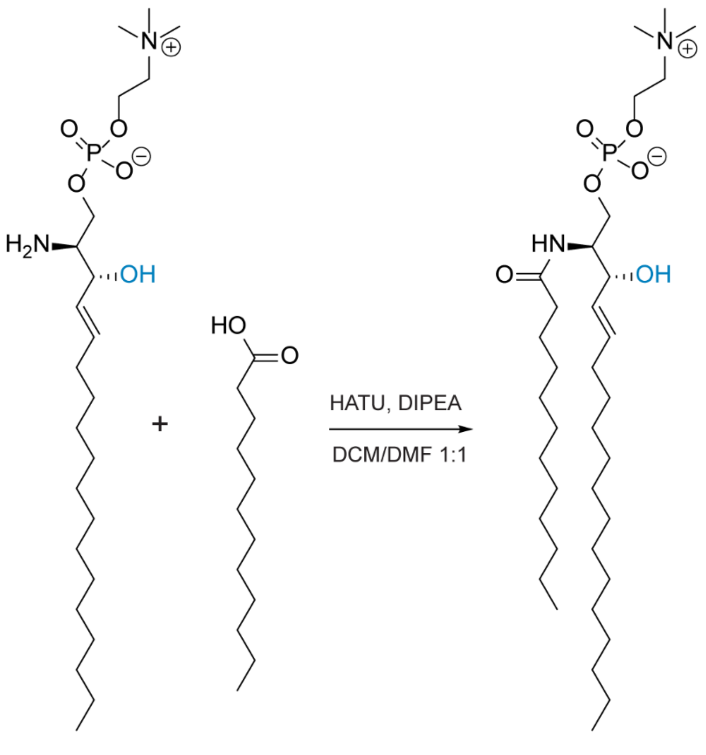


**1** **DDA** **N-SM$_{12:0}$**

**Scheme S2.** Reaction scheme for the synthesis of *N*-dodecanoyl-D-*erythro*-sphingosylphosphorylcholine (**N-SM$_{12:0}$**).

***N*-dodecanoyl-D-*erythro*-sphingosylphosphorylcholine (N-SM$_{12:0}$)**. (Scheme S2) A solution of dodecanoic acid (**DDA**, 1.5 mg, 7.5 μmol) in DCM/DMF (1:1) (250 μL) was stirred at 0 °C for 10 min, and then HATU (3.2 mg, 8.3 μmol) and DIPEA (5.3 μL, 30.1 μmol) were successively added. After 10 min stirring at 0 °C, D-*erythro*-sphingosylphosphorylcholine (**1**, Lyso d18:1 SM-NH$_2$, 3.5

mg, 7.5 µmol) was added. After 1 h stirring at rt, the mixture was concentrated under reduced pressure. The corresponding residue was dissolved in MeOH (500 µL), filtered using a 0.2 µm syringe-driven filter, and the crude solution was purified by HPLC, affording 3.5 mg of **N-SM$_{12:0}$** as a colorless film [72%, $t_R$ = 7.9 min (Zorbax SB-C18 semipreparative column, *Phase B*, 23.5 min)].

**$^1$H NMR** ($CD_3OD$, 500.13 MHz, δ): 7.98 (d, *J* = 9.0 Hz, 1H, 1×NH), 5.70 (dt, *J1* = 15.3 Hz, *J2* = 6.7 Hz, 1H, 1×CH), 5.70 (ddt, *J1* = 15.3 Hz, *J2* = 7.8 Hz, *J3* = 1.5 Hz, 1H, 1×CH), 4.71-4.58 (m, 1H, 1×CH), 4.36-4.21 (m, 2H, 1×$CH_2$), 4.14-3.92 (m, 3H, 1×$CH_2$ + 1×CH), 3.71-3.55 (m, 2H, 1×$CH_2$), 3.22 (s, 9H, 3×$CH_3$), 2.18 (td, *J1* = 7.7 Hz, *J2* = 6.9 Hz, *J3* = 1.9 Hz, 2H, 1×$CH_2$), 2.03 (ddt, *J1* = 12.6 Hz, *J2* = 8.4 Hz, *J3* = 4.0 Hz, 2H, 1×$CH_2$), 1.66-1.46 (m, 2H, 1×$CH_2$), 1.42-1.24 (m, 38H, 19×$CH_2$), 0.90 (t, *J* = 6.9 Hz, 6H, 2×$CH_3$).

**$^{13}$C NMR** ($CD_3OD$, 125.77 MHz, δ): 174.5, 133.8, 129.8, 71.1, 66.0, 64.4, 59.0, 53.8, 53.2, 36.0, 35.9, 32.1, 31.7, 31.7, 29.5, 29.5, 29.4, 29.4, 29.4, 29.4, 29.3, 29.3, 29.2, 29.1, 29.1, 29.1, 29.1, 29.0, 25.8, 22.4, 13.1.

**HRMS (ESI-TOF)** calculated for $C_{35}H_{72}N_2O_6P$ ($[M+H]^+$) 647.5123, found 647.5124.

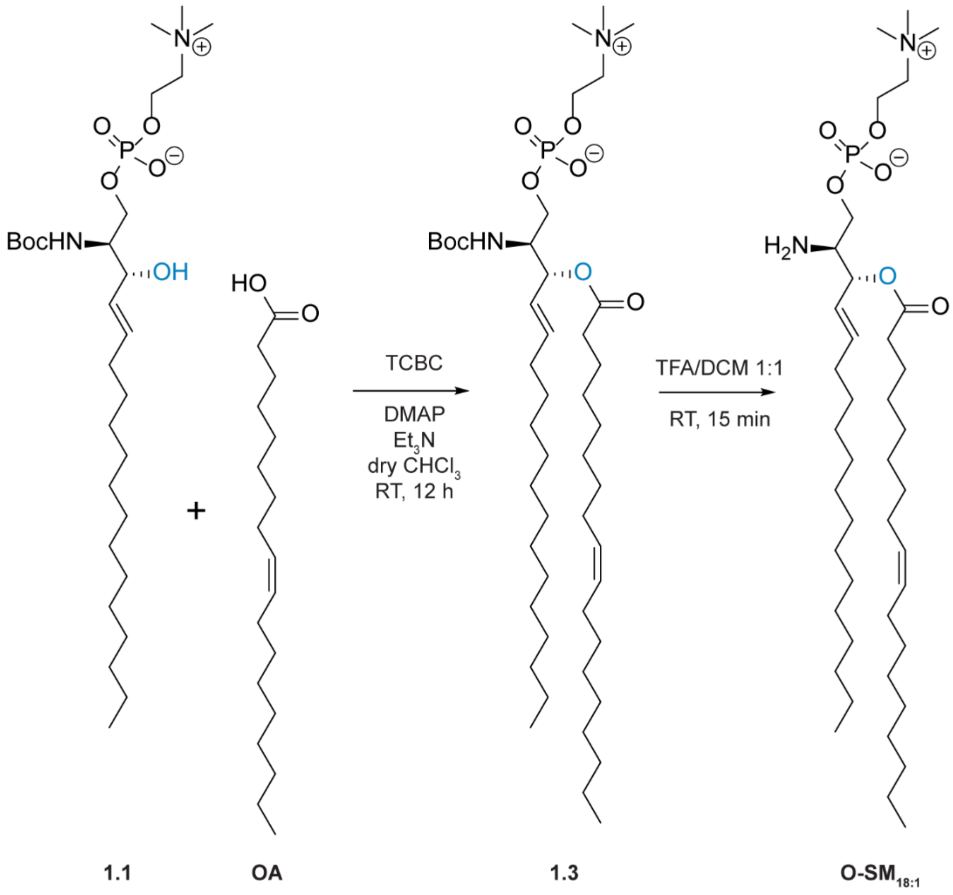


**Scheme S3.** Reaction scheme for the synthesis of *O*-oleoyl-D-*erythro*-sphingosylphosphorylcholine (**O-SM$_{18:1}$**).

***N*-Boc-*O*-oleoyl-D-*erythro*-sphingosylphosphocholine (1.3)**. (Scheme S3) A solution of *N*-Boc-D-erythro-sphingosylphosphorylcholine (**1.1**, 4.0 mg, 7.1 µmol), oleic acid (**OA**, 5.0 mg, 17.7

μmol), DMAP (5.2 mg, 42.5 μmol) and $Et_3N$ (3.5 μL) in dry $CHCl_3$ (275 μL) was stirred for 10 min at rt. Then, 2,4,6-trichlorobenzoyl chloride (TCBC, 7.2 μL) was added and the reaction was stirred for 12 h at rt. Afterwards, $H_2O$ (7.5 μL) was added to the reaction mixture to quench the acid chloride, and the solvent was removed by rotary evaporation to give a yellow oil. Then, the corresponding residue was diluted in MeOH (250 μL), filtered using a 0.2 μm syringe-driven filter, and the crude solution was purified by HPLC, affording 4.9 mg of **1.3** as a colorless film [82%, $R_t$ = 19.4 min (Zorbax SB-C18 semipreparative column, *Phase B*, 28.5 min)].

**$^1$H NMR** ($CDCl_3$, 500.13 MHz, δ): 5.86-5.66 (m, 1H, 1 × CH), 5.51-5.20 (m, 3H, 3×CH), 4.61-3.61 (m, 8H, 2×CH + 3×$CH_2$), 3.36 (s, 9H, 3×$CH_3$), 2.70-2.14 (m, 4H, 2×$CH_2$), 2.12-1.92 (m, 4H, 2×$CH_2$), 1.68-1.50 (m, 2H, 1×$CH_2$), 1.41 (s, 9H, 3×$CH_3$), 1.34-1.16 (m, 42H, 21×$CH_2$), 0.88 (t, $J$ = 6.9 Hz, 6H, 2×$CH_3$).

**$^{13}$C NMR** ($CDCl_3$, 125.77 MHz, δ): 173.1, 163.2,* 155.8, 137.6, 130.2, 129.8, 124.2, 79.5, 73.4, 66.7, 65.8, 64.9, 59.8, 54.7, 34.8, 34.6, 32.5, 32.1, 32.1, 32.0, 29.9, 29.9, 29.9, 29.8, 29.8, 29.7, 29.7, 29.6, 29.5, 29.5, 29.5, 29.4, 29.4, 29.3, 29.1, 27.4, 27.4, 26.9, 26.9, 25.1, 22.8, 14.3. MS (ESI-TOF) [m/z (%)]: 851 ($[M+Na]^+$, 100).

**HRMS (ESI-TOF)** calculated for $C_{46}H_{89}N_2O_8PNa$ ($[M+Na]^+$) 851.6249, found 851.6246.

*Carbon peak corresponding to formic acid

***O*-oleoyl-D-*erythro*-sphingosylphosphorylcholine (O-SM$_{18:1}$)**. (Scheme S3) A solution of *N*-Boc-D-*erythro*-*O*-oleoyl-sphingosylphosphorylcholine (**1.3**, 3.0 mg, 3.6 mol) in 500 μL of TFA/$CH_2Cl_2$ (1:1) was stirred at rt for 15 min. After removal of the solvent, the residue was dried under high vacuum for 3 h. Then, the corresponding residue was diluted in MeOH (500 μL), filtered using a 0.2 μm syringe-driven filter, and the crude solution was purified by HPLC, affording 1.9 mg of the sphingolipid **O-SM$_{18:1}$** as a colorless oil [74%, $t_R$ = 14.3 min (Zorbax SB-C18 semipreparative column, *Phase B*, 28.5 min)].

**$^1$H NMR** ($CDCl_3$, 500.13 MHz, δ): 6.06-5.78 (m, 1H, 1×CH), 5.59-5.43 (m, 1H, 1×CH), 5.44-5.27 (m, 2H, 2×CH), 4.67-3.47 (m, 8H, 2×CH + 3×$CH_2$), 3.26 (s, 9H, 3×$CH_3$), 2.98 (br s, 2H, 1×$NH_2$), 2.48-2.22 (m, 2H, 1×$CH_2$), 2.12-1.95 (m, 4H, 2×$CH_2$), 1.94-1.70 (m, 2H, 1×$CH_2$), 1.68-1.48 (m, 2H, 1×$CH_2$), 1.46-1.11 (m, 42H, 21×$CH_2$), 0.87 (t, J = 6.8 Hz, 6H, 2×$CH_3$).

**$^{13}$C NMR** ($CDCl_3$, 125.77 MHz, δ): 173.1, 160.4,* 140.1, 130.1, 129.8, 124.9, 71.0, 66.5, 65.8, 62.6, 60.1, 54.7, 34.6, 34.6, 34.3, 34.2, 32.4, 32.1, 32.0, 32.0, 29.9, 29.9, 29.9, 29.8, 29.7, 29.6, 29.5, 29.5, 29.4, 29.2, 29.0, 28.7, 27.4, 27.3, 26.9, 26.8, 22.9, 22.8, 14.3, 14.3. MS (ESI-TOF) [m/z (%)]: 729 ($[MH]^+$, 100).

*Carbon peak corresponding to formic acid (Sphingomyelin **O-SM$_{18:1}$** was obtained as a formate salt).

**HRMS (ESI-TOF)** calculated for $C_{41}H_{82}N_2O_6P$ ($[MH]^+$) 729.5905, found 729.5897.

***N*-oleoyl-D-*erythro*-sphingosylphosphorylcholine (N-SM$_{18:1}$)**. Synthesis of **N-SM$_{18:1}$** was carried out in a manner similar to **N-SM$_{12:0}$**.

**$^1$H NMR** ($CDCl_3$, 500.13 MHz, δ): 5.77-5-60 (m, 1H, 1×CH), 5.52-5.39 (m, 1H, 1×CH), 5.38-5.26 (m, 2H, 2×CH), 4.50-4.24 (m, 2H, 1×$CH_2$), 4.25-4.12 (m, 1H, 1×CH), 4.11-4.02 (m, 1H, 1×CH), 3.99-3.89 (m, 2H, 1×$CH_2$), 3.88-3.74 (m, 2H, 1×$CH_2$), 3.33 (s, 9H, 3×$CH_3$), 2.16-1.83 (m, 8H, 4×$CH_2$), 1.65-1.46 (m, 2H, 1×$CH_2$), 1.39-1.18 (m, 42H, 21×$CH_2$), 0.88 (t, $J$ = 7.0 Hz, 6H, 2×$CH_3$).

$^{13}$**C NMR** ($CDCl_3$, 125.77 MHz, δ): 173.7, 134.0, 130.1, 130.1, 129.8, 76.1, 71.5, 66.4, 65.3, 59.5, 54.5, 36.9, 32.8, 32.1, 32.1, 30.1, 30.0, 30.0, 30.0, 29.9, 29.9, 29.9, 29.9, 29.9, 29.8, 29.7, 29.7, 29.7, 29.6, 29.6, 29.5, 29.5, 27.4, 27.4, 26.2, 22.9, 22.8, 14.3. MS (ESI-TOF) [m/z (%)]: 729 ($[MH]^+$, 100).

**HRMS (ESI-TOF)** calculated for $C_{41}H_{82}N_2O_6P$ ($[MH]^+$) 729.5905, found 729.5907.

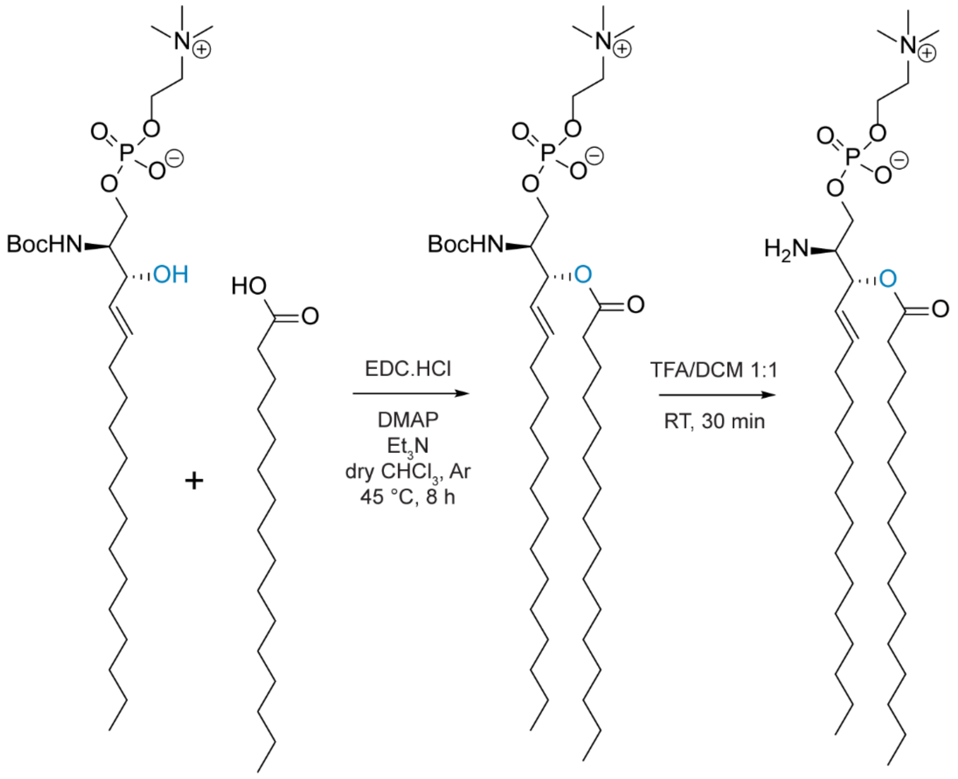


**1.1** **PA** **1.4** **O-SM$_{16:0}$**

**Scheme S4.** Reaction scheme for the synthesis of *O*-palmitoyl-D-*erythro*-sphingosylphosphorylcholine (**O-SM$_{16:0}$**).

***N*-Boc-*O*-palmitoyl-D-*erythro*-sphingosylphosphorylcholine (1.4)**. (Scheme S4) A solution of *N*-Boc-D-*erythro*-sphingosylphosphorylcholine (**1.1**, 7.93 mg, 14.04 µmol), palmitic acid (**PA**, 9.00 mg, 35.1 µmol), EDC.HCl (18.84 mg, 98.29 µmol), DMAP (12.01 mg, 98.29 µmol), and dry $Et_3N$ (7.80 µL, 56.17 µmol) were dissolved in dry $CHCl_3$ (1 mL) was stirred for 45 °C for 8 hours. Afterward the solvent was removed by rotary evaporation to give a pale-yellow solid and directly used in the next step.

***O*-palmitoyl-D-*erythro*-sphingosylphosphorylcholine (O-SM$_{16:0}$)**. (Scheme S4) To the crude *N*-Boc-*O*-palmitoyl-D-*erythro*-sphingosylphosphorylcholine (**1.4**) mixture, 2 mL of TFA/$CH_2Cl_2$ (1:1) was added and the reaction mixture was stirred at rt for 30 min. After the removal of the solvent, the residue was dried under a high vacuum for 3 h. Then, the corresponding residue was diluted in MeOH (1 mL), filtered using a 0.2 µm syringe-driven filter, and the crude solution was purified by HPLC, affording 2.1 mg of **O-SM$_{16:0}$** as a colorless solid [20%, $t_R$ = 11.9 min (Zorbax SB-C18 semipreparative column, *Phase B*, 22 min)].

**$^{1}$H NMR** ($CD_3OD$, 400 MHz, δ): 5.93 (dt, *J* = 13.8, 6.8 Hz, 1H), 5.55-5.39 (m, 2H), 4.29 (s, 2H), 4.12 (ddd, *J* = 10.2, 6.1, 3.7 Hz, 1H), 3.96 (dt, *J* = 11.5, 7.1 Hz, 1H), 3.68-3.61 (m, 2H), 3.23 (s, 9H), 2.40 (t, *J* = 7.3 Hz, 2H), 2.11 (tt, *J* = 7.9, 4.0 Hz, 2H), 1.62 (d, *J* = 9.3 Hz, 2H), 1.29 (s, 49H), 0.92-0.89 (m, 6H).

**$^{13}$C NMR** ($CD_3OD$, 101 MHz, δ): 173.8, 140.5, 123.85, 72.9, 63.8, 60.6, 55.2, 54.6, 35.0, 33.4, 33.1, 30.9, 30.7, 30.6, 30.5, 30.5, 30.3, 30.2, 29.9. 26.0, 23.7, 14.7.

**HRMS (ESI-TOF)** calculated for $C_{39}H_{80}N_2O_6P$ ($[M+H]^+$) 703.5751, found 703.5479.

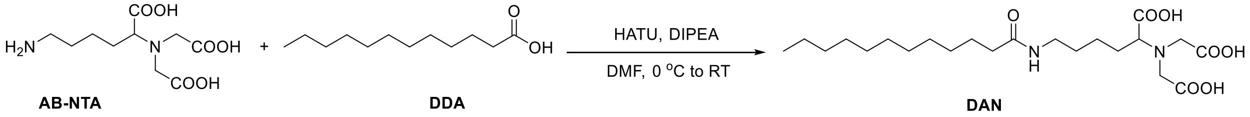


**Scheme S5**. Reaction scheme for the synthesis of Dodecanoyl AB-NTA (**DAN**).

***N*$_α$,*N*$_α$-Bis(carboxymethyl)-L-lysine(dodecanoyl)** or **dodecanoyl AB-NTA (DAN)**. (Scheme S5) A solution of dodecanoic acid (**DDA**, 7.6 mg, 38.1 µmol) in DMF (381 µL) was stirred at 0 °C for 10 min, and then HATU (15.9 mg, 41.9 µmol) and DIPEA (6.6 µL, 41.9 µmol) were successively added. After 10 min stirring at 0 °C, *N*$_α$,*N*$_α$-Bis(carboxymethyl)-*L*-lysine hydrate (**AB-NTA**, 10.0 mg, 38.1 µmol) was added. After 1 h stirring at rt, the mixture was concentrated under reduced pressure. The corresponding residue was dissolved in MeOH (500 µL), filtered using a 0.2 µm syringe-driven filter, and the crude solution was purified by HPLC, affording 13.2 mg of **DAN** as a white solid [78%, $t_R$ = 8.8 min (Zorbax SB-C18 semipreparative column, 50% *Phase A* in *Phase B*, 5 min, and then 5% *Phase A* in *Phase B*, 10 min)].

**$^{1}$H NMR** ($CD_3OD$, 500.13 MHz, δ): 3.78-3.52 (m, 4H, 2×$CH_2$), 3.46 (dd, $J_1$ = 8.4 Hz, $J_1$ = 6.4 Hz, 1H, 1×CH), 3.17 (t, *J* = 6.4 Hz, 2H, 1×$CH_2$), 2.17 (t, *J* = 7.3 Hz, 2H, 1×$CH_2$), 1.87-1.40 (m, 8H, 4×$CH_2$), 1.36-1.21 (m, 16H, 8×$CH_2$), 0.90 (t, *J* = 7.0 Hz, 3H, 1×$CH_3$).

**$^{13}$C NMR** ($CD_3OD$, 125.77 MHz, δ): 176.4, 176.0, 175.9, 66.7, 55.4, 40.0, 37.2, 33.1, 30.8, 30.8, 30.7, 30.7, 30.5, 30.5, 30.3, 30.0, 27.1, 24.8, 23.8, 14.5.

**HRMS (ESI-TOF)** calculated for $C_{22}H_{39}N_2O_7$ ($[M-H]^-$) 443.2763, found 443.2760.

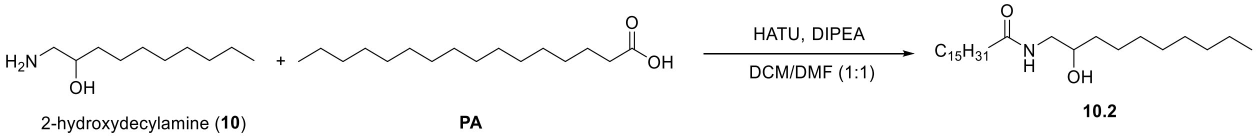


**Scheme S6.** Reaction scheme for the synthesis of *N*-palmitoyl 2-hydroxydecylamine (**10.2**).

***N*-palmitoyl 2-hydroxydecylamine (10.2)**. (Scheme S6) A solution of palmitic acid (**PA**, 2.5 mg, 9.5 µmol) in DCM/DMF (1:1) (250 µL) was stirred at 0 °C for 10 min, and then HATU (2.3 mg, 9.5 µmol) and DIPEA (6.8 µL, 33.1 µmol) were successively added. After 10 min stirring at 0 °C, 2-hydroxydecylamine (**10**, 1.5 mg, 8.7 µmol) was added. After 1 h stirring at rt, the mixture was concentrated under reduced pressure. The corresponding residue was dissolved in MeOH (500 µL), filtered using a 0.2 µm syringe-driven filter, and the crude solution was purified by HPLC,

affording 2.2 mg of **10.2** as a colorless film [63%, $t_R$ = 16.5 min (Zorbax SB-C18 semipreparative column, *Phase B*, 23.5 min)].

**$^1$H NMR** ($CDCl_3$, 500.13 MHz, δ): 8.21-7.99 (m, 1H), 6.05 (s, 1H), 3.72 (dt, *J* = 9.8, 3.7 Hz, 1H), 3.49 (ddd, *J* = 14.1, 6.1, 2.8 Hz, 1H), 3.23-3.05 (m, 1H), 2.32-2.18 (m, 2H), 1.77-1.55 (m, 2H), 1.53-1.36 (m, 2H), 1.26 (dd, *J* = 14.1, 5.9 Hz, 36H), 0.87 (t, *J* = 6.8 Hz, 6H).

**HRMS (ESI-TOF)** calculated for $C_{26}H_{54}NO_2$ ($[M+H]^+$) 412.4149, found 412.4148.

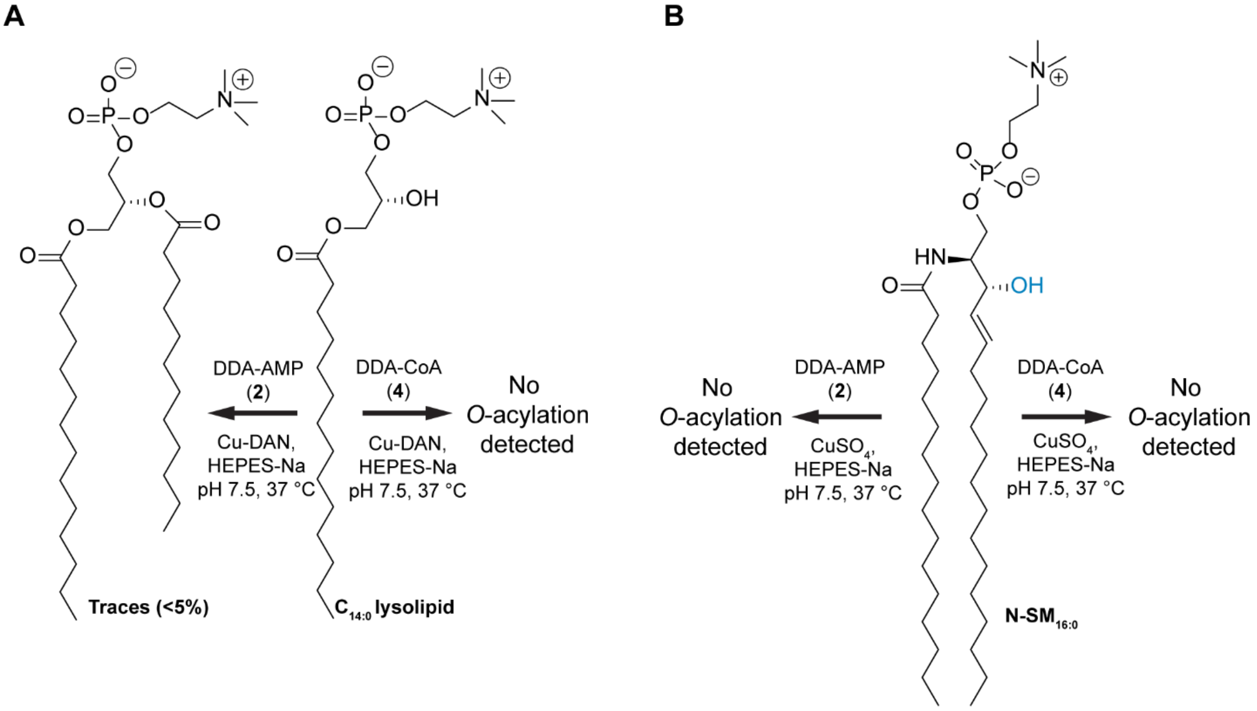


**Scheme S7**. **A**. When $C_{14:0}$ lysolipid is treated with adenylate **2** in the presence of Cu(II), traces of esterified product is detected, but when $C_{14:0}$ lysolipid is treated with thioester **4** in the presence of Cu(II), no esterified product is detected. **B**. When an *N*-acyl sphingomyelin (**N-SM$_{16:0}$**) is treated with an adenylate **2** or thioester **4** in the presence of Cu(II), no *O*-acylation is detected.

## Supplementary note: Diversity of sphingolipid species

Sphingolipids are an important class of membrane lipids occurring universally in eukaryotes and constituting over 1000 variants.[2] They are structural components of biological membranes, such as sphingomyelins in myelin sheaths. Sphingolipids play a crucial role in cellular signal transduction processes and therefore have enormous clinical significance.[3,4] Sphingolipids are also used as an important component of many liposomal drug delivery vehicles and cosmetic products.[5] Structurally, SLs constitute a long-chain of amino alcohol backbone, having a sphingoid base (typically sphingosine) which has two hydroxy (-OH) groups at positions C1 and C3 and an amino (-$NH_2$) group at position C2 (Figure S1). Sphingosine is derivatized in different ways at these positions to generate sphingolipid species with various functions. One of the simplest acylated sphingolipids is ceramide, where the OH(C1) group remains unmodified while the amine is acylated by a fatty acid chain (Figure S1). In rarer cases, modification of OH(C1) of ceramides with a fatty acyl group has also been observed in human and mice epidermis.[6] Transient formation of C1-*O*-acyl and C3-*O*-acyl analogues of ceramides from *N*-acyl ceramides due to *N*→*O* acyl shift has been reported in under strongly acidic conditions or due to prolonged storage of the lipids in chloroform.[7] The OH(C1) group of ceramides may be transformed enzymatically by addition of polar head groups like phosphocholine and phosphoethanolamine to generate sphingomyelins, and with sugar moieties to generate glycosphingolipids (Figure S1).[8] There are a few reports mentioning the occurrence of a three-tailed sphingomyelin in new-born pig plasma and human umbilical cord where the OH(C3) is also acylated with a fatty acid.[9,10] In this work, we describe the stable formation of sphingolipid species where the OH(C3) is acylated, but $NH_2$(C2) is free under biochemically relevant conditions, which to our knowledge have not been reported previously.

# Supplementary figures

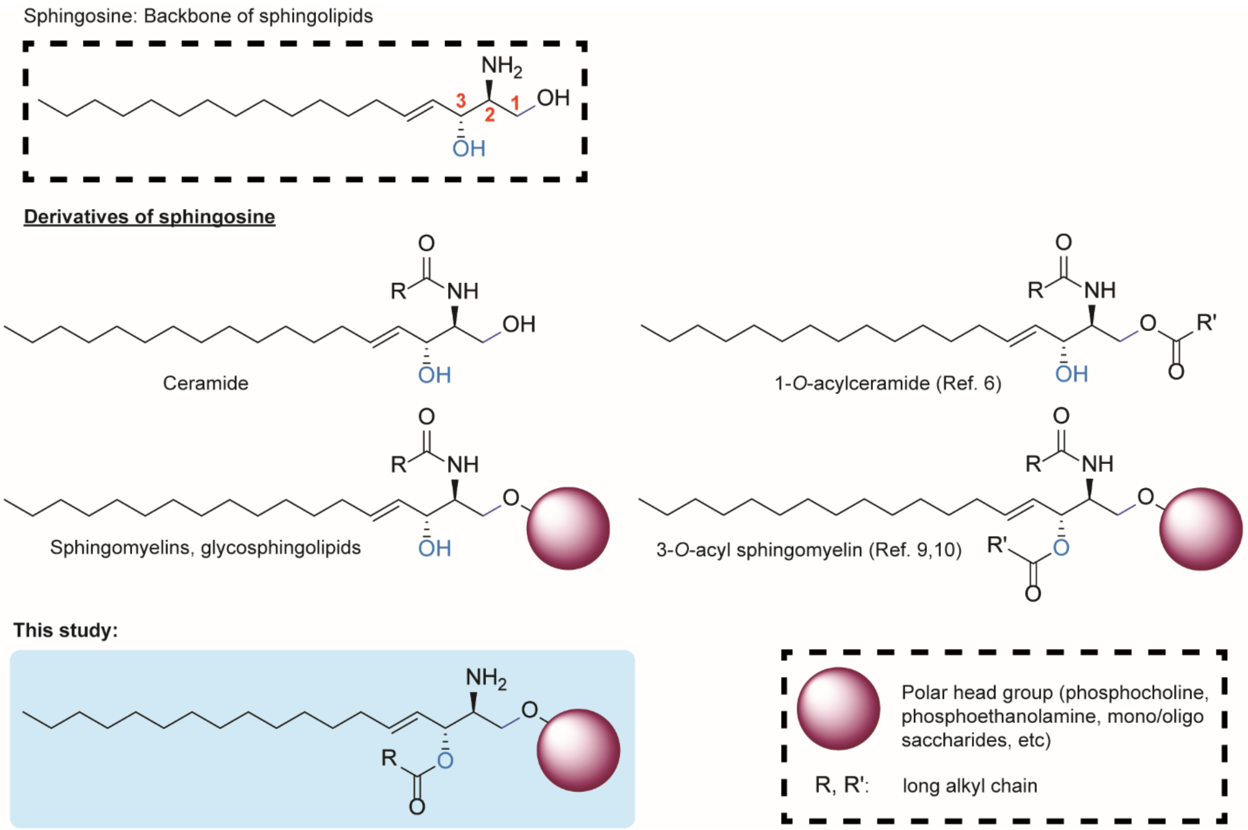


**Figure S1**. General structure of sphingosine molecule and its various derivatives that have been reported. Carbons 1, 2, 3 on sphingosine are indicated in **red**.

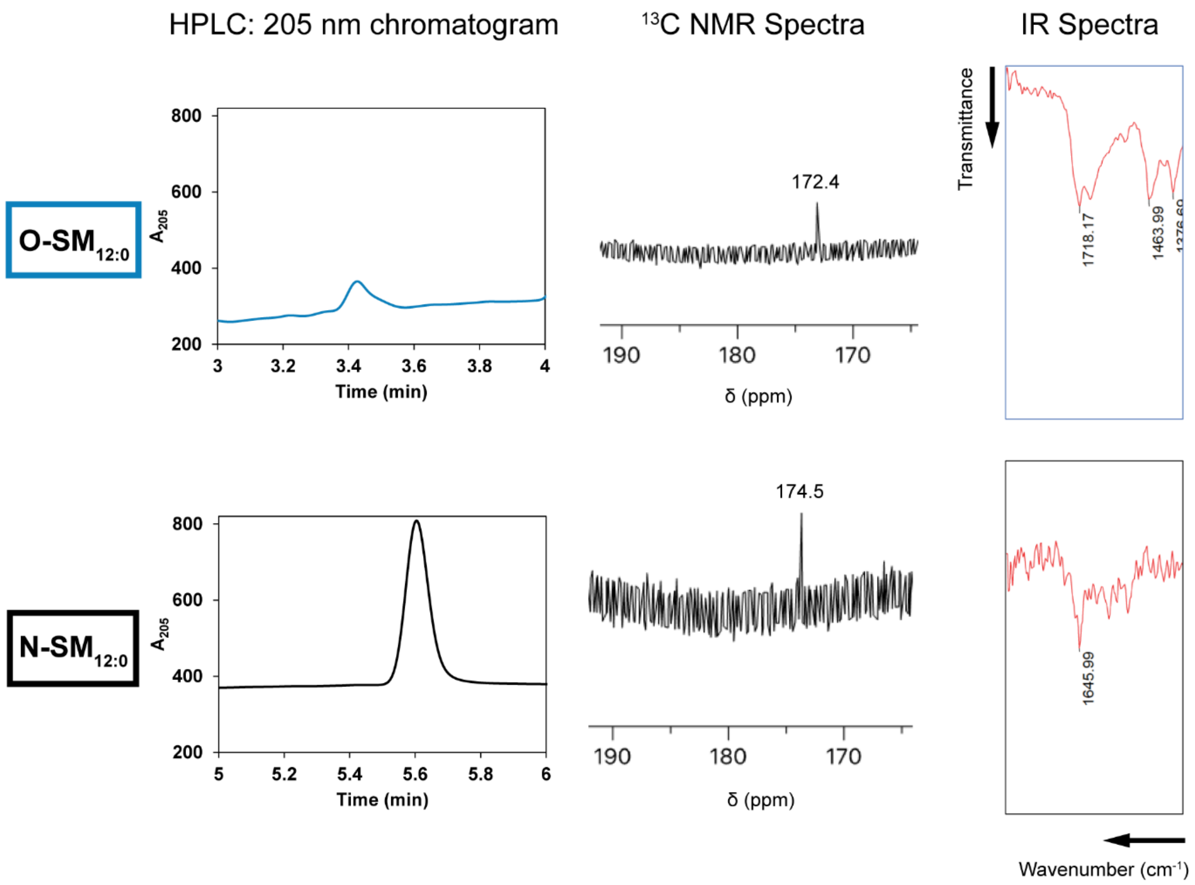


**Figure S2.** Comparison of 205 nm chromatograms (20 nmol each), $^{13}C$ NMR spectra (carbonyl carbon peaks), and FT-IR spectra of pure **O-SM$_{12:0}$** (*top* panel) and **N-SM$_{12:0}$** (*bottom* panel).

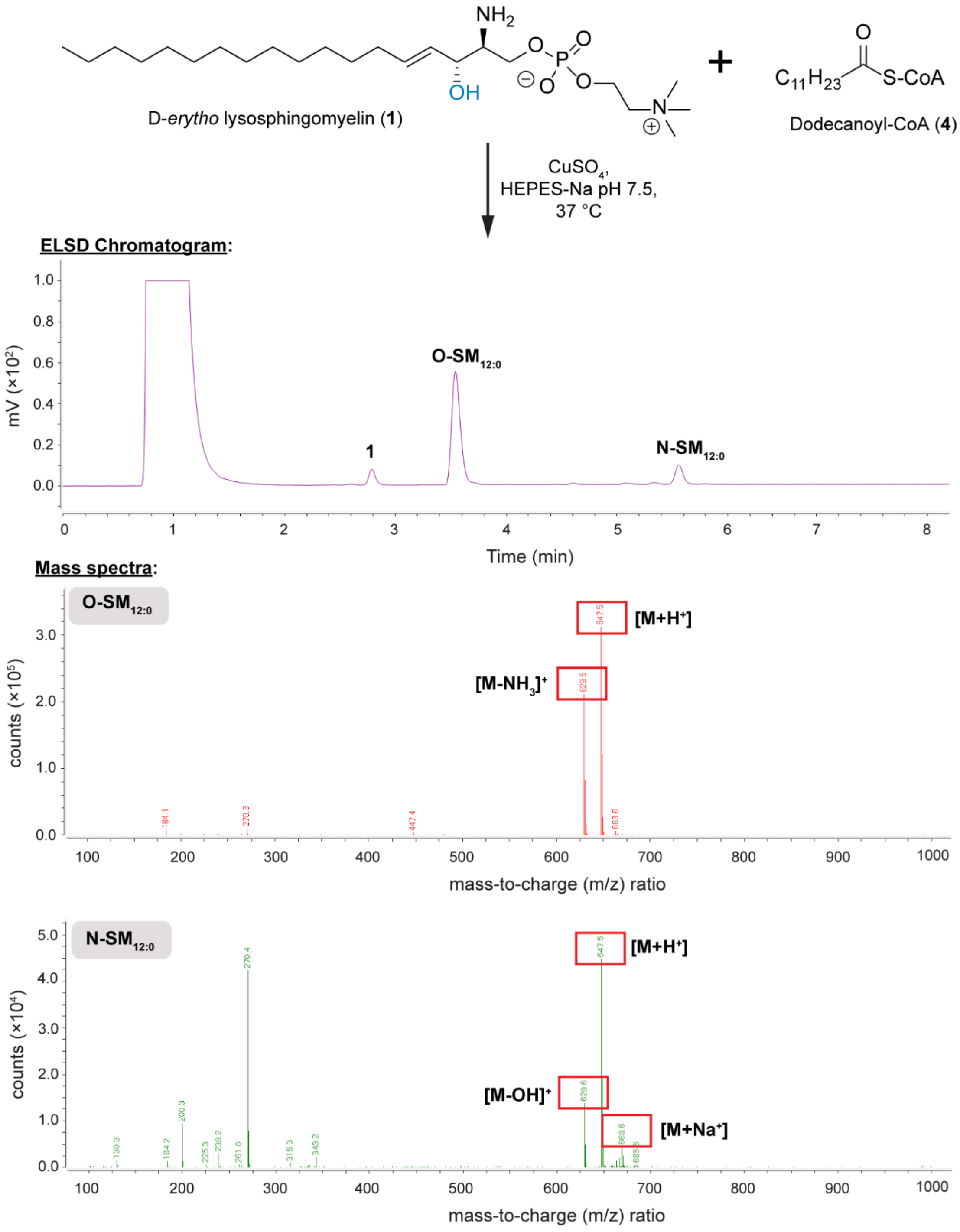


**Figure S3**. HPLC-ELSD analysis of the reaction of sphingosylphosphorylcholine (**1**, 1 mM) with dodecanoyl-CoA (**4**, 1 mM) in HEPES-Na buffer (50 mM, pH 7.5) at 37 °C. HPLC-ELSD chromatogram of the reaction mixture after 7 h and mass spectra of the acylated species are shown. **O-SM$_{12:0}$**: $R_t$ = 3.6 min, MS peaks (m/z) – 647.5 ([M+H$^+$]), 629.5 ([M-NH$_3$]$^+$); **N-SM$_{12:0}$**: $R_t$ = 5.6 min, MS peaks (m/z) – 669.6 ([M+Na$^+$]), 647.5 ([M+H$^+$]), 629.6 ([M-OH]$^+$).

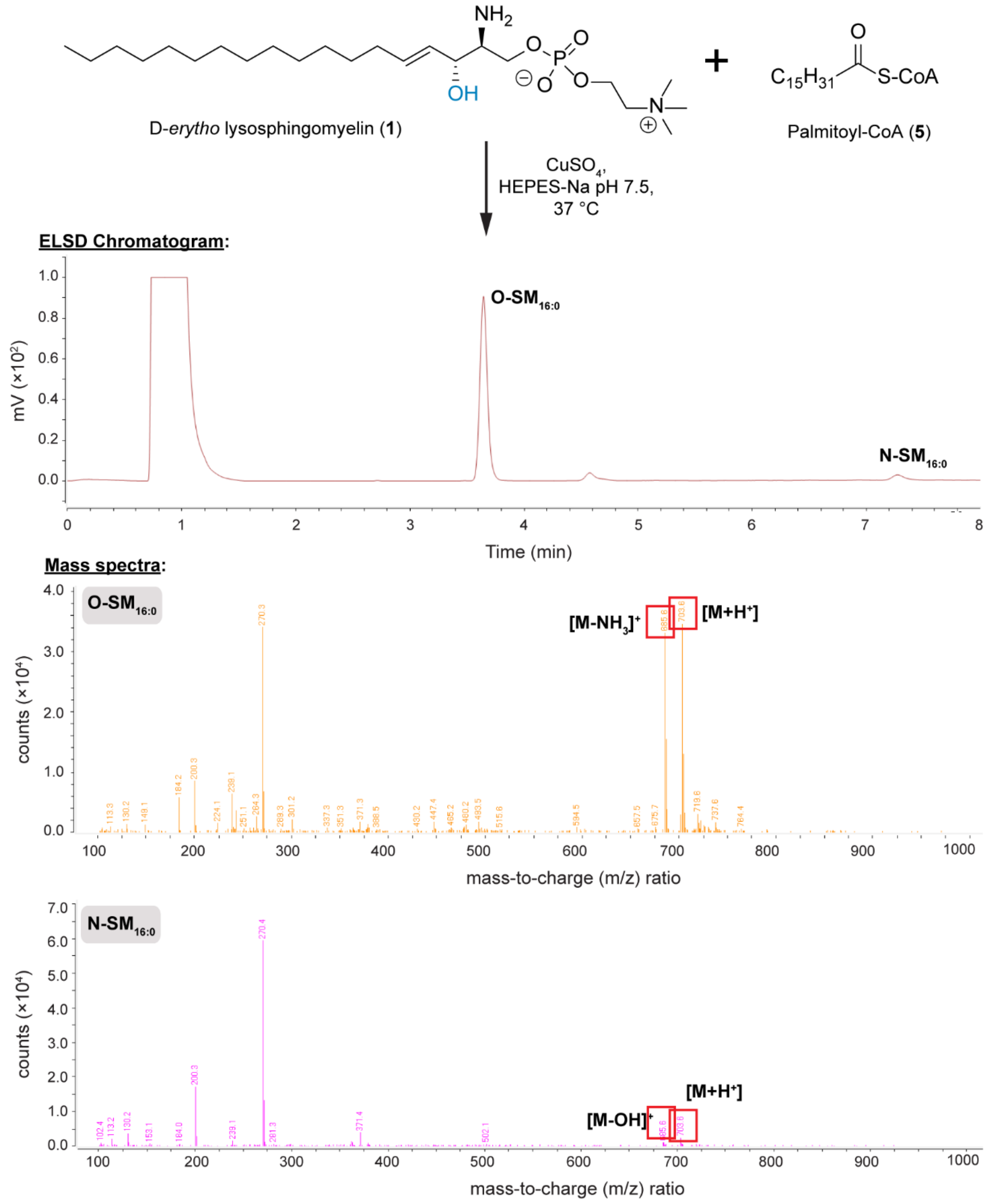


**Figure S4**. HPLC-ELSD analysis of the reaction of sphingosylphosphorylcholine (**1**, 1 mM) with palmitoyl-CoA (**5**, 1 mM) in HEPES-Na buffer (50 mM, pH 7.5) at 37 °C. HPLC-ELSD chromatogram of the reaction mixture after 24 h and mass spectra of the acylated species are shown. **O-SM$_{16:0}$**: $R_t$ = 3.6 min, MS peaks (m/z) – 703.6 ([M+H$^+$]), 685.6 ([M-NH$_3$]$^+$); **N-SM$_{16:0}$**: $R_t$ = 7.5 min, MS peaks (m/z) – 703.6 ([M+H$^+$]), 685.6 ([M-OH]$^+$).

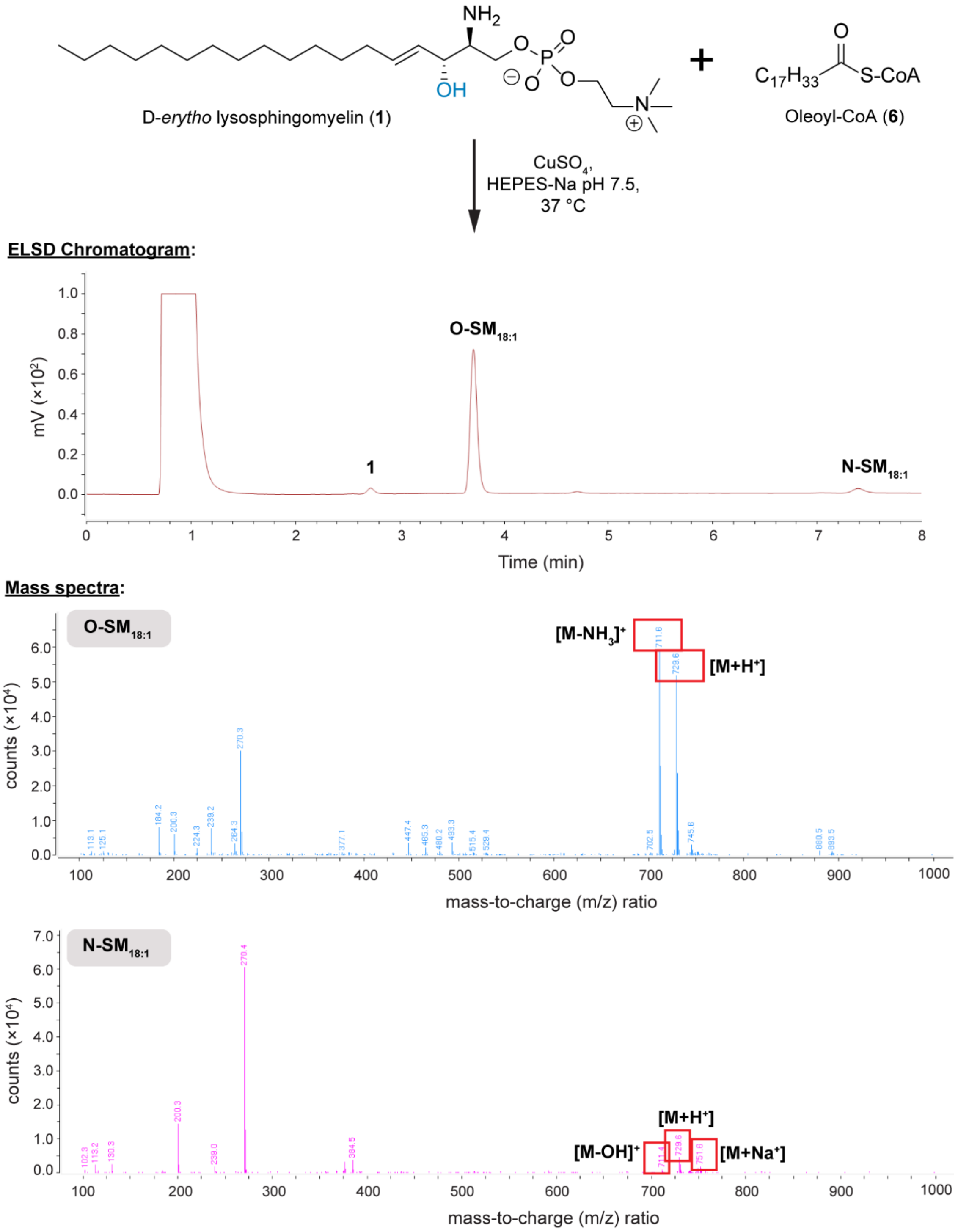


**Figure S5**. HPLC-ELSD analysis of the reaction of sphingosylphosphorylcholine (**1**, 1 mM) with oleoyl-CoA (**6**, 1 mM) in HEPES-Na buffer (50 mM, pH 7.5) at 37 °C. HPLC-ELSD chromatogram of the reaction mixture after 8 h and mass spectra of the acylated species are shown. **O-SM$_{18:1}$**: $R_t$ = 3.8 min, MS peaks (m/z) – 729.6 ([M+H$^+$]), 711.6 ([M-NH$_3$]$^+$); **N-SM$_{18:1}$**: $R_t$ = 7.5 min, MS peaks (m/z) – 751.6 ([M+Na$^+$]), 729.6 ([M+H$^+$]), 711.4 ([M-OH]$^+$).

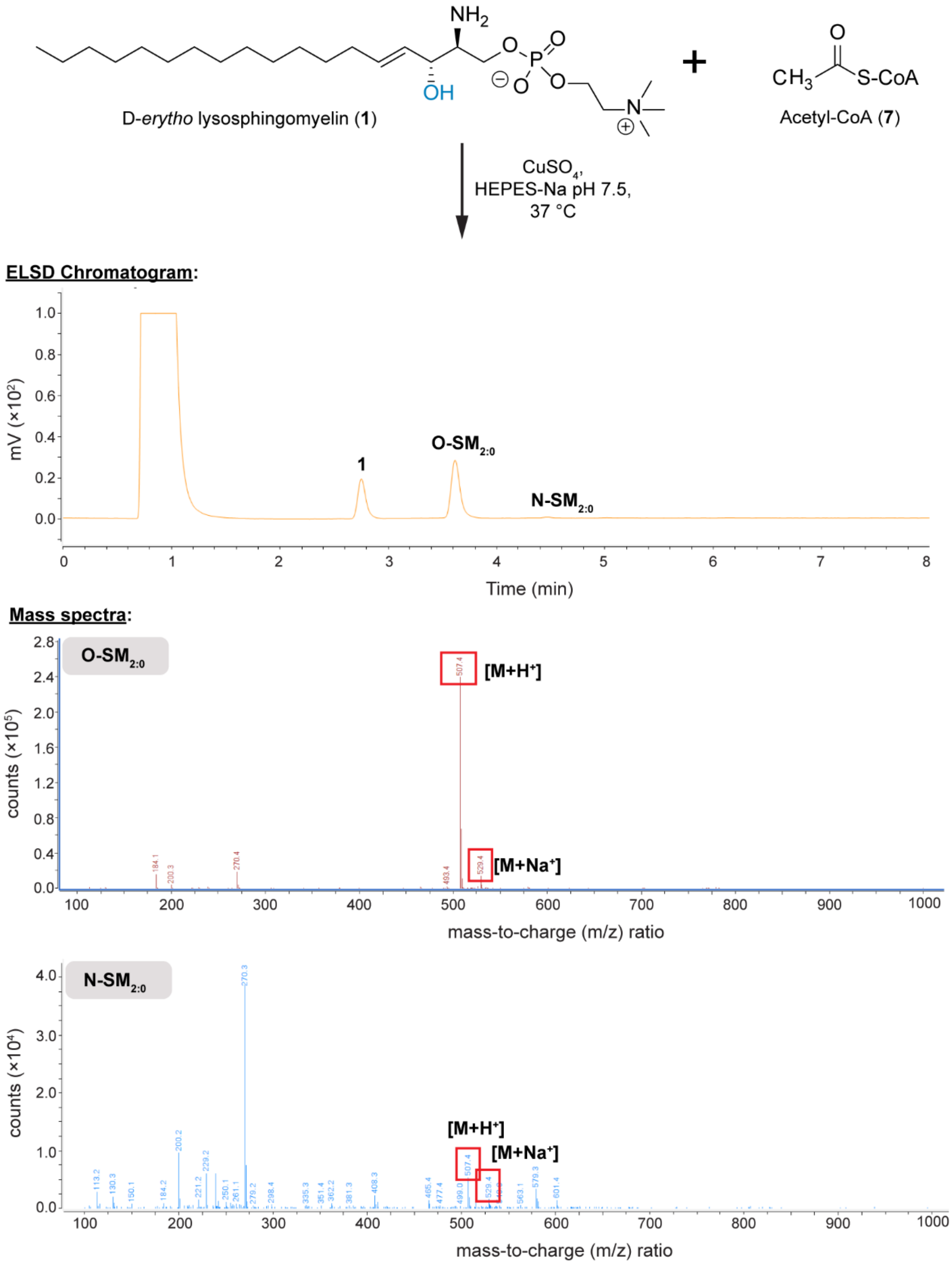


**Figure S6**. HPLC-ELSD analysis of the reaction of sphingosylphosphorylcholine (**1**, 1 mM) with acetyl-CoA (**7**, 5 mM) in HEPES-Na buffer (50 mM, pH 7.5) at 37 °C. HPLC-ELSD chromatogram of the reaction mixture after 24 h and mass spectra of the acylated species are shown. **O-SM$_{2:0}$**: $R_t$ = 3.6 min, MS peaks (m/z) – 507.4 ([M+H$^+$]), 529.4 ([M+Na$^+$]); **N-SM$_{2:0}$**: $R_t$ = 4.5 min, MS peaks (m/z) – 507.4 ([M+H$^+$]), 529.4 ([M+Na$^+$]).

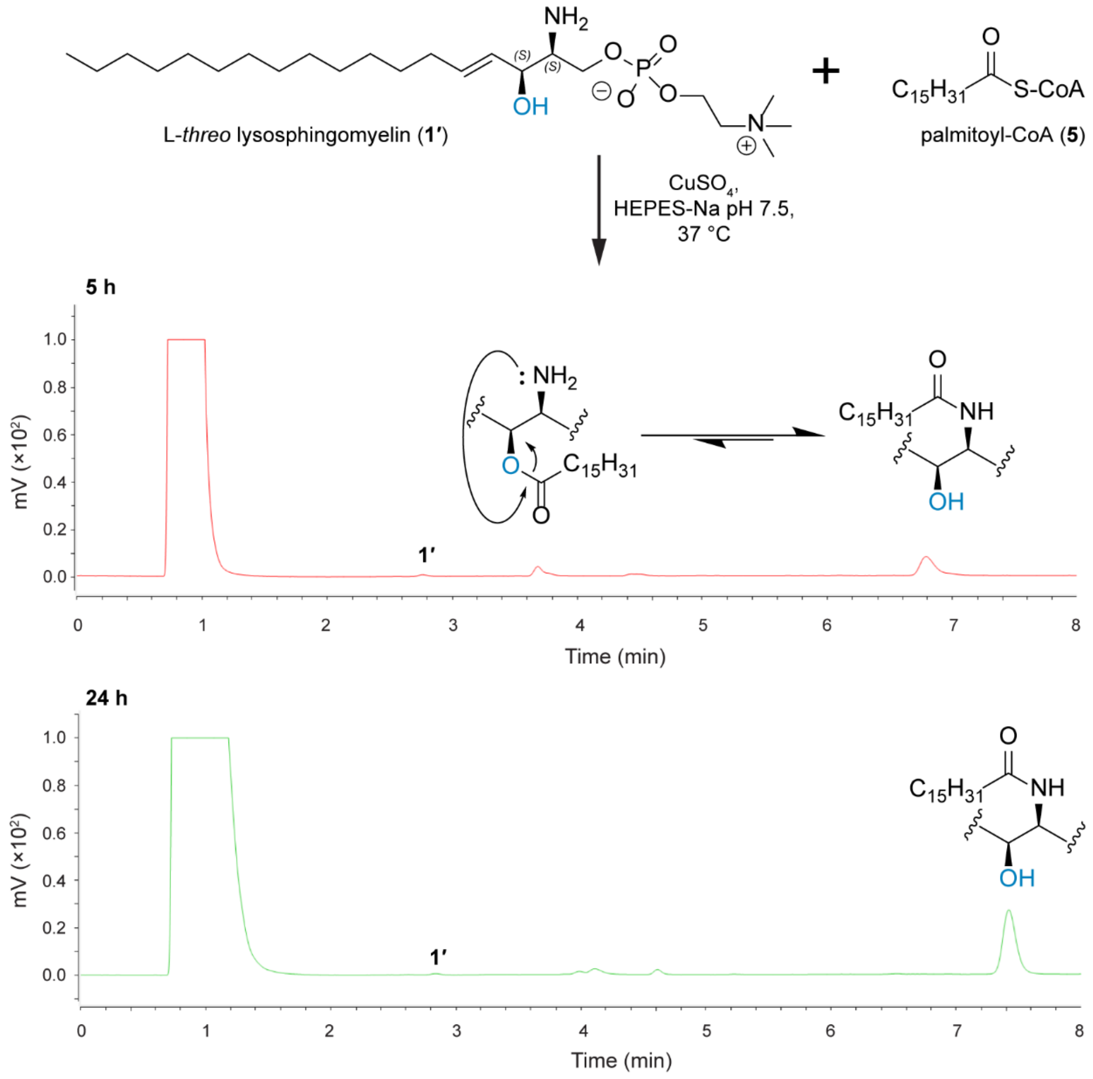


**Figure S7**. HPLC-ELSD analysis of the reaction L-*threo* lysosphingomyelin (**1′**, 1 mM) with palmitoyl-CoA (**5**, 1 mM) at pH 7.5 (50 mM HEPES-Na salt), 37 °C, 24 h. In the ELSD chromatogram of the reaction mixture after 5 h, peaks at retention times 3.8 min and 6.8 min correspond to *O*-acylated and *N*-acylated derivatives of **1′** respectively. In the ELSD chromatogram of the reaction mixture after 24 h, peaks at retention time of 7.4 min corresponds to *N*-acylated derivative of **1′**.

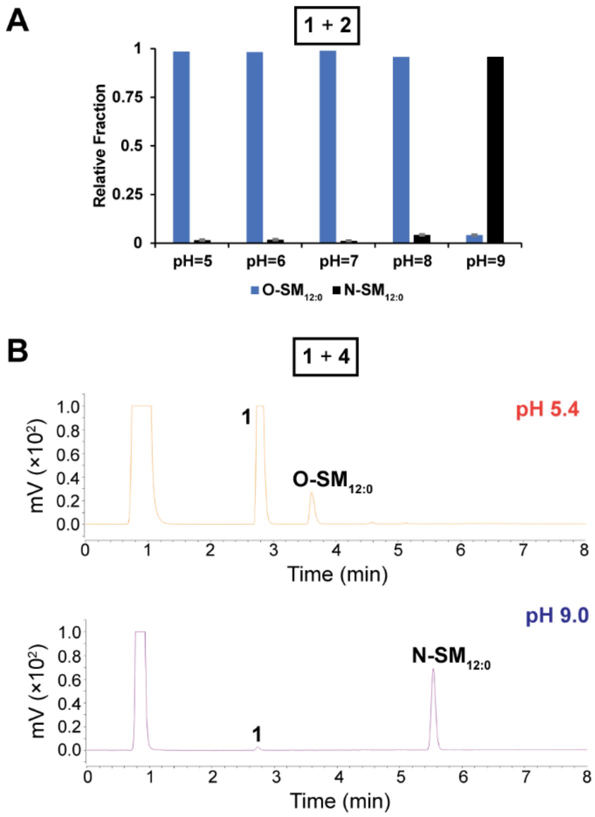


**Figure S8**. pH dependence of *O*- and *N*-acylation of sphingosylphosphorylcholine with adenylates and thioesters. **A**. HPLC-MS analyses of the relative fractions of **O-SM$_{12:0}$** and **N-SM$_{12:0}$** generated at pH 5-9 via the reaction between 1 mM of each of **1** and **2**. Error bars represent standard deviation (*n* = 3). **B**. HPLC-ELSD analysis of the reaction between **1** (1 mM) with **4** (1 mM) at pH 5.4 (50 mM NaOAc/AcOH) and at pH 9.0 (50 mM $NaHCO_3/Na_2CO_3$) at 37 °C after 24 h.

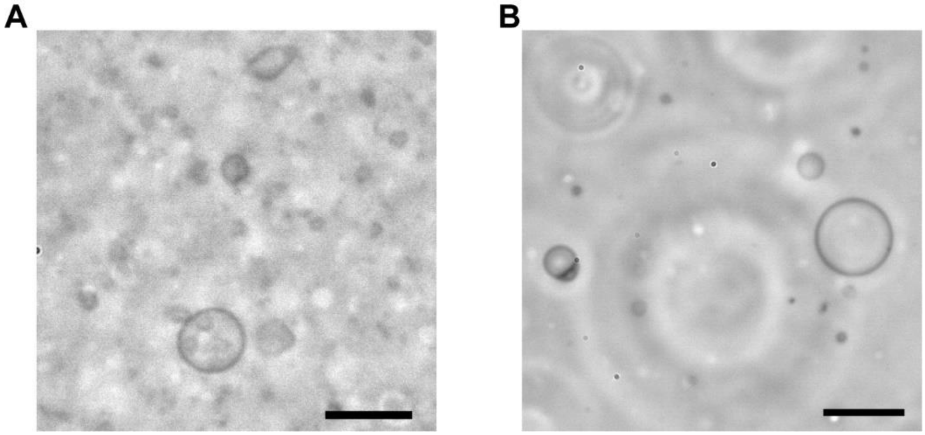


**Figure S9**. Vesicle formation from *in situ* synthesized *O*-acylated sphingomyelin analogues. 1 mM of each of sphingosylphosphorylcholine (**1**) and fatty acyl CoAs (**5** or **6**) were mixed in presence of 50 mM HEPES-Na (pH 7.5) and 0.5 mM $CuSO_4$ and incubated at 37 °C. Representative vesicles are shown corresponding to formation of **A**. **O-SM$_{16:0}$** (scale bar: 15 µm) and **B**. **O-SM$_{18:1}$** (scale bar: 20 µm).

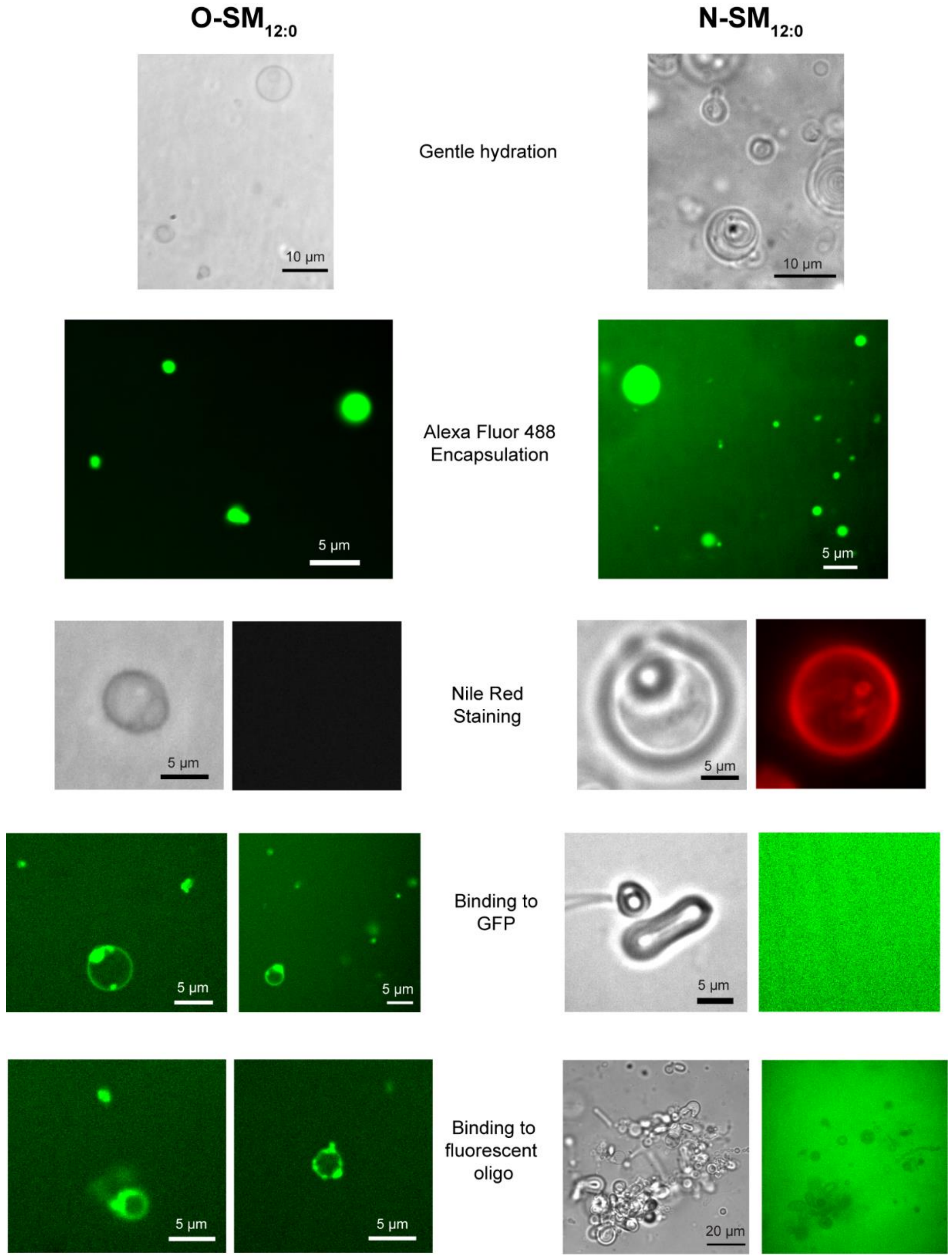


**Figure S10.** Characterization of **O-SM$_{12:0}$** and **N-SM$_{12:0}$** vesicles using bright field and fluorescence microscopy.

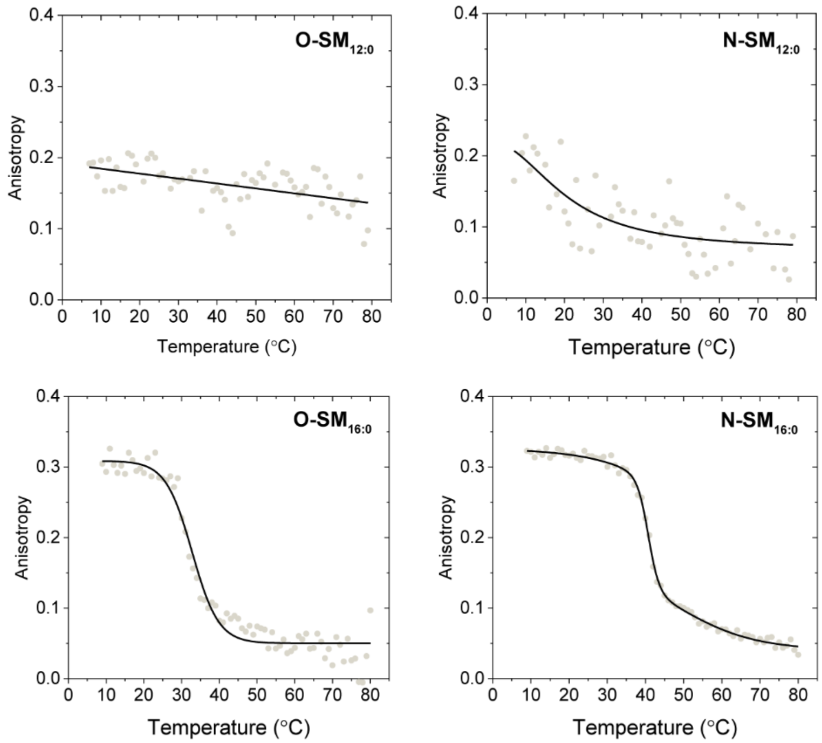


**Figure S11.** Study of gel-to-fluid phase transition behavior based on DPH anisotropy. In a typical measurement, an aqueous dispersion of 1 mM of the lipid containing 0.1 mol% DPH in water is taken in a temperature-controlled cuvette and excited ($\lambda_{ex}$) at 360 nm and emission ($\lambda_{em}$) is measured at 430 nm. The value of steady state anisotropy (r) is calculated as $r = (I_{\parallel} - I_{\perp})/(I_{\parallel} + 2I_{\perp})$, where $I_{\parallel}$ and $I_{\perp}$ correspond to emission intensities parallel or perpendicular to the direction of polarization of the excitation light respectively. The bold lines represent linear fit for **O-SM$_{12:0}$** and sigmoidal fits of the data points for **N-SM$_{12:0}$**, **O-SM$_{16:0}$**, and **N-SM$_{16:0}$**. In the case of **O-SM$_{12:0}$**, no gel-to-fluid phase transition was observed over the temperature range studied. For **N-SM$_{12:0}$**, **O-SM$_{16:0}$**, and **N-SM$_{16:0}$**, gel-to-fluid phase transition temperature were measured to be 20.3 °C, 30.9 °C, and 40.7 °C respectively.

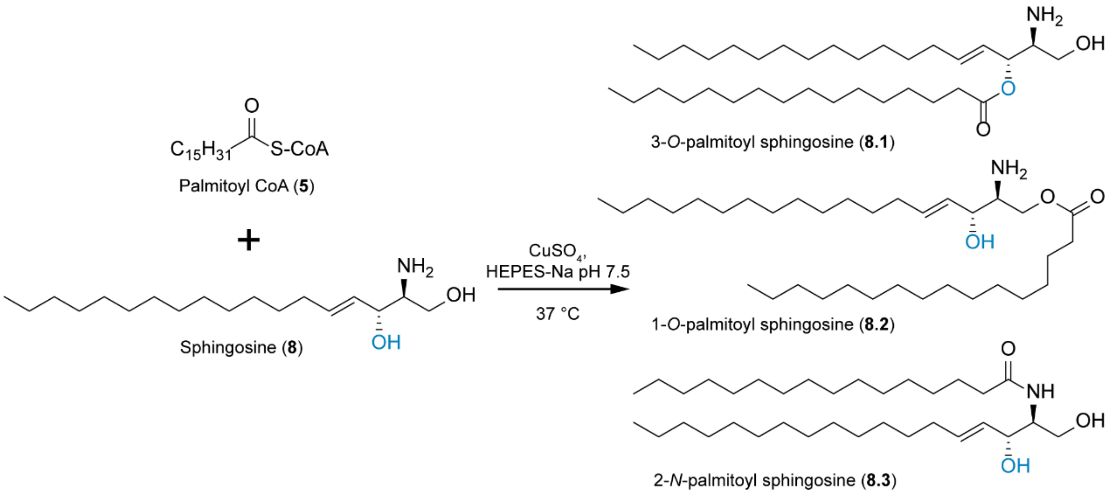


**ELSD Chromatogram**:

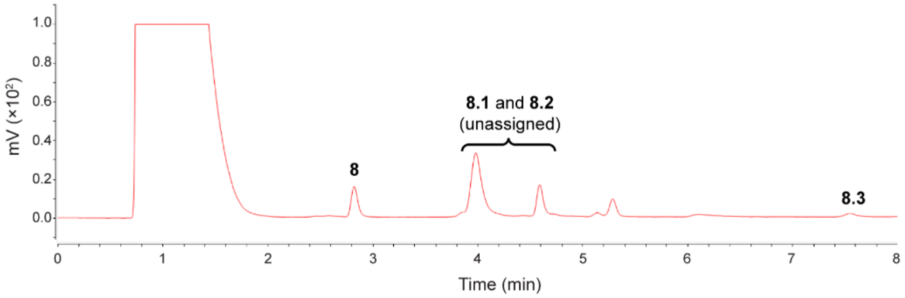


**Mass spectra**:

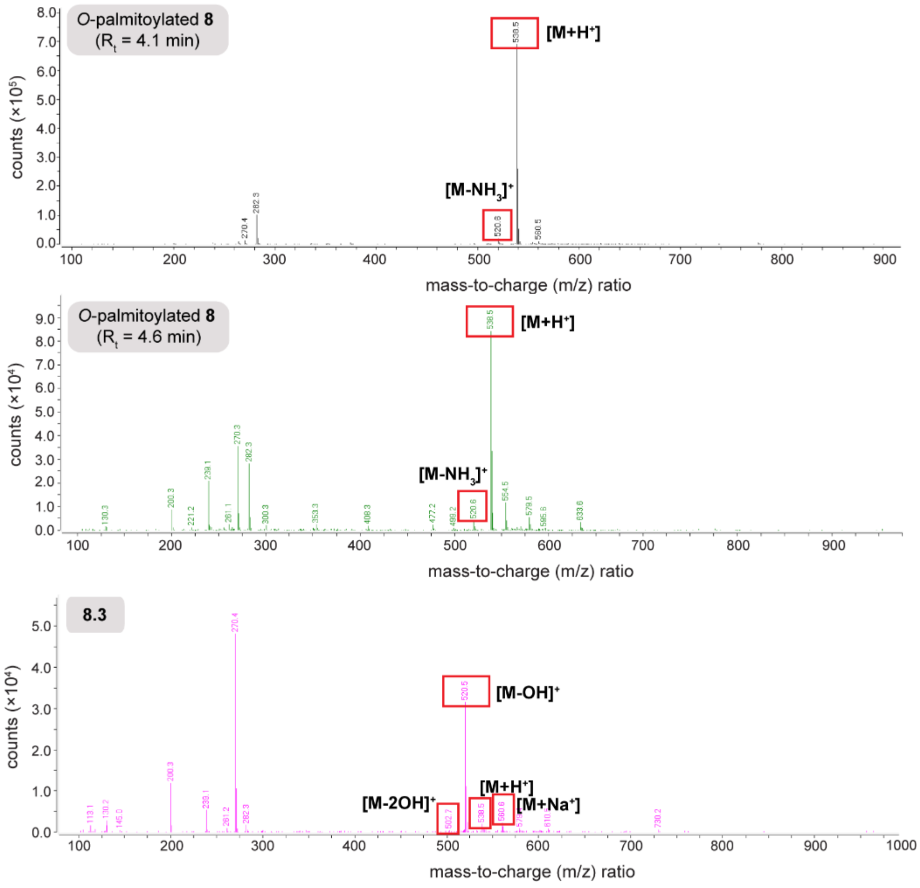

**Figure S12**. HPLC-ELSD analysis of the reaction of sphingosine (**8**, 1 mM) with palmitoyl-CoA (**5**, 2 mM) in HEPES-Na buffer (50 mM, pH 7.5) at 37 °C. HPLC-ELSD chromatogram of the reaction mixture after 24 h and mass spectra of the acylated species are shown. 1$^{st}$ *O*-palmitoylated product: $R_t$ = 4.1 min, MS peaks (m/z) – 538.5 ([M+H$^+$]), 520.5 ([M-NH$_3$]$^+$]); 2$^{nd}$ *O*-palmitoylated product: $R_t$ = 4.6 min, MS peaks (m/z) – 538.5 ([M+H$^+$]), 520.5 ([M-NH$_3$]$^+$]); **8.3**: $R_t$ = 7.5 min, MS peaks (m/z) – 560.6 ([M+Na$^+$]), 538.5 ([M+H$^+$]), 520.5 ([M-OH]$^+$), 502.7 ([M-2OH]$^+$).

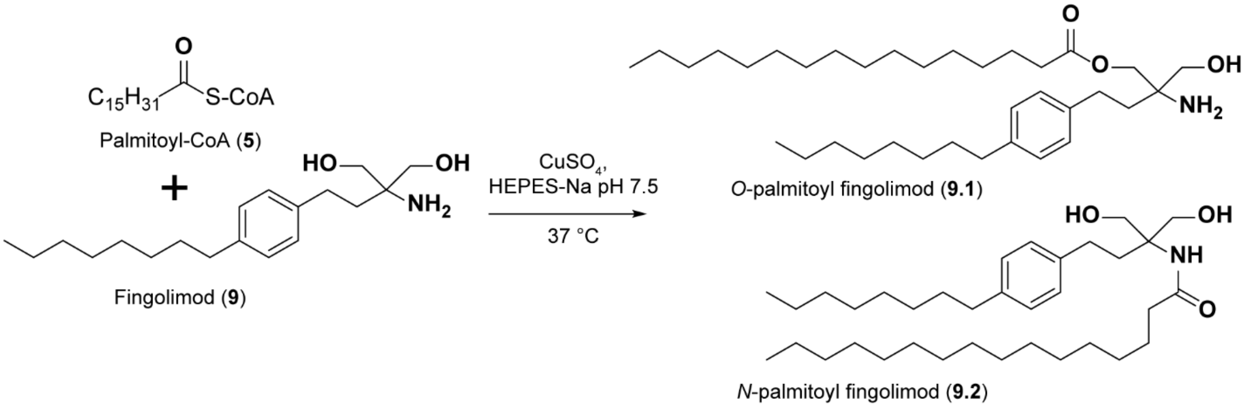


**<u>ELSD Chromatogram</u>:**

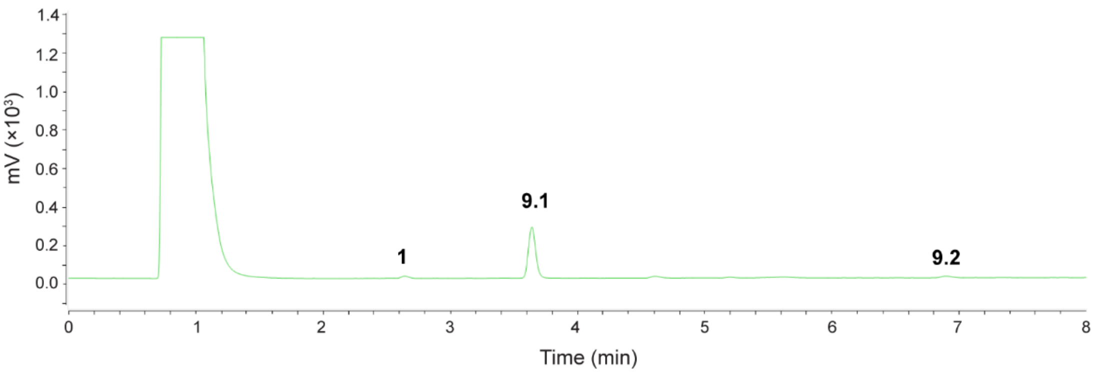


**<u>Mass spectra</u>:**

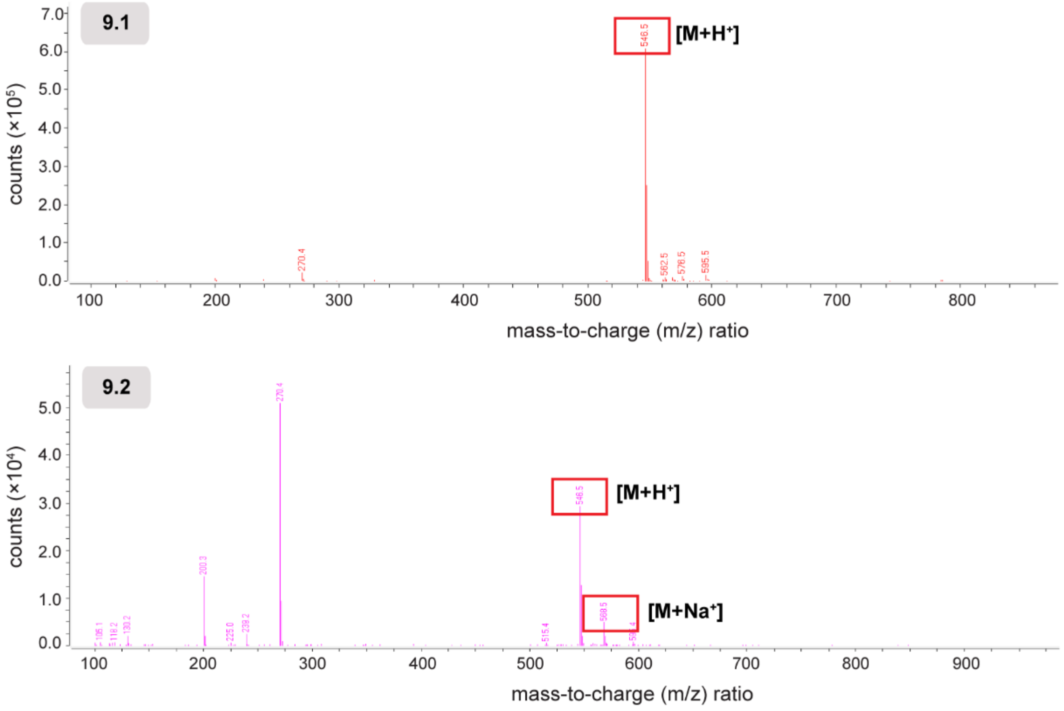

**Figure S13**. HPLC-ELSD analysis of the reaction of fingolimod (**9**, 1 mM) with palmitoyl-CoA (**5**, 2 mM) in HEPES-Na buffer (50 mM, pH 7.5) at 37 °C. HPLC-ELSD chromatogram of the reaction mixture after 24 h and mass spectra of the acylated species are shown. **9.1**: $R_t$ = 3.7 min, MS peaks (m/z) – 546.5 ([M+H$^+$]); **9.2**: $R_t$ = 7.0 min, MS peaks (m/z) – 546.5 ([M+H$^+$]), 568.5 ([M+Na$^+$]).

**A.** Prebiotic synthesis of 2-hydroxy *n*-alkylamines:

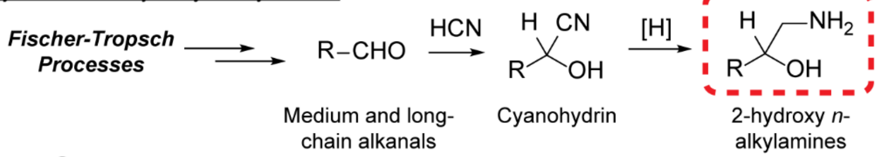


**B.**

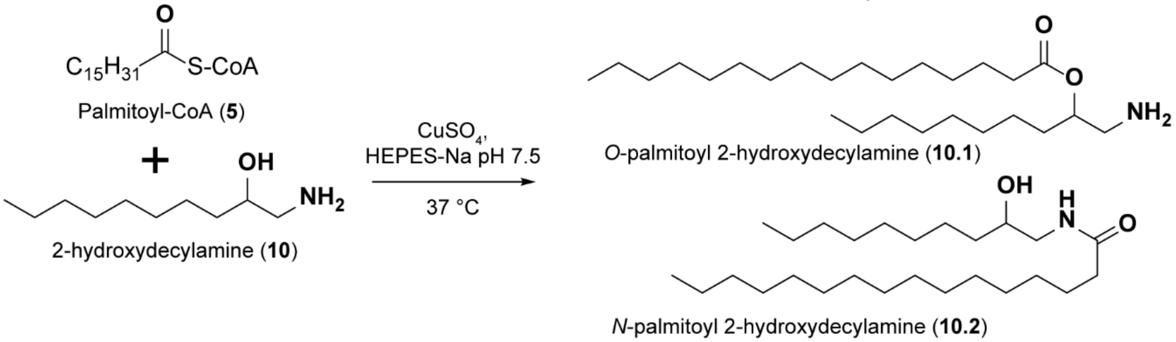


**ELSD Chromatogram:**

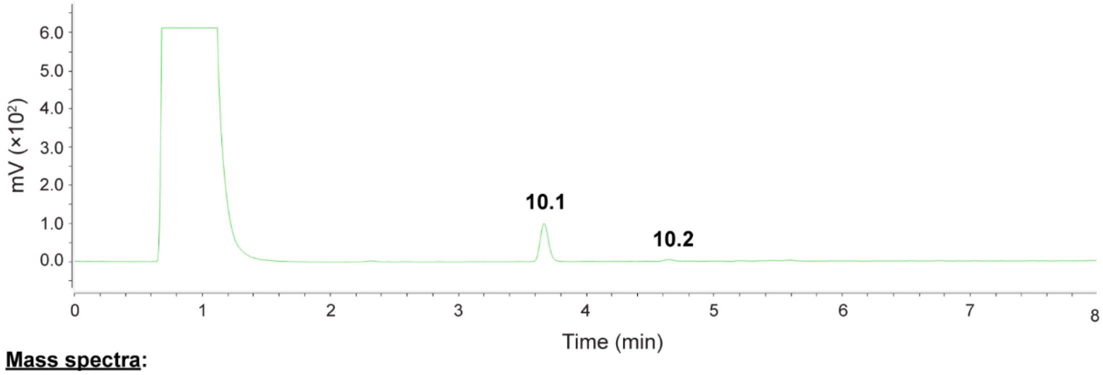


**Mass spectra:**

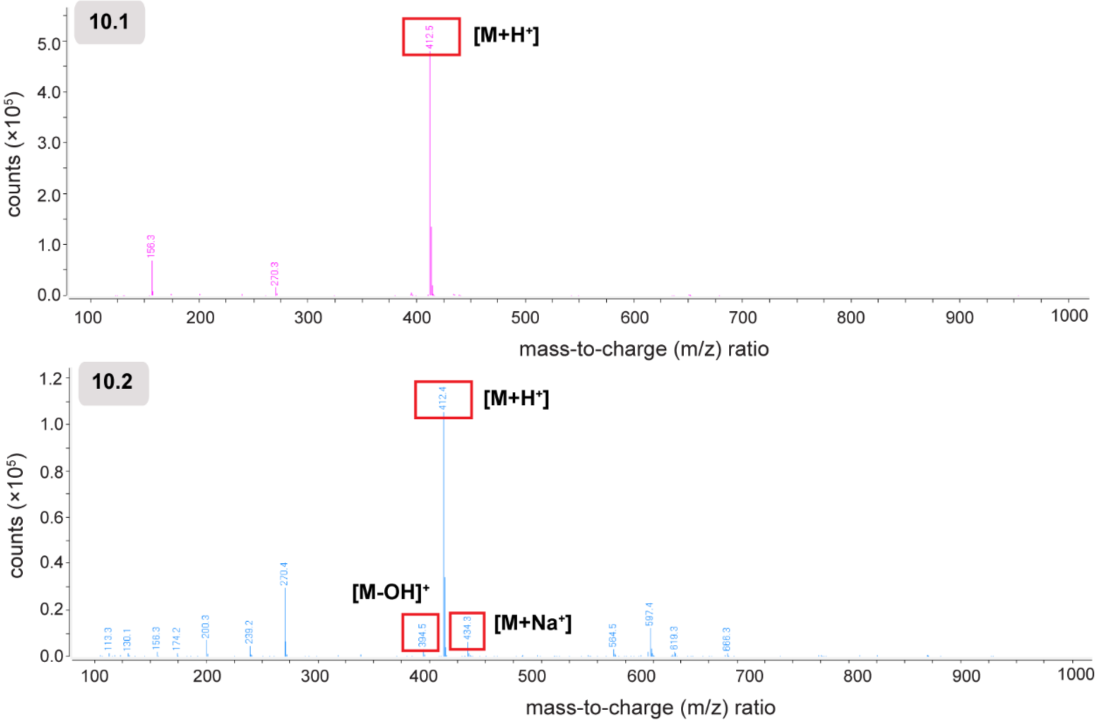

**Figure S14**. Selective *O*-acylation of a prebiotically plausible 1,2-amino alcohol amphiphile. **A**. Scheme of prebiotically plausible synthesis of 2-hydroxy *n*-alkylamines.[11] **B**. HPLC-ELSD analysis of the reaction of 2-hydroxydecylamine (**10**, 1 mM) with palmitoyl-CoA (**5**, 1 mM) in HEPES-Na buffer (50 mM, pH 7.5) at 37 °C. HPLC-ELSD chromatogram of the reaction mixture after 38 h and mass spectra of the acylated species are shown. **10.1**: $R_t$ = 3.7 min, MS peaks (m/z) – 412.5 ([M+H$^+$]); **10.2**: $R_t$ = 5.7 min, MS peaks (m/z) – 434.3 ([M+Na$^+$]), 412.4 ([M+H$^+$]), 394.5 ([M-OH]$^+$). The retention time of **10.2** was further verified with the pure compound accessed by organic synthesis.

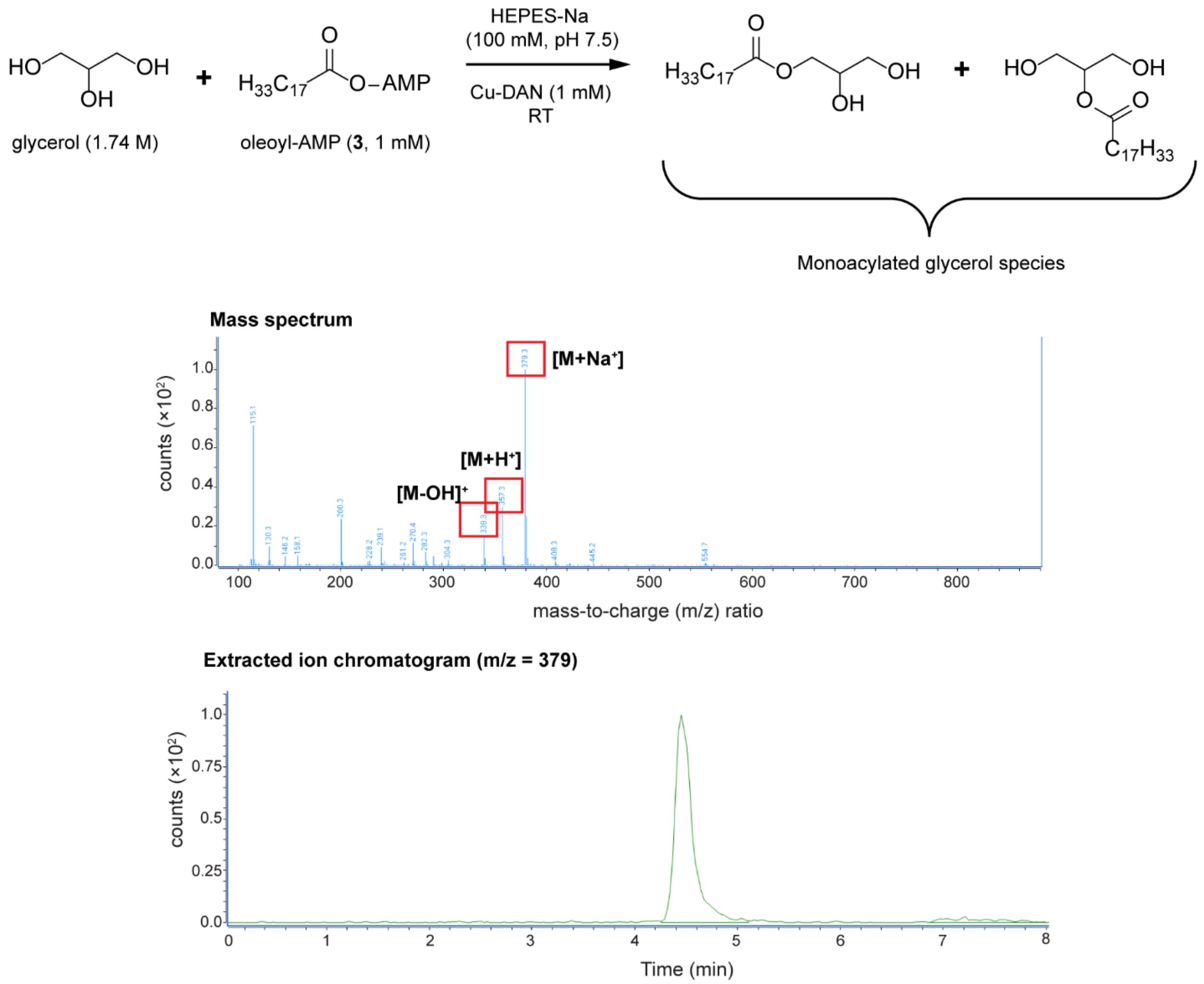


**Figure S15**. Metal ion-directed monoacylation of glycerol in aqueous media. A reaction is set up by adding glycerol (1.71 M), oleoyl AMP (**3**, 1 mM), and Cu-**DAN** (1 mM) in HEPES-Na (100 mM, pH 7.5) at RT and the reaction is analyzed by LC-MS after 40 min. Mass spectrum corresponding to formation of monooleoyl glycerol product is shown. MS peaks (m/z) – 379.3 ([M+Na$^+$]), 357.3 ([M+H$^+$]), 339.3 ([M-OH]$^+$). The extracted ion chromatogram corresponding to the major ion species m/z = 379.3 ([M+Na$^+$]) is shown.

## Supplementary References


(1) Bhattacharya, A.; Brea, R. J.; Niederholtmeyer, H.; Devaraj, N. K. A Minimal Biochemical Route towards *de novo* Formation of Synthetic Phospholipid Membranes. *Nat. Commun.* **2019**, *10*, 300.

(2) Gulbins, E. *Sphingolipids: Basic Science and Drug Development*; **2013**; Vol. 215.

(3) Taha, T. A.; Mullen, T. D.; Obeid, L. M. A House Divided: Ceramide, Sphingosine, and Sphingosine-1-Phosphate in Programmed Cell Death. *Biochim. Biophys. Acta* **2006**, *1758* (12), 2027–2036.

(4) Bartke, N.; Hannun, Y. A. Bioactive Sphingolipids: Metabolism and Function. *J. Lipid Res.* **2009**, *50*, S91–S96.

(5) Miazek, K.; Lebecque, S.; Hamaidia, M.; Paul, A.; Danthine, S.; Willems, L.; Frederich, M.; De Pauw, E.; Deleu, M.; Richel, A.; Goffin, D. Sphingolipids: Promising Lipid-Class Molecules with Potential Applications for Industry. A Review. *Biotechnol. Agron. Soc. Environ.* **2016**, *20*, 321–336.

(6) Rabionet, M.; Bayerle, A.; Marsching, C.; Jennemann, R.; Gröne, H. J.; Yildiz, Y.; Wachten, D.; Shaw, W.; Shayman, J. A.; Sandhoff, R. 1-*O*-Acylceramides Are Natural Components of Human and Mouse Epidermis. *J. Lipid Res.* **2013**, *54* (12), 3312–3321.

(7) Van Overloop, H.; Van Der Hoeven, G.; Van Veldhoven, P. P. *N*-Acyl Migration in Ceramides. *J. Lipid Res.* **2005**, *46* (4), 812–816.

(8) Malyarenko, T. V.; Kicha, A. A.; Stonik, V. A.; Ivanchina, N. V. Sphingolipids of Asteroidea and Holothuroidea: Structures and Biological Activities. *Mar. Drugs* **2021**, *19* (6), 330.

(9) Kramer, J. K. G.; Blackwell, B. A.; Dugan, M. E. R.; Sauer, F. D. Identification of a New Sphingolipid 3-*O*-Acyl-D-*Erythro*-Sphingomyelin in Newborn Pig and Infant Plasma. *Biochim. Biophys. Acta* **1996**, *1303* (1), 47–55.

(10) Sergelius, C.; Slotte, J. P. Membrane Properties of and Cholesterol's Interactions with a Biologically Relevant Three-Chain Sphingomyelin: 3-*O*-Palmitoyl-*N*-Palmitoyl-D-Erythro-Sphingomyelin. *Biochim. Biophys. Acta* **2011**, *1808* (12), 2841–2848.

(11) Mullen, L. B.; Sutherland, J. D. Formation of Potentially Prebiotic Amphiphiles by Reaction of *β*-Hydroxy-*n*-Alkylamines with Cyclotriphosphate. *Angew. Chem. Int. Ed.* **2007**, *46* (22), 4166–4168.

# NMR Spectra

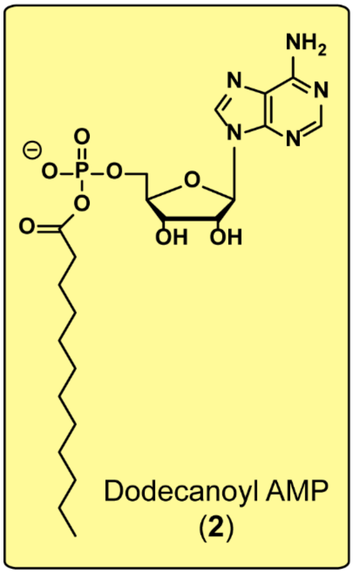

Dodecanoyl AMP
(**2**)

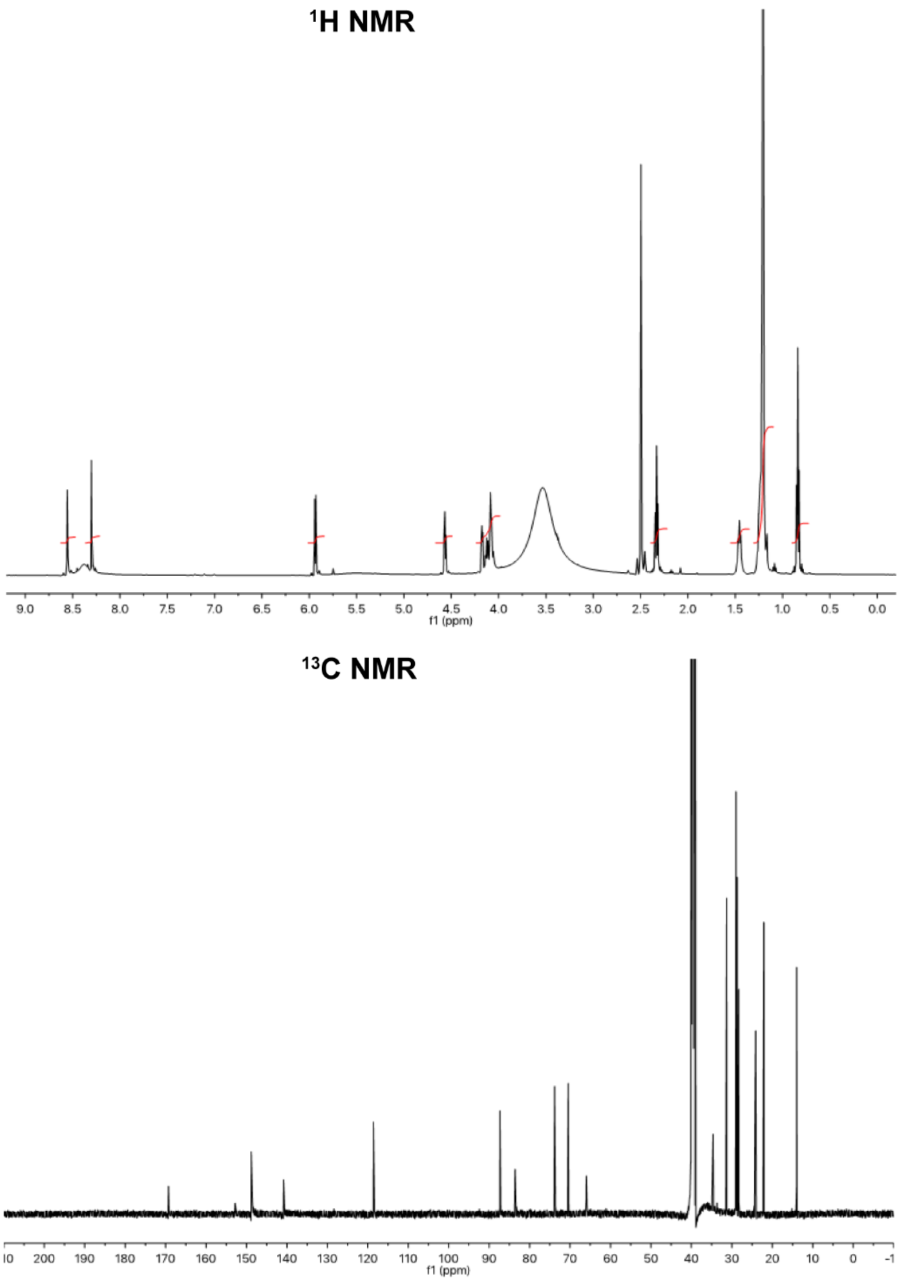


$^1$H (*top*) and $^{13}$C (*bottom*) NMR spectra of compound **2**.

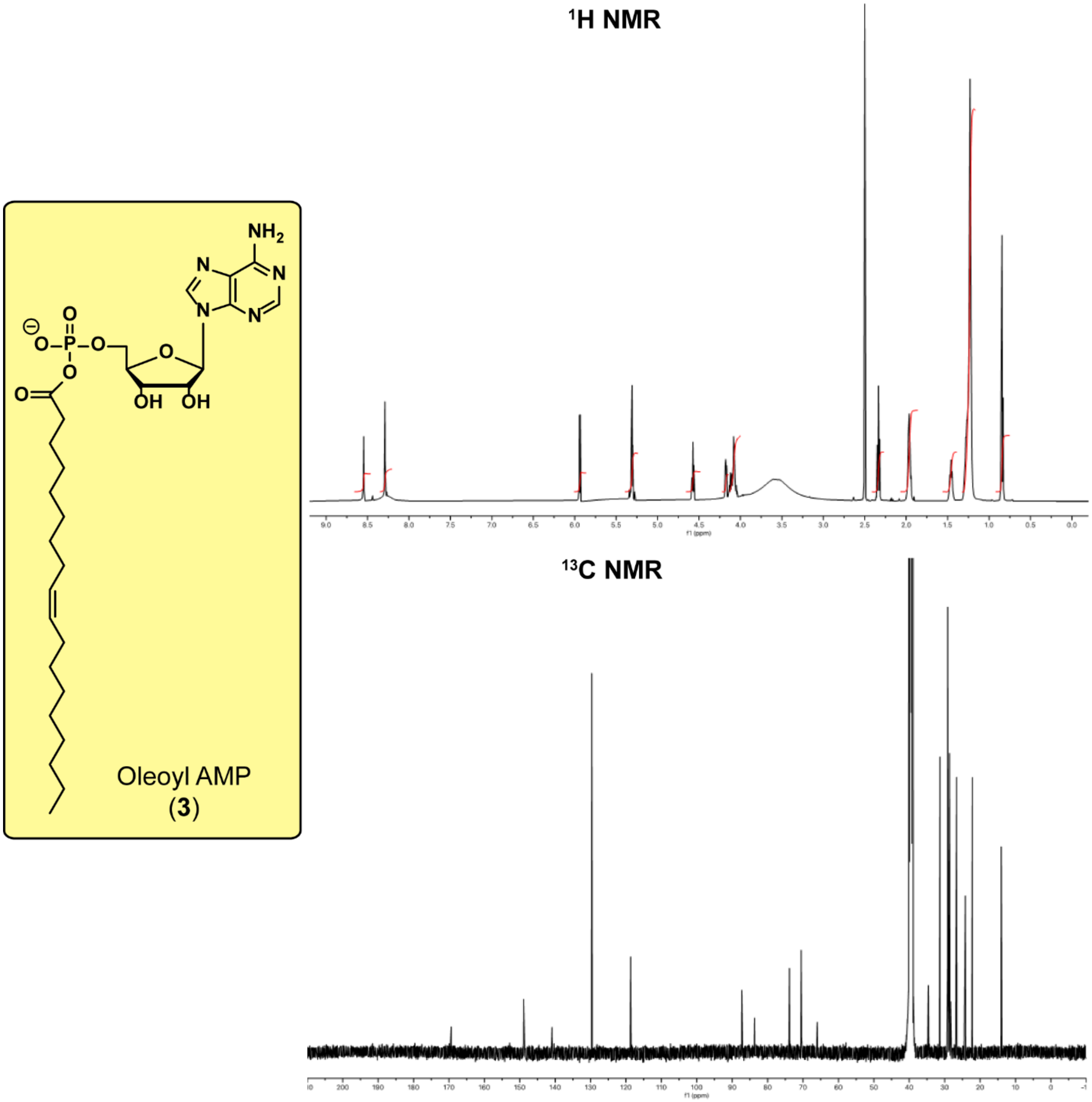


$^{1}$H (*top*) and $^{13}$C (*bottom*) NMR spectra of compound **3**.

## $^{1}$H NMR

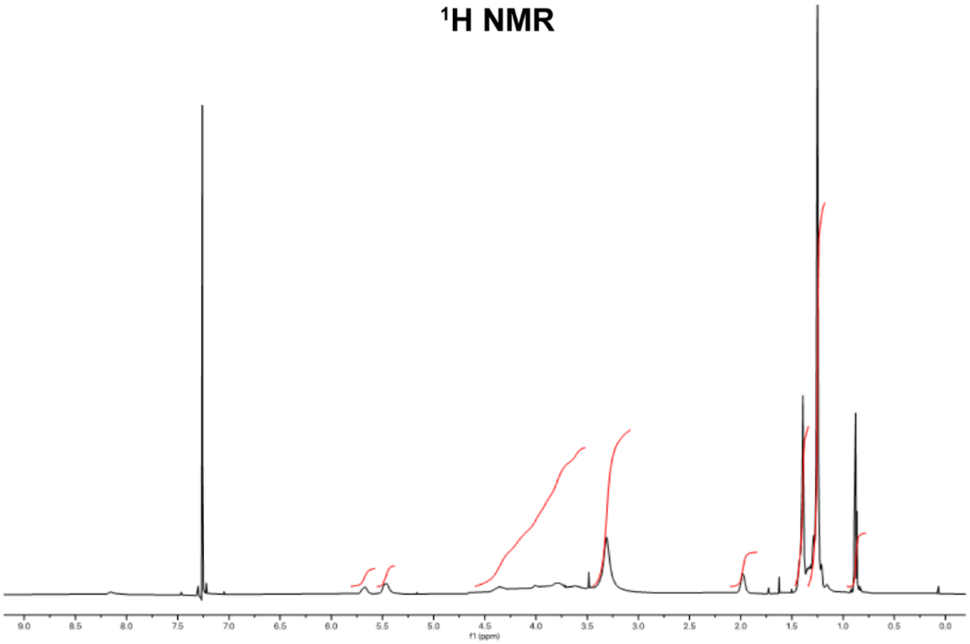

## $^{13}$C NMR

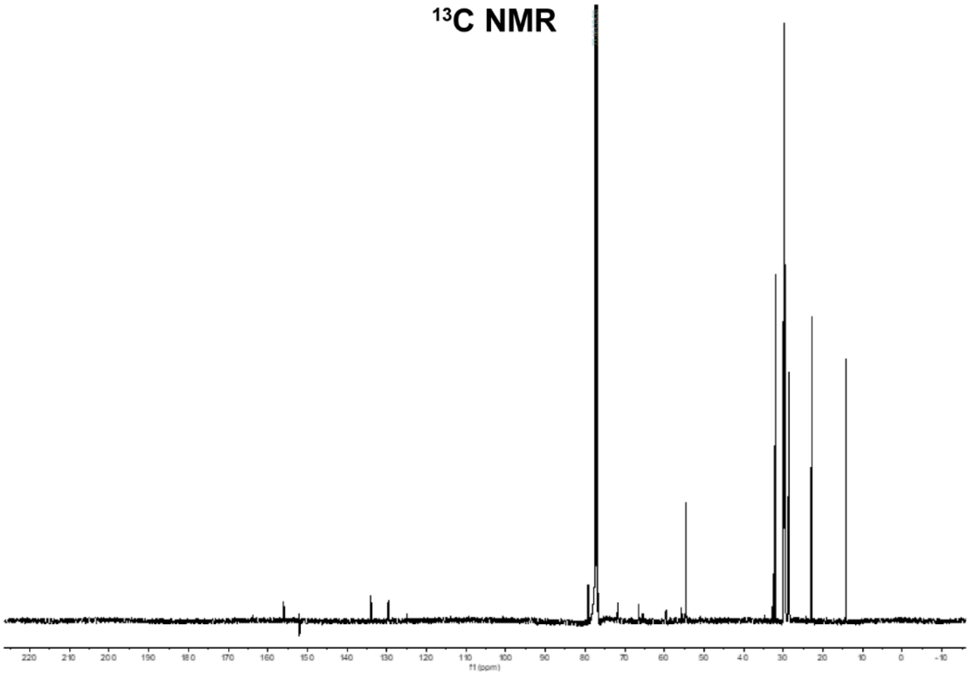

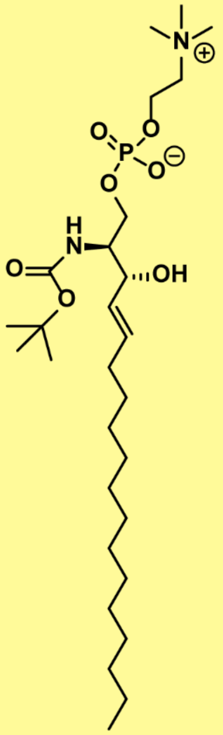

Lyso-SM (Boc)
(**1.1**)

$^{1}$H (*top*) and $^{13}$C (*bottom*) NMR spectra of compound **1.1**.

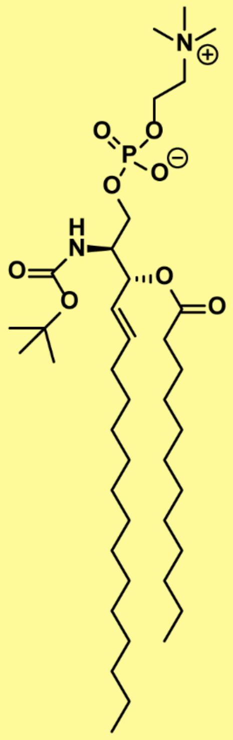

O-SM$_{12:0}$(Boc)
(**1.2**)

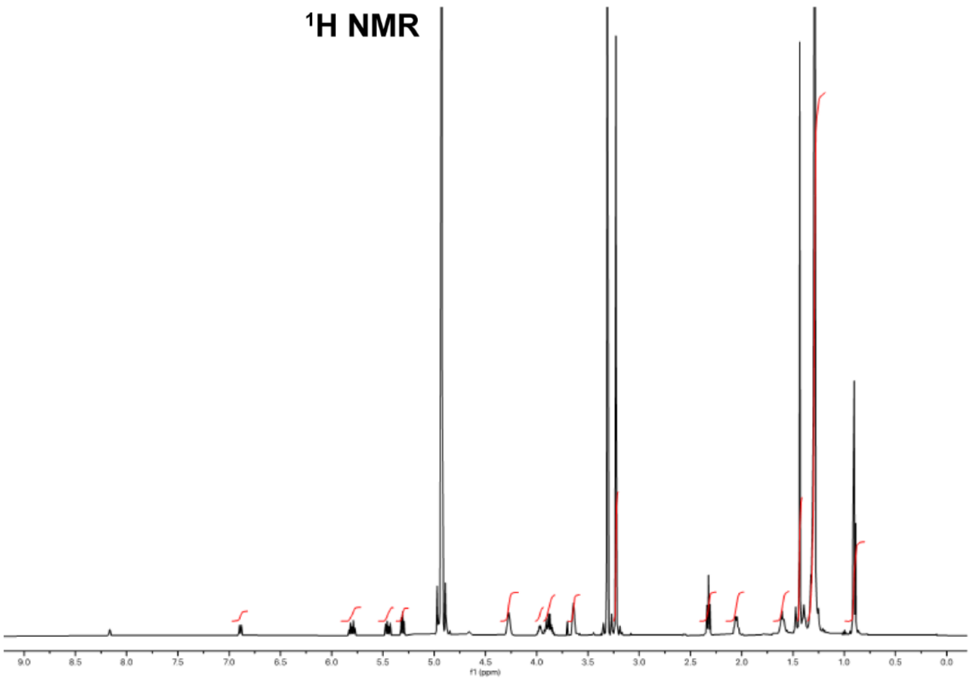


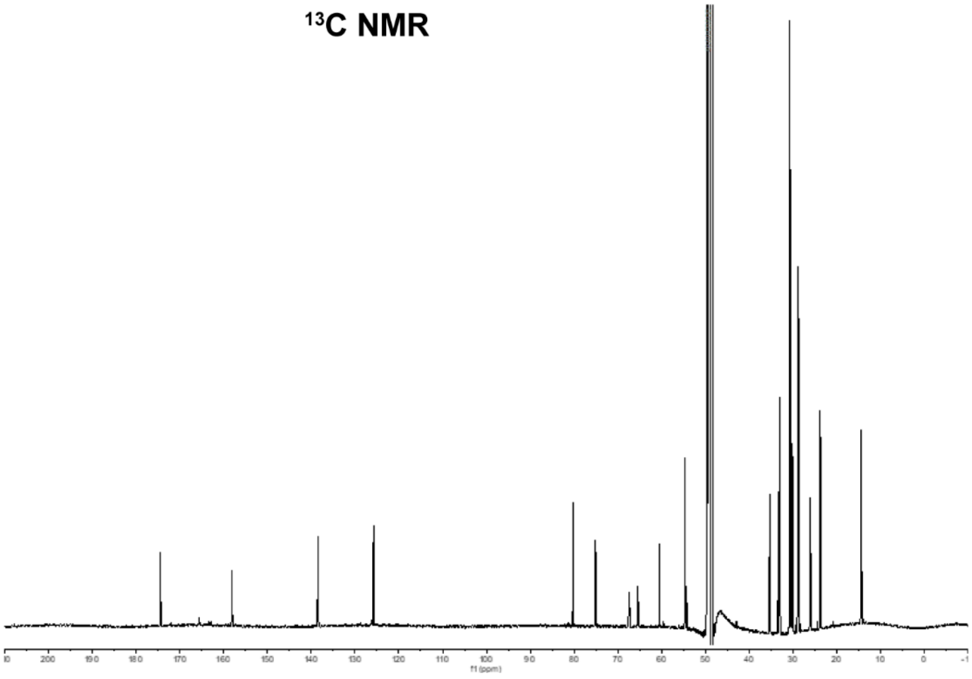


$^{1}$H (*top*) and $^{13}$C (*bottom*) NMR spectra of compound **1.2**.

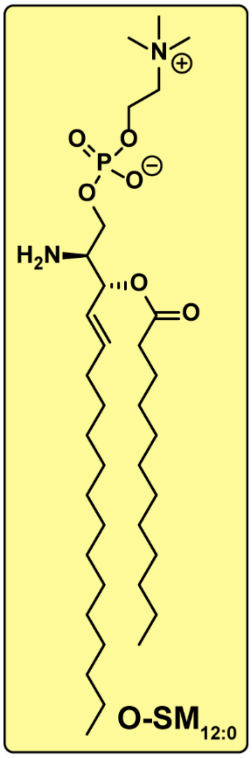


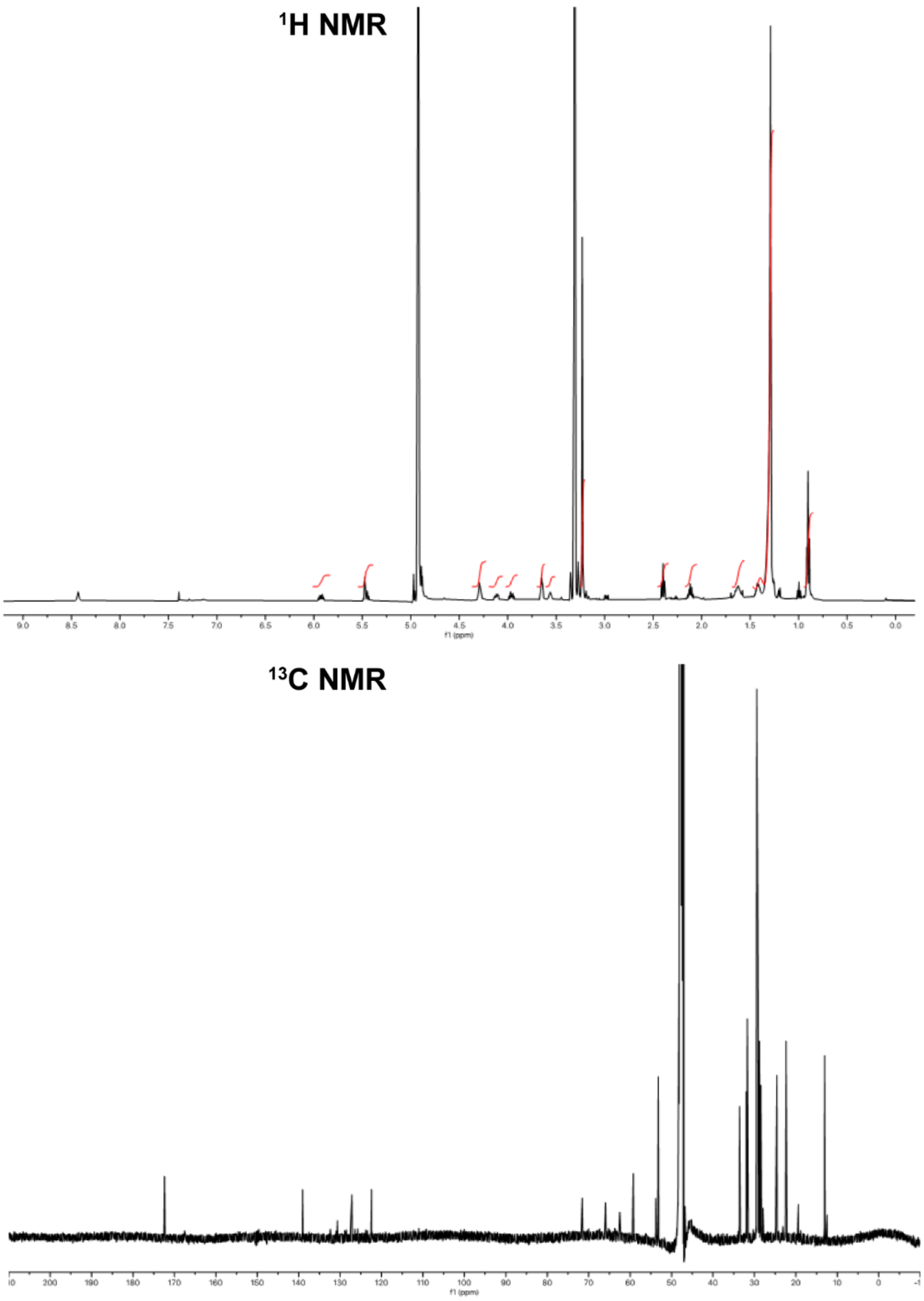


$^1$H (*top*) and $^{13}$C (*bottom*) NMR spectra of compound **O-SM$_{12:0}$**.

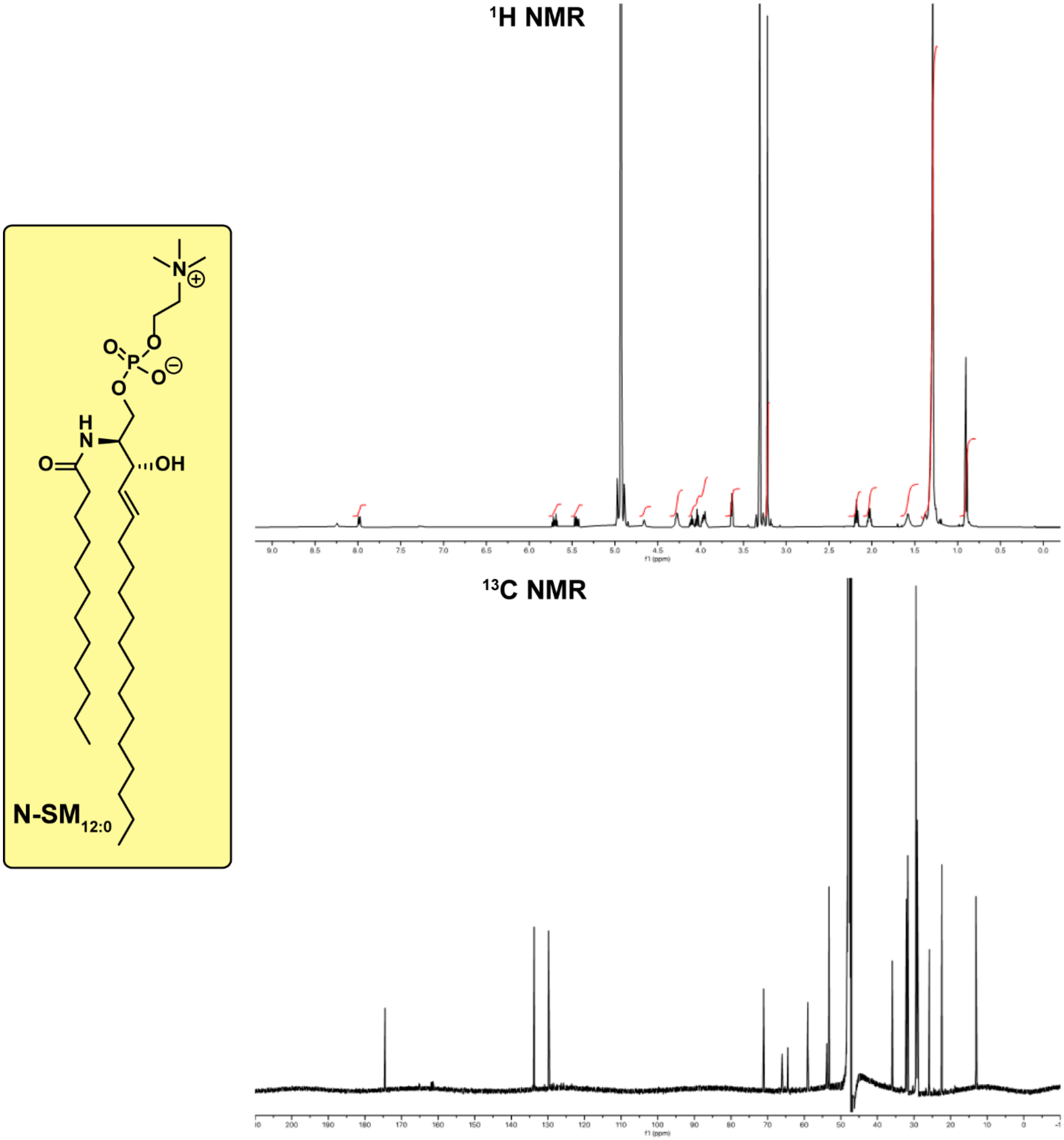


$^{1}$H (*top*) and $^{13}$C (*bottom*) NMR spectra of compound **N-SM$_{12:0}$**.

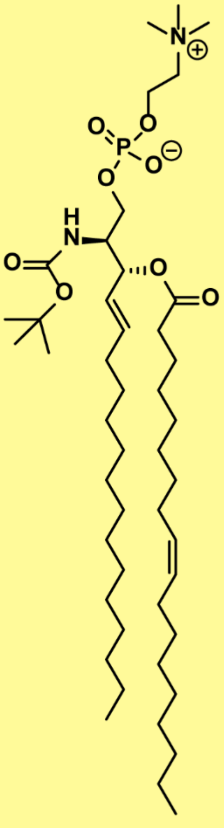

O-$SM_{18:1}$(Boc)
(**1.3**)

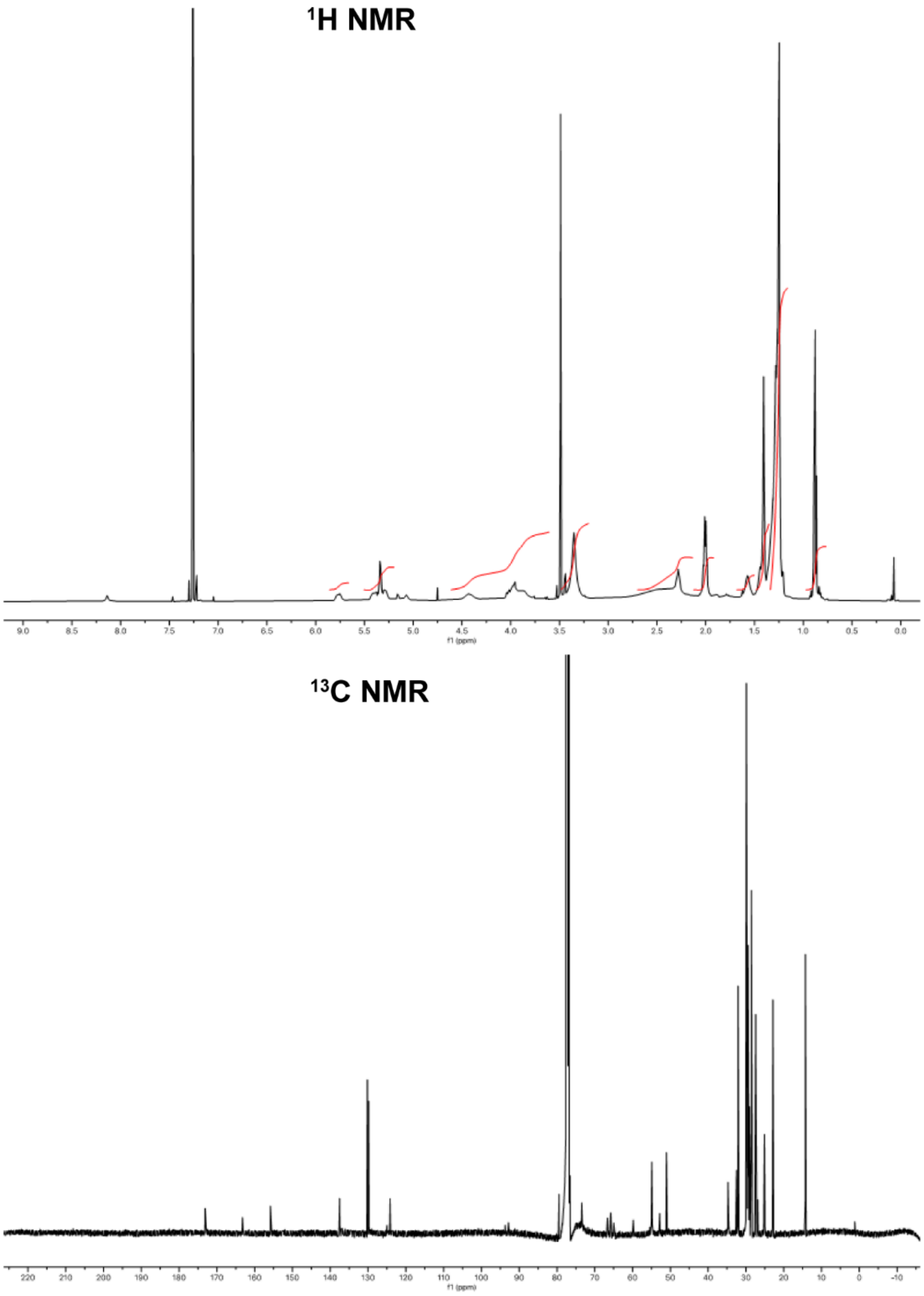


$^{1}$H (*top*) and $^{13}$C (*bottom*) NMR spectra of compound **1.3**.

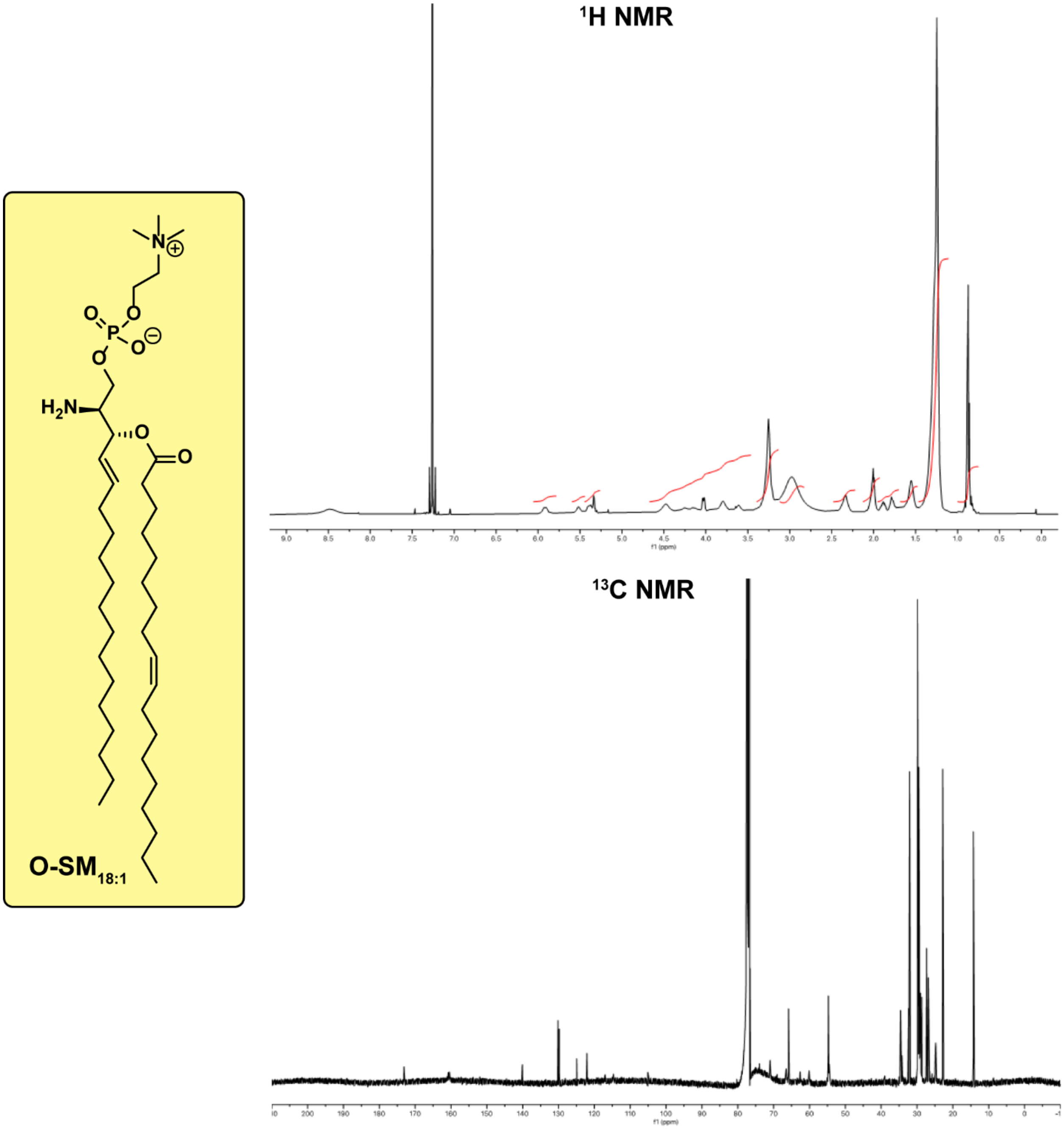


$^{1}$H (*top*) and $^{13}$C (*bottom*) NMR spectra of compound **O-SM$_{18:1}$**.

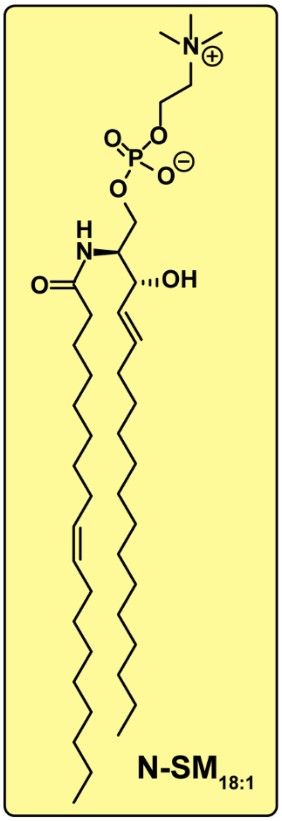


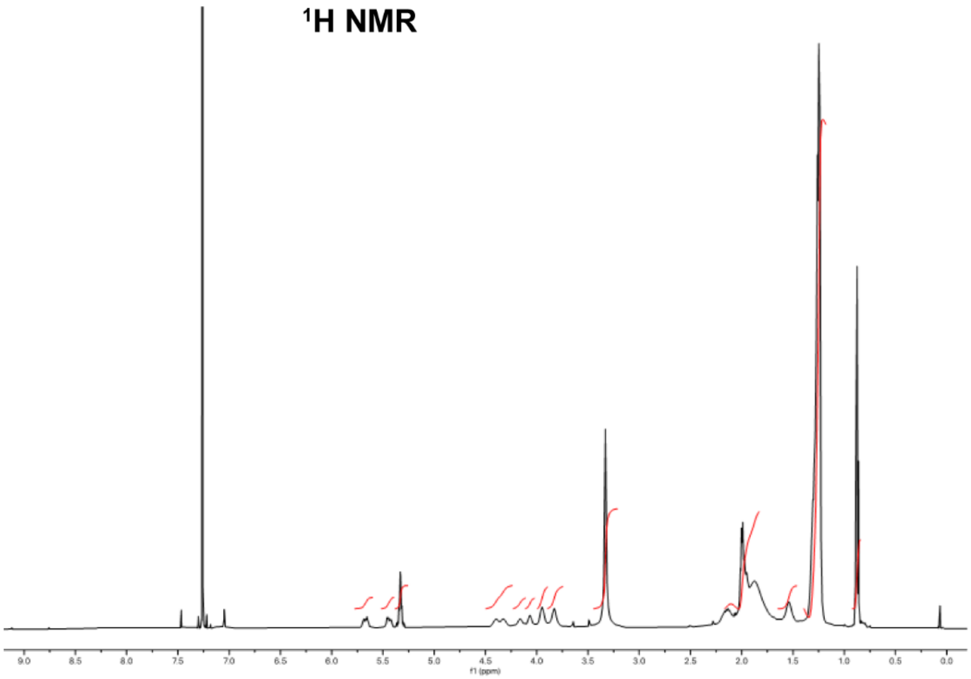


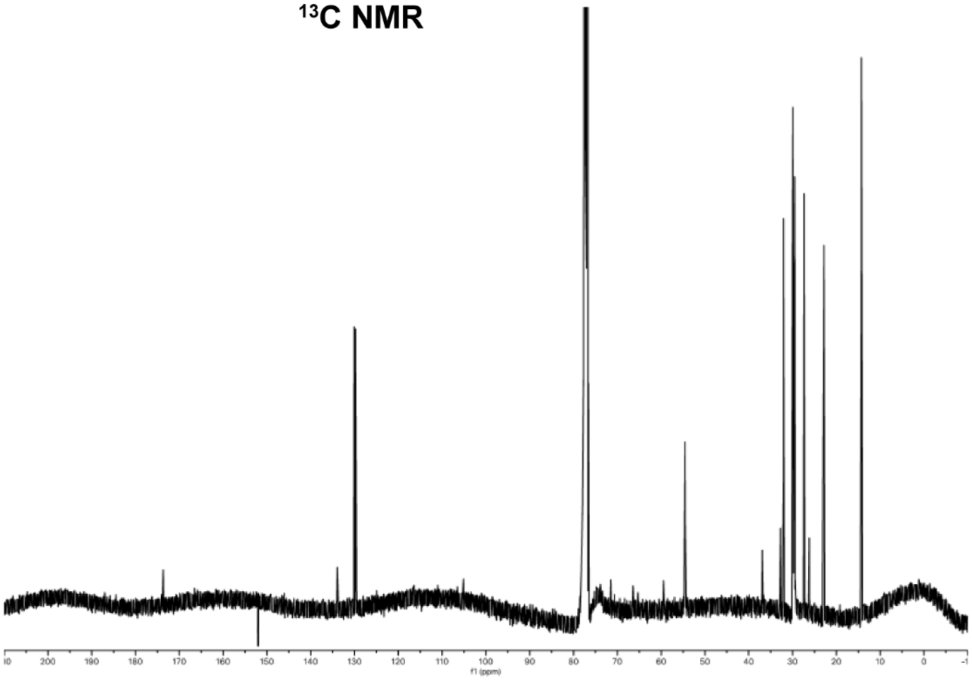


$^{1}H$ (*top*) and $^{13}C$ (*bottom*) NMR spectra of compound **N-SM$_{18:1}$**.

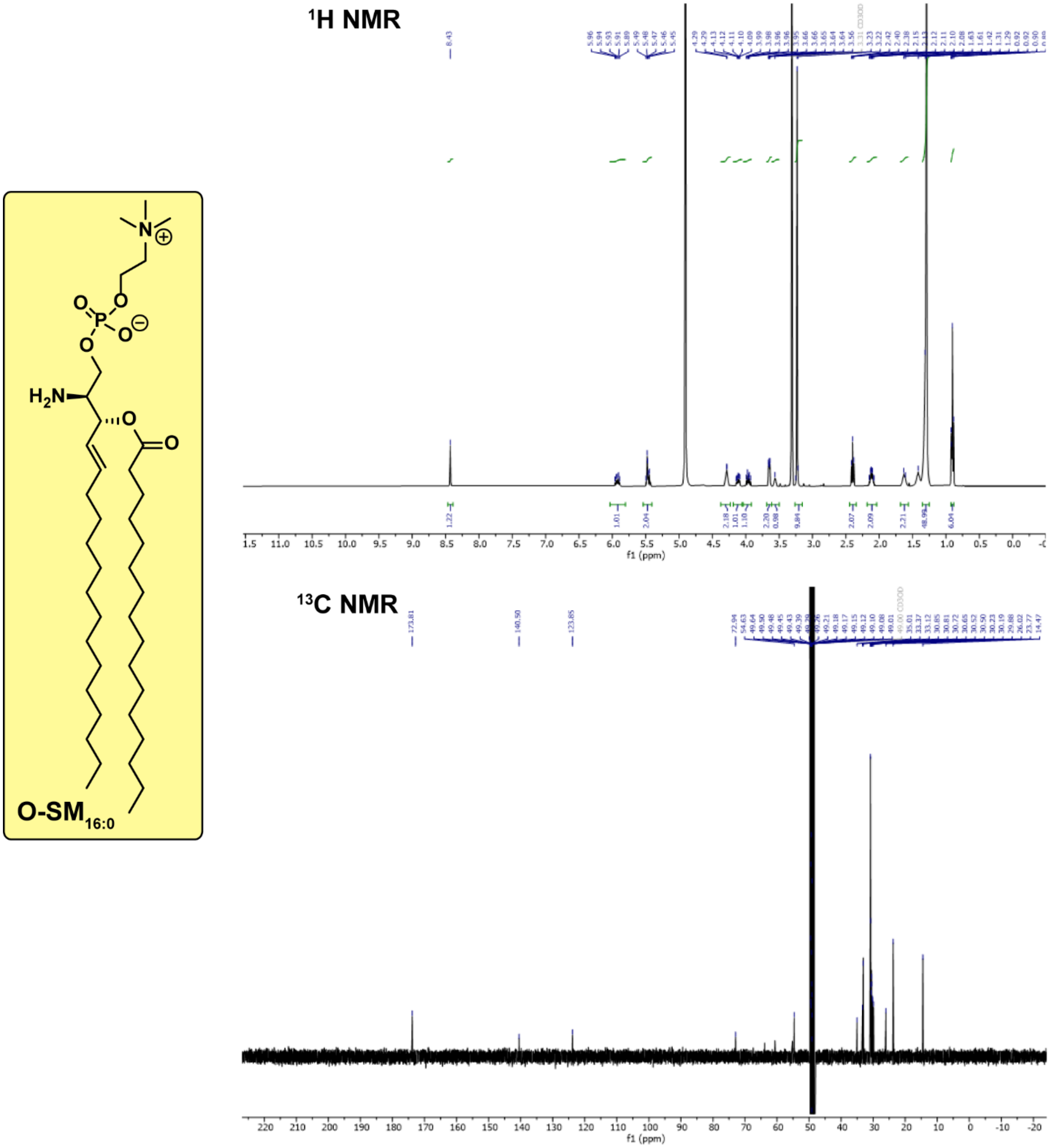


$^{1}$H (*top*) and $^{13}$C (*bottom*) NMR spectra of compound **O-SM$_{16:0}$**.

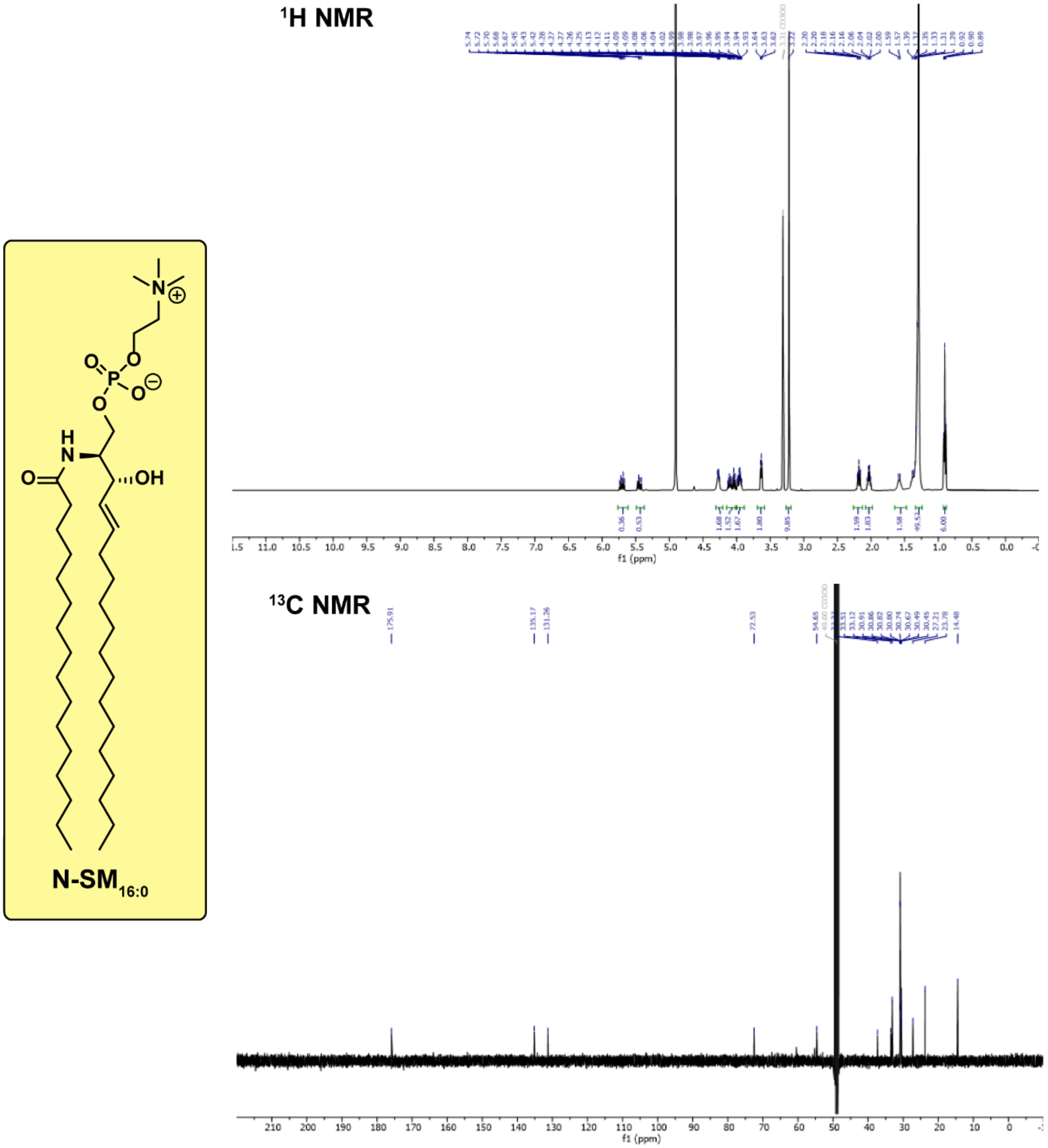


$^{1}$H (*top*) and $^{13}$C (*bottom*) NMR spectra of compound **N-SM$_{16:0}$**.

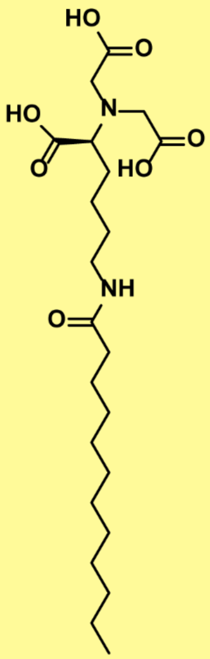

**DAN**

**$^1$H NMR**

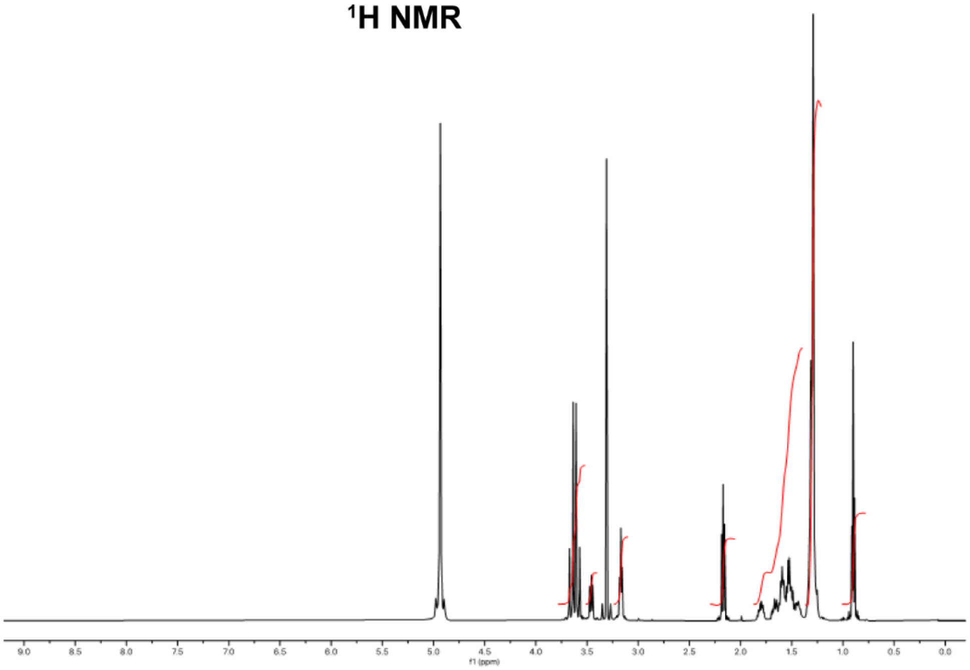

**$^{13}$C NMR**

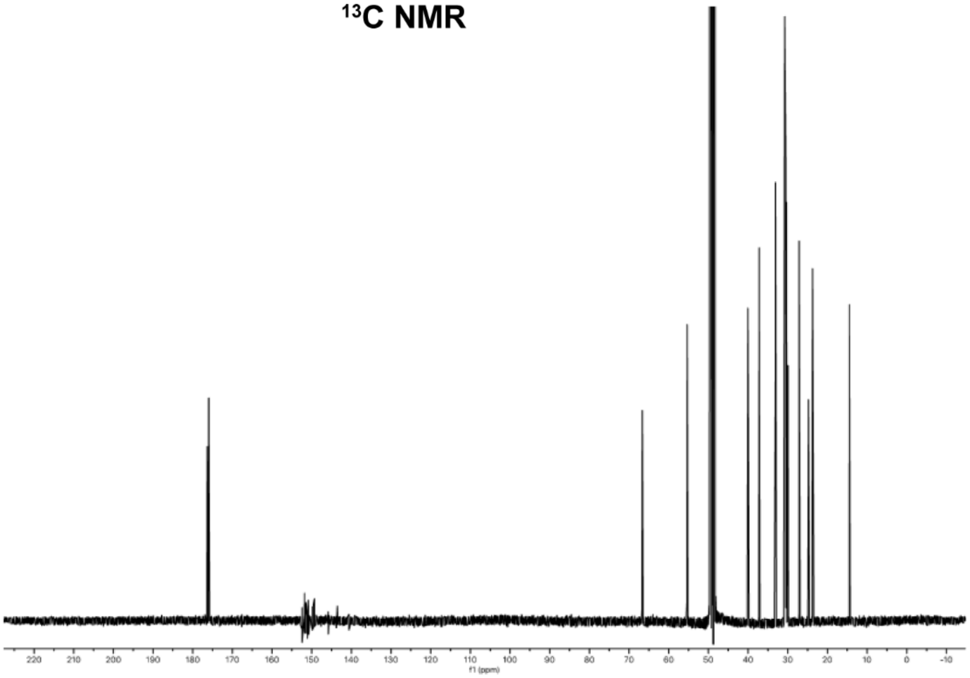

$^1$H (*top*) and $^{13}$C (*bottom*) NMR spectra of compound **DAN**.
